# Goose grubbing and warming suppress summer net ecosystem CO_2_ uptake differentially across high‐Arctic tundra habitats

**DOI:** 10.1002/ecy.4498

**Published:** 2024-12-09

**Authors:** Matteo Petit Bon, Karen H. Beard, Kari Anne Bråthen, Hanna Lee, Ingibjörg S. Jónsdóttir

**Affiliations:** ^1^ Department of Arctic Biology The University Centre in Svalbard (UNIS) Longyearbyen Norway; ^2^ Department of Wildland Resources, Quinney College of Natural Resources and Ecology Center Utah State University Logan Utah USA; ^3^ Department of Arctic and Marine Biology, Faculty of Biosciences, Fisheries, and Economics Arctic University of Norway Tromsø Norway; ^4^ Department of Biology, Faculty of Natural Sciences Norwegian University of Science and Technology Trondheim Norway; ^5^ Institute of Life and Environmental Sciences University of Iceland Reykjavik Iceland

**Keywords:** CO_2_ fluxes, ecosystem respiration (ER), environmental changes, gross ecosystem productivity (GEP), herbivore disturbance, international tundra experiment (ITEX), net ecosystem exchange (NEE), normalized‐difference vegetation index (NDVI), pink‐footed geese (*Anser brachyrhynchus*), plant communities, Svalbard

## Abstract

Environmental changes, such as climate warming and higher herbivory pressure, are altering the carbon balance of Arctic ecosystems; yet, how these drivers modify the carbon balance among different habitats remains uncertain. This hampers our ability to predict changes in the carbon sink strength of tundra ecosystems. We investigated how spring goose grubbing and summer warming—two key environmental‐change drivers in the Arctic—alter CO_2_ fluxes in three tundra habitats varying in soil moisture and plant‐community composition. In a full‐factorial experiment in high‐Arctic Svalbard, we simulated grubbing and warming over two years and determined summer net ecosystem exchange (NEE) alongside its components: gross ecosystem productivity (GEP) and ecosystem respiration (ER). After two years, we found net CO_2_ uptake to be suppressed by both drivers depending on habitat. CO_2_ uptake was reduced by warming in mesic habitats, by warming and grubbing in moist habitats, and by grubbing in wet habitats. In mesic habitats, warming stimulated ER (+75%) more than GEP (+30%), leading to a 7.5‐fold increase in their CO_2_ source strength. In moist habitats, grubbing decreased GEP and ER by ~55%, while warming increased them by ~35%, with no changes in summer‐long NEE. Nevertheless, grubbing offset peak summer CO_2_ uptake and warming led to a twofold increase in late summer CO_2_ source strength. In wet habitats, grubbing reduced GEP (−40%) more than ER (−30%), weakening their CO_2_ sink strength by 70%. One‐year CO_2_‐flux responses were similar to two‐year responses, and the effect of simulated grubbing was consistent with that of natural grubbing. CO_2_‐flux rates were positively related to aboveground net primary productivity and temperature. Net ecosystem CO_2_ uptake started occurring above ~70% soil moisture content, primarily due to a decline in ER. Herein, we reveal that key environmental‐change drivers—goose grubbing by decreasing GEP more than ER and warming by enhancing ER more than GEP—consistently suppress net tundra CO_2_ uptake, although their relative strength differs among habitats. By identifying how and where grubbing and higher temperatures alter CO_2_ fluxes across the heterogeneous Arctic landscape, our results have implications for predicting the tundra carbon balance under increasing numbers of geese in a warmer Arctic.

## INTRODUCTION

Environmental changes to the terrestrial carbon (C) cycle are concerning because of their potential to modify ecosystem C exchange rates, thus feeding back to atmospheric greenhouse gas concentrations and, ultimately, the global climate system (Luo, 2007). Of all terrestrial ecosystems, northern permafrost ecosystems only cover ~22% of the Earth's land surface (Obu et al., 2019), yet they store half of the global belowground organic C pool (Mishra et al., 2021), or twice as much C as is presently in the atmosphere (Schuur et al., 2015). These ecosystems therefore play a key role in the global C cycle (Schimel et al., 2015) and the major positive ecosystem‐climate change feedback loop (Schuur et al., 2015). There has never been a more urgent need to better understand the sensitivity and magnitude of their CO_2_‐flux responses to environmental changes (Schuur et al., 2022; Virkkala et al., 2018).

With the Arctic warming three to four times faster than the rest of the planet (Rantanen et al., 2022), evidence is accumulating that elevated temperatures are altering the C balance of tundra ecosystems (Schuur et al., 2022; Virkkala et al., 2018). However, studies on the effect of summer warming on net CO_2_ exchange rate (net ecosystem exchange, NEE) have unveiled contrasting responses. Some studies have reported net CO_2_ losses (ecosystems act as net C sources; e.g., Belshe et al., 2013), indicating that warming‐induced increases in ecosystem respiration (ER) may outbalance increases in gross ecosystem productivity (GEP). Conversely, some other studies have documented net CO_2_ gains (ecosystems act as net C sinks; e.g., McGuire et al., 2012). The Arctic tundra is a mosaic of different habitats and plant communities, which differ in C dynamics (Arndal et al., 2009; Sjögersten et al., 2006), plausibly manifesting differential CO_2_‐flux responses to higher temperatures. For example, in a high‐Arctic Canadian ecosystem, experimental warming increased net CO_2_ uptake in drier habitats, but reduced it in wetter habitats (Welker et al., 2004). Contrastingly, in the northern Alaskan tundra, Oberbauer et al. (2007) found warming to enhance net CO_2_ losses from drier habitats and net CO_2_ gains in wetter habitats. Further, when measured across a fine‐scaled soil moisture gradient in an alpine meadow, positive responses of soil ER (and plant biomass) to warming were greater in wetter conditions (Fei et al., 2015). This is consistent with the fact that both tundra ER and GEP generally increase with soil moisture and temperature, although ER (not GEP) might decrease when soil moisture exceeds certain thresholds (Knowles et al., 2015; Sjögersten et al., 2006). Combined, these results suggest that better predicting the C balance of a warmer Arctic relies on understanding how warming effects may vary across the heterogeneous tundra landscape (Virkkala et al., 2018).

Recently, the crucial role of vertebrate herbivores in regulating ecosystem functioning and the C balance of tundra ecosystems has been recognized (Barbero‐Palacios et al., 2024; Cahoon et al., 2012; Leffler et al., 2019; Petit Bon, Hansen, et al., 2023; Ylänne et al., 2015). During the last five decades, the number of migratory, Arctic‐breeding geese has risen considerably across several regions (Fox & Madsen, 2017; but see Weegman et al., 2022). After arrival in spring and prior to vegetation greening, geese belonging to the genera *Anser* and *Chen* forage through grubbing (i.e., by excavating belowground parts of vascular plants; Fox et al., 2006). Grubbing, which is most pronounced in wetter habitats (Eischeid et al., 2023; Speed et al., 2009), largely depletes plant biomass, and hence plant C and nutrient pools (Petit Bon, Bråthen, et al., 2023), and disturbs the soil (Jefferies & Rockwell, 2002; Ravolainen et al., 2020), likely reducing net C uptake. The only research on the impact of grubbing on tundra CO_2_ fluxes investigated peak summer responses in wet habitats (Van der Wal et al., 2007). They found that both GEP and ER were reduced by grubbing. Yet, plausibly because of the significant decrease in plant biomass, the reduction in GEP exceeded that in ER, thereby weakening ecosystem C gains. However, grubbing occurs at the onset of the plant growing season, and the tundra can exhibit different susceptibility to grubbing, with drier habitats displaying slower recovery rates (Speed et al., 2010). Hence, investigations of grubbing impacts on early summer CO_2_ fluxes across the range of habitats used by geese are warranted. Moreover, whether spring goose grubbing can modulate tundra CO_2_‐flux responses to summer warming, as has been demonstrated for aboveground goose grazing (Leffler et al., 2019; Sjögersten et al., 2008), remains to be investigated.

The archipelago of Svalbard, in the European high Arctic, is one of the most rapidly warming regions on Earth (Isaksen et al., 2022). Due to a mixture of anthropogenic factors, the Svalbard‐breeding pink‐footed goose (*Anser brachyrhynchus*) population has grown strikingly from ~15,000 individuals to ~90,000 individuals over the past 50 years (Fox & Madsen, 2017). This population growth has the potential to cause greater disturbance to the tundra through grubbing (Pedersen, Speed, & Tombre, 2013) and greater exploitation of previously less‐used drier habitats (Eischeid et al., 2023; Pedersen, Tombre, et al., 2013). Therefore, Svalbard represents a highly relevant system to deepen our knowledge on how grubbing and warming, alone and in combination, can modify ecosystem CO_2_ fluxes across the tundra landscape.

In this study, we asked to what extent goose grubbing in spring and elevated temperature throughout the summer alters high‐Arctic Svalbard ecosystem CO_2_ fluxes during the growing season. We simulated these key environmental‐change drivers over two years in a full‐factorial field experiment across three habitats (mesic, moist, and wet habitats) that differ in soil moisture and plant‐community composition and that are widely used by pink‐footed geese in spring. Based on the outline presented above, we expected (1) grubbing to decrease GEP more than ER, especially in early summer, resulting in a reduced C uptake by the ecosystem; (2) higher temperatures to increase GEP and ER to a comparable extent, with larger alterations in mid‐to‐late summer, resulting in little change in NEE; (3) grubbing and warming effects to partly offset each other, given their predicted contrasting effects when acting alone (cf. 1 vs. 2); and (4) either driver to be stronger modifiers of CO_2_ fluxes in mesic than wet habitats, reflecting the lower resistance of drier habitats to grubbing and the fact that soil moisture in mesic habitats is unlikely to constrain responses to temperature.

## MATERIALS AND METHODS

### Study area and experimental design

**FIGURE 1:**
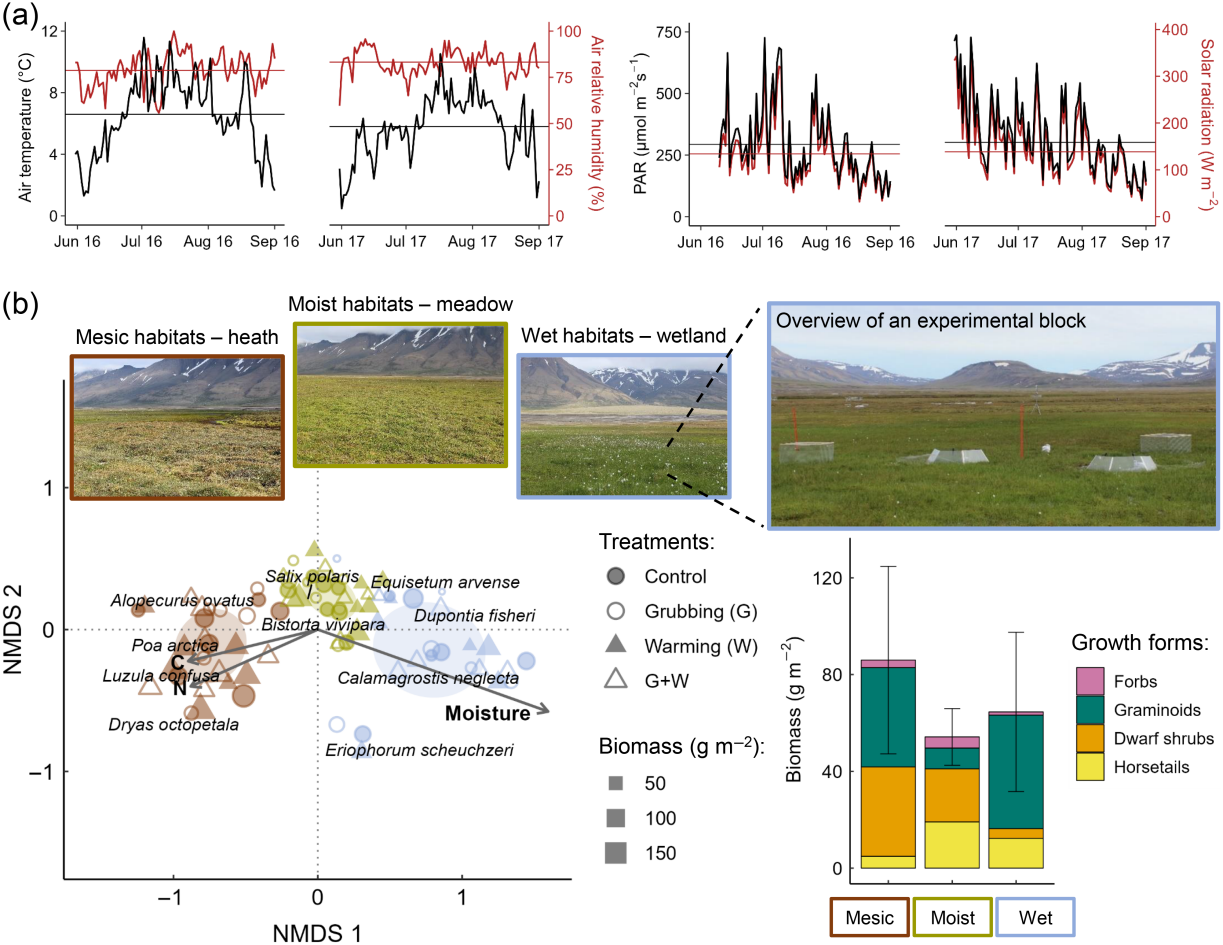
Environmental characteristics during the experiments and overview of the three studied habitats and their vascular plant communities. (a) Daily average air temperature and air relative humidity (left panel) and photosynthetically active radiation (PAR) and solar radiation (right panel) from June to August. Horizontal lines show the averages across the summer. Data were registered every 15 min at 2 m height by an in situ weather station. (b—left panel) Nonmetric multidimensional scaling on Bray–Curtis dissimilarity distances of the plant species composition of experimental plots (*n* = 80; see main text) at peak growing season in 2017 (stress = 0.12; nonmetric fit *R* ^2^ = 0.99; linear fit *R* ^2^ = 0.94). Data (from Petit Bon et al., 2021; Petit Bon, Bråthen, et al., 2023) were analyzed in R v. 4.3.0 with the package “vegan” (Oksanen et al., 2020). Only the 10 most abundant species, making up >90% of the aboveground biomass within plots, are shown (names follow the Svalbard Flora; https://www.svalbardflora.no). Ellipses are the 95% confidence intervals of habitat centroids (permutational‐ANOVA: *R* ^2^ = 0.57, *p* < 0.0001). The four experimental treatments (see text) are displayed, with dot size proportional to plot biomass. Fit of the soil parameters when regressed on the biplot is moisture: *r* ^2^ = 0.80, *p* = 0.0001; nitrogen (N) concentration: *r* ^2^ = 0.26, *p* = 0.0041; carbon (C) concentration: *r* ^2^ = 0.24, *p* = 0.0041; details in Appendix S1: Section S1. (b—right panel) Average (±SD) aboveground plant biomass in control plots of the three habitats, sorted according to growth forms (data from references above). Photo credits: Matteo Petit Bon.

Research was performed in Svalbard, and experiments took place during summers of 2016 and 2017 in Adventdalen (78°10′ N, 16°05′ E), an U‐shaped 2‐ to 4‐km‐wide formerly glaciated valley, at 15–60 m above sea level. Adventdalen is in the bioclimatic subzone C (Middle Arctic tundra), the warmest in the high Arctic, which overall comprises ~23% of the non‐glaciated Arctic (Walker et al., 2005). Mean annual and summer (June–August) temperatures for the 30‐year period 1988–2017 were −4.2 and 5.4°C, respectively, while mean annual precipitation was 200 mm (data from Longyearbyen airport weather station, ~10 km from the study site; http://met.no; Figure 1a and Appendix S1: Figure S1).

In late summer of 2015, we selected seven replicate sites (~300–1500 m apart), each including three habitats (~30–100 m apart) with contrasting soil moisture, plant‐community composition, and plant biomass: mesic (heath vegetation), moist (meadow vegetation), and wet (wetland vegetation) habitats (Figure 1b). These three habitats are widely used by pink‐footed geese (Fox et al., 2006) and are common across Svalbard (Johansen et al., 2012). Furthermore, tundra heaths, meadows, and wetlands are widespread communities across the Circumpolar Arctic, covering ~16%, ~23%, and ~6% (~45% in total), respectively, of the Middle Arctic tundra (Walker et al., 2005).

To study the effects of spring goose grubbing and summer warming on ecosystem CO_2_ fluxes, we used a full‐factorial randomized‐block design. At each site, a block with four 80 cm × 80 cm plots (~2–10 m apart) was established within each of the three habitats (Figure 1b) immediately after snowmelt in late May 2016. Due to a flooding event in early June 2016, one block in wet habitats was discarded, reducing the total number of plots from 84 to 80. Plots within blocks were assigned randomly to one of four treatment combinations: no grubbing and ambient temperature (unmanipulated control); grubbing and ambient temperature; no grubbing and warming; grubbing and warming. To avoid herbivory beyond experimental grubbing, all plots were fenced during both summers (net mesh size: 1.9 cm × 1.9 cm).

Spring grubbing was simulated once each year when grubbing was most intensive (5–22 June) by using a steel tube (2 cm diameter) that was forced to a depth of 5 cm and twisted to remove material from the plot (Speed et al., 2010). Grubbing was applied in a regular fashion to 33% of the plot surface (Appendix S1: Figure S2), after which we added 120 g of fresh goose feces to the “grubbing” and “grubbing and warming” plots (Petit Bon et al., 2021, 2023). We found that the effect of our grubbing treatment mirrored that of natural grubbing across all three habitats (see below), consistent with observations of recent increases in grubbing intensity in previously less‐used drier habitats (Eischeid et al., 2023; Pedersen, Tombre, et al., 2013). Simulating a similar grubbing intensity allowed the comparison between sensitivity and magnitude of CO_2_‐flux responses across habitats. Yet, the magnitude of our spring grubbing was most comparable with that of natural grubbing observed in moist habitats, and somewhat lower and higher than that of natural grubbing observed in wet and mesic habitats, respectively.

Summer warming was achieved with hexagonal, ITEX‐style open‐top chambers (OTCs, 1.4 m base diameter; Henry et al., 2022), which we deployed soon after snowmelt (5–15 June) and removed before the winter (1–10 October). Across years and habitats, OTCs in “warming” plots increased daily (from 900 to 1700 h) average temperatures in air (+10 cm above the moss‐mat), within the moss‐mat (−2 cm) and in soil (−7 cm) by ~1.1, ~1.0, and ~0.3°C, respectively, between 15 June and 31 August. Temperatures were recorded in each “treatment × habitat” combination (n replicates = 3 to 4 plots; loggers: DS1921G‐F5; Maxim Integrated, San Jose, California, USA; and U23‐003/UA‐001; Onset Computer Corporation, Bourne, Massachusetts, USA) (Appendix S1: Figures S3–S5).

We determined to what extent our grubbing simulation reflected natural grubbing by geese. In the spring of 2017, we established 36 plots within naturally grubbed areas at three of the seven sites by identifying four plots (25 cm × 25 cm; ~2–10 m apart) in each habitat as close as possible to the experimental blocks (Appendix S1: Figure S2).

### Data collection and processing

To capture the impact of grubbing and warming on ecosystem CO_2_ fluxes throughout the plant growing season, data were gathered at each of three sampling occasions (early, peak, and late summer; cf. Cannone et al., 2019) in both years (early [2016: 21–30 June; 2017: 29 June–5 July], peak [2016: 21–29 July; 2017: 21–28 July], and late [2016: 10–18 August; 2017: 15–22 August] summer). Differing dates of sample collection in early summer among years were due to the unusually warm 2016. Although the landscape thaws patchily, focal habitats reach peak biomass at approximately the same time (second half of July; Van der Wal & Stien, 2014). We sampled each of the seven sites within one day, thereby each round of data collection across the whole experiment required seven days. The mean interval between consecutive sampling occasions was 15.7 ± 3.7 SD days. Consequently, our data encompass a relatively large cross section of biotic and abiotic conditions featuring the short high‐Arctic summer (~25% of a ~2.5‐month Svalbard growing season).

Ecosystem CO_2_ fluxes were assessed using a closed system (Virkkala et al., 2018) made of a clear acrylic chamber (25 cm × 25 cm area × 35 cm height), including a fan for air mixing, connected through an air pump (L052C‐11; Parker Corp, Cleveland, Ohio, USA; ~1 L min^−1^ flow rate) to a CO_2_ infrared gas analyzer (LI‐840A; LICOR, Lincoln, Nebraska, USA). To limit air circulation between chamber and external environment, sealing was obtained using a flexible plastic skirt attached to the bottom of the chamber and held down during measurements using a 4‐kg 2‐m‐long steel chain (cf. Jónsdóttir et al., 2022; Petit Bon, Hansen, et al., 2023).

To include some of the spatial variation within experimental plots, CO_2_‐flux data were gathered in two 25 cm × 25 cm subplots (>30 cm apart) at each plot. Subplots were laid out at random in 2016 and used for data collection throughout both summers. In 2017, data collection was also performed within naturally grubbed plots, following sampling schedule and methods applied to experimental plots.

A set of CO_2_‐flux measurements was taken (between 900 and 1700 h) in each subplot and sampling occasion during both summers (*n* set = 1068). Each set consisted of a light and a dark measurement, from which we obtained NEE and ER, respectively. GEP was obtained by subtracting ER from NEE. For ER measurements, we placed a dark cloth over the chamber to exclude light. Photosynthetically active radiation (PAR) and temperature can exhibit substantial diurnal variations in the Arctic (cf. Sjögersten et al., 2006, 2008). To reduce potential within‐day variations in environmental conditions among treatments, all measurements within a block (i.e., habitat) were collected in sequence. Moreover, at each site and for each measurement day, we approached the three blocks and the plots (i.e., treatments) within blocks in a random order to avoid introducing systematic measurement differences across habitats and treatments. Our sampling design successfully minimized diurnal variations in environmental conditions across treatment and habitat measures (see *Statistical analysis* for details).

During both light and dark measurements, CO_2_ concentration within the chamber was sampled every second and each 5‐s average was recorded for 120–180 s. Together with CO_2_ concentration, we recorded within‐chamber PAR and air temperature 30 cm from the ground every 5 and 10 s, respectively, using a PAR sensor connected to a datalogger (LI‐190SA and LI‐1400, LICOR) and a temperature logger (DS1922L‐F5, Maxim Integrated). We calculated CO_2_ fluxes for each measurement by fitting linear regression models based on the ideal gas law (Jónsdóttir et al., 2022; Petit Bon, Hansen, et al., 2023). We used within‐chamber average air temperature and average air pressure recorded at Adventdalen weather station (~2 km from the study site; sampling interval: 1 s). Because measurements took place ±4 h of solar noon, our results are indicative of maximum ecosystem sink strength.

At the plot level, temperatures (air, moss‐mat, and soil) during CO_2_‐flux measurements were obtained through date/time interpolation using “treatment × habitat” average temperatures recorded by our loggers. Finally, we measured plot volumetric soil moisture content (integrated across 0–10 cm depth) at each sampling occasion by averaging readings from five random spots in each plot (ML3 Theta Kit; Delta‐T Ltd., Cambridge, UK).

To build a link between treatment‐induced alterations in ecosystem CO_2_ fluxes and vegetation, we measured the normalized‐difference vegetation index (NDVI). NDVI is a proxy for net primary productivity (photosynthesis), as well as live vegetation cover or live aboveground biomass (Boelman et al., 2003; Jespersen et al., 2023; Appendix S1: Figure S6). NDVI was determined in each subplot at each sampling occasion using a handheld NDVI meter (2‐channel sensor SKL925 SpectroSense2 and SKR 1800/SS2; Skye Instruments, Llandrindod Wells, UK) mounted on a pole at the height ensuring a ground projection with diameter equals to the diagonal of the subplots.

### Statistical analysis

To determine whether the effect of simulated grubbing reflected that of natural grubbing, we compared CO_2_‐flux variables (NEE, GEP, and ER) and NDVI among experimentally grubbed, naturally grubbed, and ungrubbed control plots. Relative to controls, the impact of simulated grubbing was either similar or weaker (based on our directional hypotheses) than that of natural grubbing (Appendix S1: Figure S7). Therefore, we concluded that our manipulation satisfactorily mirrored and, if anything, underestimated the effects of natural grubbing.

All analyses on the effects of experimental spring grubbing and summer warming used a linear mixed‐effects model (LMM) framework. For each response variable (NEE, GEP, ER, and NDVI), separately for the two years (2016 and 2017) and the three habitats (mesic, moist, and wet), we fitted LMMs in which the initial full fixed‐effects structure included the three‐way interaction among the three categorical predictors: “grubbing” (grubbed and ungrubbed plots), “warming” (warmed and ambient plots), and “seasonality” (early, peak, and late summer). Because of the high variability around the relationships between CO_2_ fluxes and either PAR or air temperature (Appendix S1: Figure S8), we did not standardize CO_2_‐flux data at a fixed level of these variables (cf. Falk et al., 2015). Nonetheless, within each year and sampling occasion, differences in PAR and air temperature among treatment and habitat measurements were small (Appendix S1: Figures S9 and S10), whereas differences between and within growing seasons are assumed to represent natural variations in abiotic conditions (cf. Sjögersten et al., 2008). We specified “block,” “plot‐within‐block,” and “subplot‐within‐plot” as nested random‐effect intercept terms. As we sampled each site within one day, “block” also accounted for temporal variations among consecutive sampling days.

For each full LMM, we separately selected the better random‐effects structure by deleting those terms with an estimated zero variance (Bates, Kliegl, et al., 2015). Then, by using ANOVA with threshold at *p* < 0.05 (Bolker et al., 2009), we selected the most parsimonious, but common, fixed‐effects structure for all the analyses. We kept a common model structure to be able to compare (1) effect sizes of CO_2_‐flux (NEE, GEP, and ER) responses within and across habitats, as well as of one‐year (2016) and two‐year (2017) responses, and (2) CO_2_‐flux and NDVI responses. The final model structure included all three main effects and the two‐way “grubbing × seasonality” and “warming × seasonality” interactions. Further details on data analyses are reported in Appendix S1: Section S2, while an overview of the final dataset used is presented in Appendix S1: Table S1.

To gain a better mechanistic understanding of the biotic and abiotic controls of CO_2_ fluxes in this high‐Arctic ecosystem, we explored across‐habitat relationships between NEE, ER, or GEP (used as response variables in separate models) and both NDVI and abiotic (soil moisture and temperature) variables (used as additive smooth fixed effects) using additive mixed‐effects models (AMM), separately for the two years. We used AMM to enhance the flexibility of the modeled relationships and to be able to display the underlaying nonlinear patterns that LMM would have missed. In these models, we did not incorporate treatments as fixed effects as they would be correlated with the considered smooth terms (cf., e.g., the effect of treatments on NDVI). The initial random‐effects structure of these models, which was also simplified as outlined above, included “site,” “block‐within‐site,” “plot‐within‐block,” and “subplot‐within‐plot” as nested random‐effect smooth terms (Appendix S1: Section S2).

We validated each final model by checking for normal distribution of the residuals, homogeneity of residual variances, and linearity between observed and fitted values. We focus on presenting and discussing two‐year responses to treatments. One‐year responses are also presented but displayed in Appendix S1. Analyses were run in R v. 4.3.0 (https://www.r-project.org) with packages “lme4” (LMM fitting; Bates, Mächler, et al., 2015), “mgcv” (AMM fitting; Wood, 2017), “emmeans” (model summaries and factor‐level contrasts; Lenth, 2021), and “ggplot2” (graphical displays; Wickham, 2016).

## RESULTS

**FIGURE 2:**
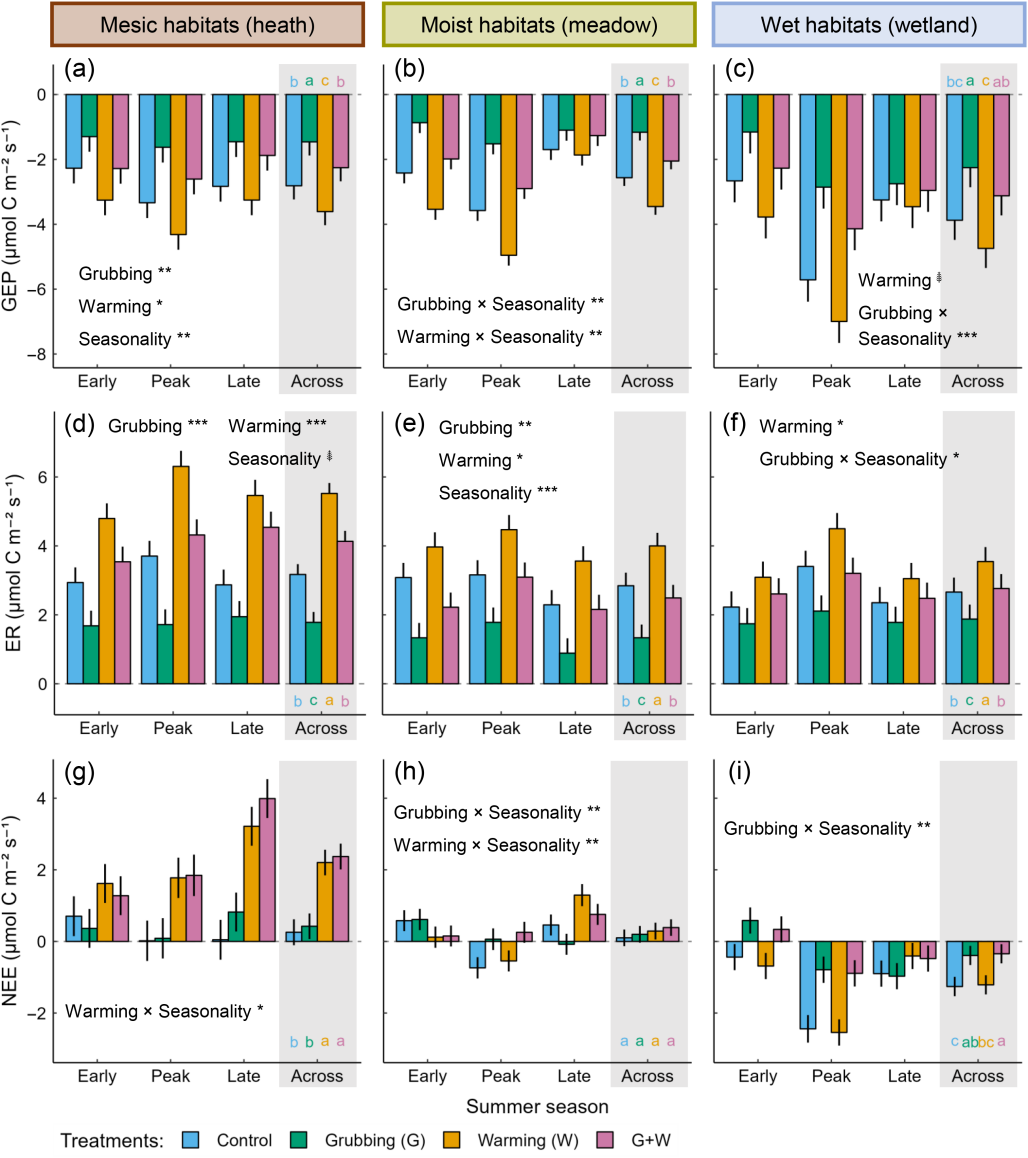
Effects of spring goose grubbing and summer warming on ecosystem CO_2_ fluxes in 2017. Model predictions ± SE for (a–c) gross ecosystem productivity (GEP), (d–f) ecosystem respiration (ER), and (g–i) net ecosystem exchange (NEE) in early, peak, and late summer, separately for the three habitats. Gray panels show model predictions ± SE averaged over the summer; different letters indicate significant differences among treatments. Significant and marginally significant main and interactive effects are shown (ANOVA); when an interaction was significant (*p* < 0.05), its main effects are not shown. Significance: 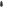 *p* < 0.1; *p < 0.05; **p < 0.01; and ***p < 0.001. Full ANOVA results are given in Appendix S1: Table S2. LMM parameter estimates are given in Appendix S1: Tables S3–S5. Positive and negative fluxes denote CO_2_ losses (the ecosystem acts as a C source) and CO_2_ gains (the ecosystem acts as a C sink), respectively.

Background (i.e., unmanipulated control plot) CO_2_ fluxes in 2017 varied among the three habitats (Figure 2). Throughout the growing season, GEP was greatest in wet habitats (−3.9 μmol CO_2_ m^−2^ s^−1^) and similar in mesic and moist habitats (ca. −2.7 μmol CO_2_ m^−2^ s^−1^), while ER was similar among habitats (2.7–3.2 μmol CO_2_ m^−2^ s^−1^). Across habitats, both GEP and ER were greatest at peak summer, especially GEP in wet habitats. Over the growing season, mesic and moist habitats were weak CO_2_ sources (0.1–0.3 μmol CO_2_ m^−2^ s^−1^), while wet habitats were CO_2_ sinks (−1.3 μmol CO_2_ m^−2^ s^−1^). Similar patterns in CO_2_ fluxes held in 2016, although in wet habitats CO_2_ uptake was ~50% greater than in 2017, owing to larger GEP but similar ER (Appendix S1: Figure S11).

### Effects of goose grubbing and warming on CO_2_ fluxes

Overall, spring goose grubbing decreased and summer warming increased both GEP (Figure 2a–c) and ER (Figure 2d–f), resulting in similar GEP and ER fluxes between control plots and grubbed and warmed plots. Nonetheless, as grubbing decreased GEP more than ER and warming increased ER more than GEP, both drivers alone and in combination still reduced net ecosystem CO_2_ uptake (Figure 2g–i). Moreover, because GEP and ER showed habitat‐specific responses to treatments, the impacts of grubbing and warming on NEE also varied across habitats. One‐year (2016) responses to treatments (Appendix S1: Figure S11) were similar to two‐year (2017) responses (Figure 2), although generally weaker.

#### Mesic habitats

Warming stimulated summer‐long GEP by 30% (Figure 2a) and ER by 75% (Figure 2d), thus promoting a 7.5‐fold increase in the overall ecosystem C source strength (Figure 2g). This increase was mainly driven by large changes in peak and late summer NEE. Grubbing reduced growing‐season GEP and ER by ~45% (Figure 2a,d), without modifying NEE (Figure 2g).

In 2016 (Appendix S1: Figure S11), warming stimulated early summer GEP and ER and late summer ER, which translated to a 35% increase in summer‐long ER and no changes in summer‐long GEP. These produced a 1.5‐fold rise in the overall ecosystem C source strength. Throughout the summer, grubbing reduced GEP by 25%, tended to reduce ER (−15%), and did not affect NEE.

#### Moist habitats

Grubbing suppressed (−60%) and warming stimulated (+40%) early and peak summer, but not late summer, GEP, leading to a 55% decrease (under grubbing) and a 35% increase (under warming) in summer‐long GEP (Figure 2b). Concurrently, grubbing suppressed (−55%) and warming increased (+35%) growing‐season ER (Figure 2e). As overall changes in GEP and ER canceled each other out, summer‐long NEE was not altered by treatments (Figure 2h). Yet, grubbing still shifted moist habitats in peak summer from C sink (−0.7 μmol CO_2_ m^−2^ s^−1^) to weak C source (0.1 μmol CO_2_ m^−2^ s^−1^), while warming caused a twofold increase in their late summer C source strength.

In 2016 (Appendix S1: Figure S11), grubbing reduced growing‐season GEP and ER by 35% and 20%, respectively, while warming promoted similar responses to those detected after two years. Although only marginally significant, grubbing shifted the growing‐season C balance of moist habitats from weak C sink (−0.1 μmol CO_2_ m^−2^ s^−1^) to C source (0.5 μmol CO_2_ m^−2^ s^−1^), with no effect of warming.

#### Wet habitats

Grubbing suppressed early and peak summer, but not late summer, GEP by ~50%, leading to a 40% decrease in summer‐long GEP (Figure 2c). As grubbing reduced growing‐season ER to a lesser extent (−30%) (Figure 2f), with effects driven by a 40% ER reduction in peak summer only, the overall ecosystem C sink strength was also decreased by 70% (Figure 2i). Such reduction was driven by alterations in early and peak summer NEE. Warming raised growing‐season ER by 35% (Figure 2f), tended to stimulate (+20%) GEP (Figure 2c), and did not alter NEE (Figure 2i).

In 2016 (Appendix S1: Figure S11), grubbing suppressed growing‐season GEP by 25%, without modifying ER or NEE. Warming tended to stimulate both summer‐long GEP (+15%) and ER (+25%) and did not affect NEE.

### Effects of goose grubbing and warming on NDVI

**FIGURE 3:**
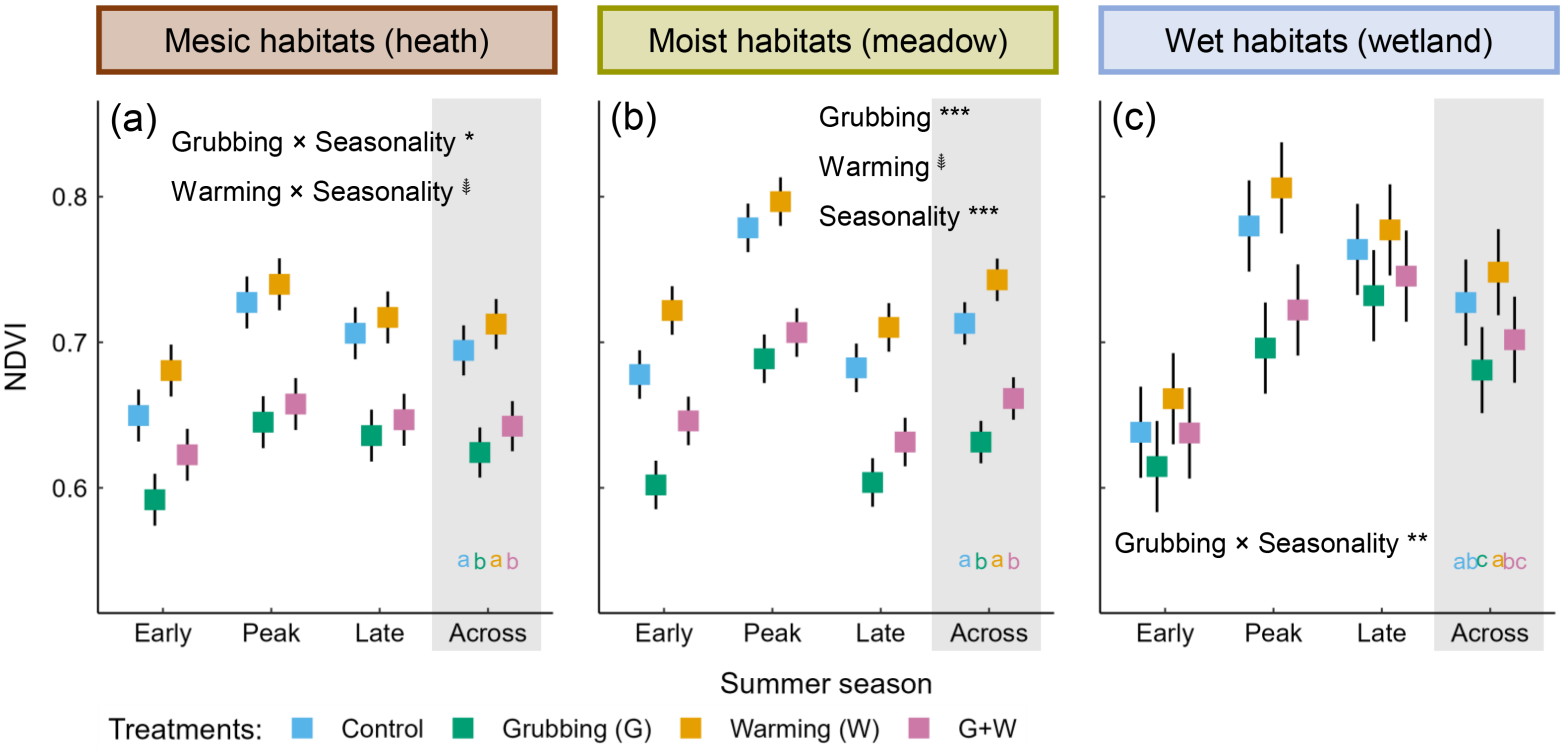
Effects of spring goose grubbing and summer warming on normalized‐difference vegetation index (NDVI) in 2017. Model predictions ± SE for NDVI of (a) mesic, (b) moist, and (c) wet habitats in early, peak, and late summer. Gray panels show model predictions ± SE averaged over the summer; different letters indicate significant differences among treatments. Significant and marginally significant main and interactive effects are shown (ANOVA); when an interaction was significant (*p* < 0.05), its main effects are not shown. Significance: 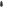 *p* < 0.1; *p < 0.05; **p < 0.01; and ***p < 0.001. Full ANOVA results are given in Appendix S1: Table S2. LMM parameter estimates are given in Appendix S1: Table S6.

Overall, grubbing had stronger effects than warming on NDVI. Grubbing reduced growing‐season NDVI by ~11% in mesic and moist habitats (Figure 3a,b), and by 6% in wet habitats (Figure 3c). The decrease in wet habitats was driven by a reduction (−11%) in peak summer NDVI only. Warming tended to increase early summer NDVI in mesic habitats (+5%; Figure 3a) and summer‐long NDVI in moist habitats (+4%; Figure 3b). In 2016 (Appendix S1: Figure S12), NDVI responses to grubbing were similar, whereas warming tended to increase growing‐season NDVI only in wet habitats (+3%).

### Relationships between CO_2_ fluxes and microenvironmental conditions

**FIGURE 4:**
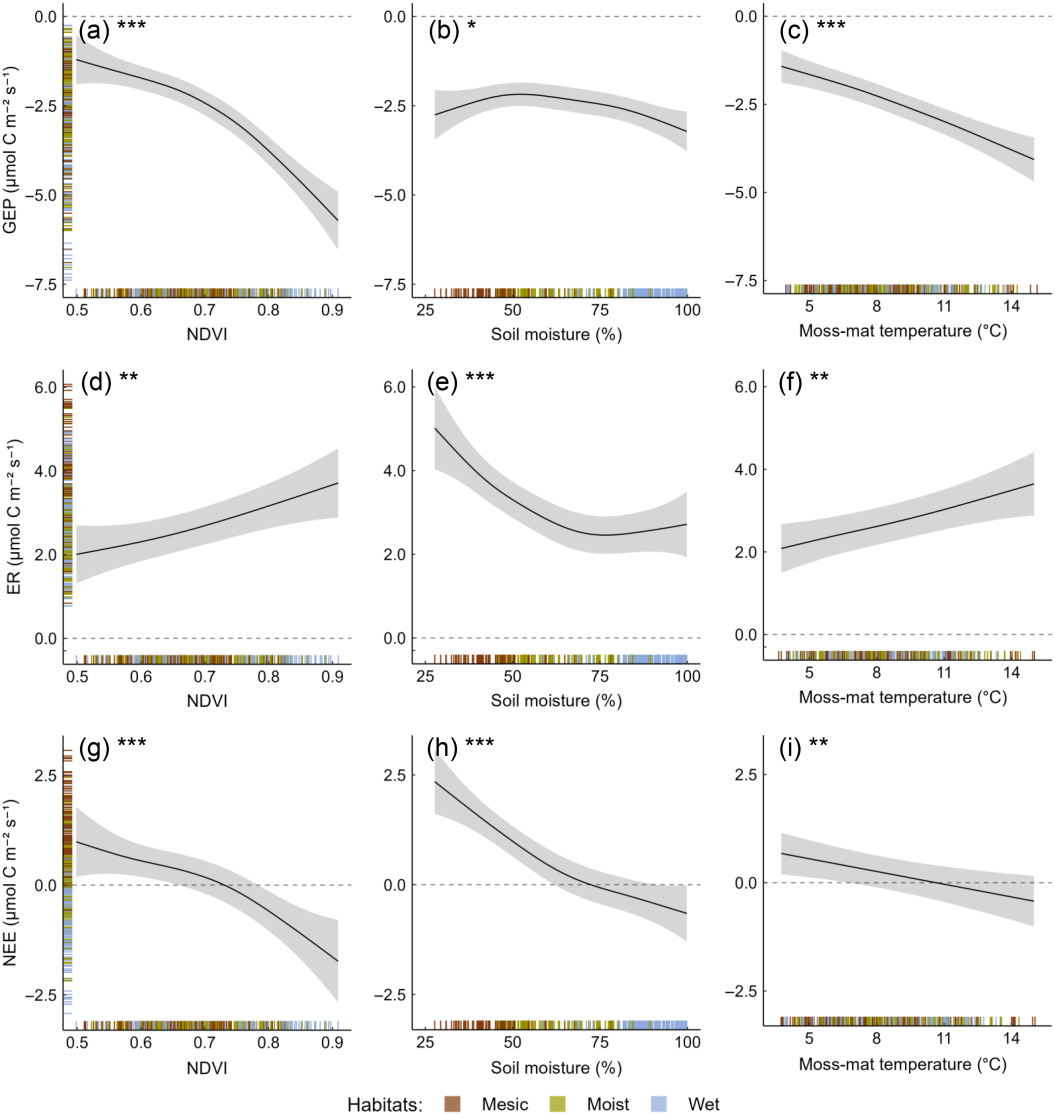
Across‐habitat relationships between ecosystem CO_2_ fluxes and both vegetation and abiotic variables in 2017. Regression lines ±95% CI for relations between (a–c) gross ecosystem productivity (GEP), (d–f) ecosystem respiration (ER), and (g–i) net ecosystem exchange (NEE) and the predictors (as additive smooth terms): normalized‐difference vegetation index (NDVI), soil moisture, and moss‐mat temperature (−2 cm). For each of the three models (GEP, ER, and NEE), the relationship with each predictor is shown at the average value of the other two predictors. Adjusted R^2^ for each model: GEP: 0.70; ER: 0.46; NEE: 0.44. Rugs on the *y*‐axis show predicted values, whereas rugs on the *x*‐axis show values of the predictors (all colored according to habitat). Significance of the smooth terms: *p < 0.05; **p < 0.01; and ***p < 0.001. ANOVA results are given in Appendix S1: Table S7. Positive and negative fluxes denote CO_2_ losses (the ecosystem acts as a C source) and CO_2_ gains (the ecosystem acts as a C sink), respectively. CO_2_‐flux relationships with air (+10 cm) and soil (−7 cm) temperatures were similar (not shown), plausibly because of the positive correlations among plot‐level temperatures (Appendix S1: Figure S14).

Across habitats, CO_2_ fluxes were related to both NDVI and abiotic variables. Both GEP and ER fluxes increased, although at a different rate, with increasing NDVI (Figure 4a,d), leading the ecosystem to switch from C source to C sink at values above ~0.7 (Figure 4g). ER fluxes, but not GEP fluxes, decreased with increasing soil moisture content, before leveling off at values exceeding ~70% (Figure 4b,e). These led to net ecosystem C release and sequestration below and above, respectively, this soil moisture threshold (Figure 4h). Because both GEP and ER fluxes increased with increasing temperature (Figure 4c,f), the NEE‐temperature relationship was weak (Figure 4i). Similar relationships held in 2016 (Appendix S1: Figure S13).

## DISCUSSION

We demonstrate three critical aspects of the sensitivity and magnitude of the CO_2_‐flux responses of Arctic ecosystems to environmental changes. First, both belowground spring herbivory by grubbing geese and elevated summer temperatures suppressed growing‐season net ecosystem CO_2_ uptake of this high‐Arctic ecosystem. Second, these two drivers elicited responses of similar magnitude, indicating that a disturbance occurring at the beginning of the growing season, such as grubbing, can have large impacts on tundra C balance, as does summer‐long warming. Finally, the ecosystem sensitivity to grubbing and warming varied across the three habitats, with drier habitats exhibiting stronger NEE responses to warming and wetter habitats exhibiting stronger NEE responses to herbivory. Combined, these results indicate a diminished ecosystem C sink strength capacity under increasing numbers of geese in a warmer Arctic. Our findings emphasize that predicting the future of this globally important C store amidst escalating global change relies on better understanding the spatial variability of tundra CO_2_ fluxes and their differential responses to key environmental‐change drivers.

We set out to examine whether grubbing and warming interact to affect tundra CO_2_ fluxes. We did not find interactive effects, which aligns with previous studies reporting additive effects of grubbing and warming (Ravolainen et al., 2020) on a range of ecosystem properties, such as moss and vascular plant biomass (Gornall et al., 2009) and soil and plant‐community nutrient levels (Petit Bon et al., 2021; Petit Bon, Bråthen, et al., 2023). In line with our prediction, we found these environmental‐change drivers to have additive antagonistic effects on both GEP and ER. This suggests that belowground spring herbivory can at least partly mediate tundra C cycling responses to elevated summer temperatures, as has been shown for aboveground grazing by geese (Leffler et al., 2019; Sjögersten et al., 2008) and ungulates (Cahoon et al., 2012; Ylänne et al., 2015). However, because the strength of these drivers differed across habitats, their combined effect was that of reducing net ecosystem C sequestration of both mesic and wet habitats. This indicates that neither grubbing nor warming dominates over the other, but their role as modifiers of NEE can vary within meters across the heterogeneous tundra landscape.

Grubbing caused the strongest decreases in summer‐long GEP, ER, and aboveground live biomass (i.e., NDVI) in moist (meadows) and mesic (heaths) habitats. These findings indicate that wet habitats (wetlands) are characterized by the lowest responsiveness in GEP and ER rates and the lowest loss of aboveground biomass to spring goose disturbance (cf. Petit Bon et al., 2021; Speed et al., 2010). Furthermore, they suggest that grubbing‐induced decreases in GEP and ER across habitats were largely driven by decreases in plant biomass (also note the tighter GEP‐NDVI than ER‐NDVI relationship [Figure 4a,d]; Van der Wal et al., 2007; Sjögersten et al., 2011; Petit Bon, Hansen, et al., 2023). Although the strongest grubbing‐induced changes in GEP and ER occurred in mesic and moist habitats, grubbing modified NEE by reducing GEP more than ER only in wet habitats. A plausible explanation is that the thicker moss layer in wetlands better protects their belowground component from disturbance (Petit Bon et al., 2021; Speed et al., 2010), and thus, grubbing had weaker effects on root and soil respiration in wet habitats. Our findings complement those from Sjögersten et al. (2008), who found aboveground goose grazing in this same Arctic ecosystem to also cause the largest decrease in C uptake in wetter habitats. As tundra wetlands have the strongest C sink strength (this study; Arndal et al., 2009; Oberbauer et al., 2007; Sjögersten et al., 2006) and experience the most extensive grubbing (Eischeid et al., 2023; Speed et al., 2009), this negative impact on C sequestration is likely to be disproportional to their occurrence. In the longer term, repeated grubbing may expose wetland soil to desiccating wind (Jefferies & Rockwell, 2002) and enhance soil temperatures (Gornall et al., 2009), thereby increasing ER and further reducing C uptake.

Grubbing promoted generally greater CO_2_‐flux changes in early‐to‐peak summer than in late summer in both wet and moist habitats, matching our expectation that its effects would be stronger soon after disturbance and diminish throughout the growing season. Yet, a more nuanced description is warranted. Grubbing decreased ER in moist habitats from early‐to‐late summer, suggesting that the recovery of ER in tundra meadows following spring disturbance might be slower than that of GEP. Both GEP and ER in mesic habitats were also reduced by grubbing from early‐to‐late summer, which aligns with drier habitats being characterized by slower recovery rates from disturbance than wetter habitats (Speed et al., 2010). This is relevant, as growing populations of grubbing geese in Svalbard and elsewhere (Fox & Madsen, 2017) are increasingly exploiting previously less‐used drier habitats (Pedersen, Tombre, et al., 2013), suggesting that substantial changes in tundra CO_2_ fluxes are already occurring. Also, the fact that grubbing reduced GEP and ER across all habitats indicates that the tundra in spring may be particularly vulnerable to herbivore disturbance. This is supported by findings from experimentally advanced goose grazing in coastal Alaska, which largely suppressed GEP by removing young plants before full leaf‐out, whereas typical and delayed grazing did not (Leffler et al., 2019). Combined, these findings highlight that CO_2_‐flux assessments that consider how environmental‐change impacts display across the growing season and among tundra habitats are crucial to capture accurately Arctic ecosystem C dynamics and their responses to perturbations.

Consistent with our expectations, warming promoted the strongest CO_2_‐flux responses in mesic habitats, where it caused a 7.5‐fold increase in summer‐long C release by stimulating ER more than GEP. Further, warming effects generally grew larger throughout the summer. The greater warming‐induced ER increase in mesic than in wetter habitats might stem from the control exerted by soil moisture on belowground ER (Sjögersten et al., 2006). In wetlands, higher temperatures increased GEP and aboveground plant respiration, but high soil moisture (average across the summer: ~90%) likely promoted soil anoxia, constraining belowground ER responses to warming. Conversely, soil moisture in mesic heaths (~40%) was unlikely to limit root or soil respiration, and thus, warming potentially stimulated both aboveground and belowground ER (Illeris et al., 2004; Oberbauer et al., 2007; but see Welker et al., 2004). Accordingly, because GEP did not substantially vary along the considered soil moisture gradient and because wet habitats had the greatest aboveground plant productivity (NDVI in this study; cf. Petit Bon, Bråthen, et al., 2023), the observed reduction in ER with increasing soil moisture must originate by its limiting effects on belowground processes. Optimum soil respiration conditions were also shown to be ~30%–45% soil moisture content in an alpine meadow (Knowles et al., 2015). In the longer term, permafrost thawing and associated thermokarst induced by warming (Schuur et al., 2022) may decrease soil moisture of some tundra habitats, thereby increasing ER (Rodenhizer et al., 2023) and thus C release.

Factors other than temperature per se and soil moisture, such as plant phenology and soil nutrient levels, may have contributed to the observed CO_2_‐flux responses to warming. For example, because in moist habitats higher temperatures tended to increase summer‐long NDVI and enhanced late summer ER, but not late summer GEP, the warming‐induced increase in late summer biomass must have been offset by a concomitant decrease in plant photosynthetic rates. Warming decreased plant‐community nitrogen and phosphorus concentrations most in moist habitats (−12%; Petit Bon, Bråthen, et al., 2023), potentially transposing to reduced photosynthetic rates (Kattge et al., 2009). In a Tibetan permafrost ecosystem, Li et al. (2017) also found that attenuated warming impacts on CO_2_ fluxes in late summer were associated with lower plant nutrient levels plausibly caused by accelerated plant senescence at higher temperatures. Concurrently, lower soil nitrogen in wet than mesic or moist habitats (Petit Bon et al., 2021; Petit Bon, Bråthen, et al., 2023) could partly explain the weaker wetland GEP responses to warming, as low nitrogen availability can constrain ecosystem productivity responses to temperature (Liu et al., 2022). A study encompassing 28 tundra OTC experiments showed that warming has stimulated summer ER by ~30% across the past 25 years, although large variations were detected among sites (Maes et al., 2024). Herein, we show that large variations in CO_2_‐flux responses to warming also occur among neighboring Arctic habitats and that the multitude of factors contributing to this variability challenges predictions of the tundra C budget.

Ecosystem CO_2_‐flux responses to grubbing and warming were generally consistent across the two years. This somewhat contrasts with differences in background process rates, with the warmer summer of 2016 being characterized by larger CO_2_ fluxes than the colder 2017. Wet habitats in 2016 were larger C sinks (even under treatments) than in 2017, owing to greater GEP but similar ER. In a Canadian high‐Arctic ecosystem, interannual variations in NEE were also attributed to higher variability in GEP (and hence NDVI) than ER (Braybrook et al., 2021). Therefore, though the often‐large between‐year variability in abiotic and biotic conditions in the Arctic can promote large between‐year variability in CO_2_ fluxes, grubbing and warming appear to alter consistently C exchange rates in this Svalbard ecosystem.

From this same experiment, we have demonstrated that grubbing reduces nitrogen and phosphorus pools in plant communities, although it generally increases nutrient concentrations, whereas warming has the opposite effects (Petit Bon et al., 2021; Petit Bon, Bråthen, et al., 2023). Here, we show that these rapid nutrient‐level changes are accompanied by decreases in summer‐long net ecosystem CO_2_ uptake. Moreover, lower plant biomass (cf. Petit Bon, Bråthen, et al., 2023) and lower soil C stocks (Van der Wal et al., 2007) with grubbing suggest the potential for longer term negative consequences for ecosystem C pool (cf. Petit Bon, Hansen, et al., 2023). These findings indicate a significant decrease in the capacity of Svalbard ecosystems to store C, as the three habitats studied here account for ~10% (>2500 km^2^) of the glacier‐free area (Johansen et al., 2012). Additionally, tundra heaths, meadows, and wetlands cover large parts of the Middle Arctic tundra (Walker et al., 2005), highlighting the potentially far‐reaching implications of our results. Consequently, the expansion of Arctic‐breeding goose populations—particularly species of the genera *Anser* and *Chen*, which feed by grubbing at the start of the growing season—is likely contributing to the substantial alterations in tundra C and nutrient cycling driven by climate warming.

## AUTHOR CONTRIBUTIONS

Matteo Petit Bon, Ingibjörg S. Jónsdóttir, and Kari Anne Bråthen designed the experiment. Matteo Petit Bon and Hanna Lee designed methodology. Matteo Petit Bon set up and ran the experiment and collected and processed the data. Matteo Petit Bon and Karen H. Beard explored the data and discussed the main patterns. Matteo Petit Bon analyzed the data and wrote the paper, to which all authors contributed critically.

## CONFLICT OF INTEREST STATEMENT

The authors declare no conflicts of interest.

## Supporting information


Appendix S1.

## Data Availability

Data (Petit Bon et al., 2024) are available in UiT The Arctic University of Norway's DataverseNO repository at https://doi.org/10.18710/HJN3LV.
