# Supplementary material for: Goose grubbing and warming suppress summer net ecosystem CO2 uptake differentially across high‐Arctic tundra habitats
Source: Ecology. 2024 Dec 9;106(1):e4498. doi: 10.1002/ecy.4498 (PMC11739665; doi:10.1002/ecy.4498)
Supplement: Supplementary file 1 — Appendix S1. [file ECY-106-e4498-s001.pdf]

Journal: Ecology

APPENDIX S1

Supplementary tables, figures, results, and methods accompanying the article

‘**Goose grubbing and warming suppress summer net ecosystem CO_2 uptake differentially across high-Arctic tundra habitats**’

Matteo Petit Bon, Karen H. Beard, Kari Anne Bråthen, Hanna Lee, Ingibjörg S. Jónsdóttir

Index:

1) **Supplementary Tables** (page 3):
    a. Table S1. Overview of the final dataset used for analyses.
    b. Tables S2-S13. ANOVA summaries and parameter estimates from statistical modelling.
2) **Supplementary Figures** (page 25):
    a. Figure S1. *In-situ* environmental characteristics of the study area.
    b. Figure S2. Overview of the three habitats and of the simulated and natural spring goose grubbing.
    c. Figures S3-S5. Plot-level temperatures in the four treatment combinations.
    d. Figure S6. Relationships between live biomass and NDVI.
    e. Figure S7. Comparison of experimental and natural grubbing effects.
    f. Figures S8-S10. Relationships between CO_2-fluxes and environmental conditions and their variation among treatment and habitat measures.
    g. Figures S11-S13. Main results for the first year of the experiment (2016).
    h. Figure S14. Relationships between plot-level temperatures during CO_2-flux measurements.
3) **Section S1** (page 43): Details on the non-metric multidimensional scaling shown in Figure 1.
4) **Section S2** (page 46): Additional details on data analyses.
5) References (page 52).

Supplementary Tables

TABLE S1. Overview of the final dataset used for analyses. Measurements of gross ecosystem productivity (GEP), ecosystem respiration (ER), net ecosystem exchange (NEE), and normalized-difference vegetation index (NDVI) sorted according to habitat (mesic, moist, and wet habitats), summer period (early, peak, and late), and treatments (un-grubbed/ambient, grubbed/ambient, un-grubbed/warmed, and grubbed/warmed plots), and further separated for year **(a)** 2016 and **(b)** 2017. The number of measurements is further summarized across summer periods (*All summer*), separately for each treatment, and across treatments (*All treatments*), separately for each summer period. The number in bold gives the number of observations for each 'CO_2-flux component (or NDVI) × habitat' combination; note that this number also represents the number of observations for each of our statistical models (see Table S3-S6 for year 2017 and Table S9-S12 for year 2016, and *Material and Methods: Statistical analyses* in the main text for details). Out of the collected 3619 CO_2-flux data points and 960 NDVI data points, 3572 (CO_2-fluxes) and 959 (NDVI) data points (overview presented in this table) were used for analysis (see Appendix S1: Section S2 for details about data exploration).

Table S1.

	Variable	Treatment	Mesic habitats				Moist habitats				Wet habitats			
			Early	Mid	Late	*All summer*	Early	Mid	Late	*All summer*	Early	Mid	Late	*All summer*
(a) Year 2016	Gross ecosystem productivity (GEP)	Ungrubbed/Ambient	13	14	14	*41*	14	14	14	*42*	12	12	12	*36*
		Grubbed/Ambient	12	14	14	*40*	14	14	14	*42*	12	12	11	*35*
		Ungrubbed/Warming	14	14	14	*42*	14	14	14	*42*	12	12	12	*36*
		Grubbed/Warming	14	14	14	*42*	13	13	14	*40*	12	12	11	*35*
		All treatments	*53*	*56*	*56*	**165**	*55*	*55*	*56*	**166**	*48*	*48*	*46*	**142**
	Ecosystem respiration (ER)	Ungrubbed/Ambient	14	14	14	*42*	14	13	14	*41*	12	12	12	*36*
		Grubbed/Ambient	12	14	14	*40*	14	13	14	*41*	12	12	12	*36*
		Ungrubbed/Warming	14	14	14	*42*	14	13	13	*40*	12	12	12	*36*
		Grubbed/Warming	14	13	14	*41*	13	14	14	*41*	12	12	12	*36*
		All treatments	*54*	*55*	*56*	**165**	*55*	*53*	*55*	**163**	*48*	*48*	*48*	**144**
	Net ecosystem exchange (NEE)	Ungrubbed/Ambient	13	14	14	*41*	14	13	14	*41*	12	12	12	*36*
		Grubbed/Ambient	14	14	14	*42*	14	13	14	*41*	12	12	11	*35*
		Ungrubbed/Warming	14	14	14	*42*	14	13	13	*40*	12	12	12	*36*
		Grubbed/Warming	14	14	14	*42*	14	13	14	*41*	12	12	11	*35*
		All treatments	*55*	*56*	*56*	**167**	*56*	*52*	*55*	**163**	*48*	*48*	*46*	**142**
	Normalized-difference vegetation index (NDVI)	Ungrubbed/Ambient	14	14	14	*42*	14	14	14	*42*	12	12	12	*36*
		Grubbed/Ambient	14	14	14	*42*	14	14	14	*42*	12	12	12	*36*
		Ungrubbed/Warming	14	14	14	*42*	14	14	14	*42*	12	12	12	*36*
		Grubbed/Warming	14	14	14	*42*	14	14	14	*42*	11	12	12	*35*
		All treatments	*56*	*56*	*56*	**168**	*56*	*56*	*56*	**168**	*47*	*48*	*48*	**143**
(b) Year 2017	Gross ecosystem productivity (GEP)	Ungrubbed/Ambient	13	13	13	*39*	14	14	14	*42*	12	10	12	*34*
		Grubbed/Ambient	14	13	13	*40*	14	13	13	*40*	12	12	12	*36*
		Ungrubbed/Warming	14	14	14	*42*	13	14	13	*40*	12	12	12	*36*
		Grubbed/Warming	14	13	14	*41*	14	14	14	*42*	12	12	12	*36*
		All treatments	*55*	*53*	*54*	**162**	*55*	*55*	*54*	**164**	*48*	*46*	*48*	**142**
	Ecosystem respiration (ER)	Ungrubbed/Ambient	14	14	14	*42*	14	14	14	*42*	12	12	12	*36*
		Grubbed/Ambient	14	14	13	*41*	13	13	13	*39*	12	12	12	*36*
		Ungrubbed/Warming	14	13	14	*41*	14	14	14	*42*	12	12	12	*36*
		Grubbed/Warming	14	13	13	*40*	14	14	14	*42*	12	12	12	*36*
		All treatments	*56*	*54*	*54*	**164**	*55*	*55*	*55*	**165**	*48*	*48*	*48*	**144**
	Net ecosystem exchange (NEE)	Ungrubbed/Ambient	13	13	13	*39*	14	14	14	*42*	12	10	12	*34*
		Grubbed/Ambient	14	13	14	*41*	13	12	14	*39*	12	12	12	*36*
		Ungrubbed/Warming	14	13	14	*41*	13	14	12	*39*	12	12	12	*36*
		Grubbed/Warming	14	12	14	*40*	14	14	14	*42*	12	12	12	*36*
		All treatments	*55*	*51*	*55*	**161**	*54*	*54*	*54*	**162**	*48*	*46*	*48*	**142**
	Normalized-difference vegetation index (NDVI)	Ungrubbed/Ambient	14	14	14	*42*	14	14	14	*42*	12	12	12	*36*
		Grubbed/Ambient	14	14	14	*42*	14	14	14	*42*	12	12	12	*36*
		Ungrubbed/Warming	14	14	14	*42*	14	14	14	*42*	12	12	12	*36*
		Grubbed/Warming	14	14	14	*42*	14	14	14	*42*	12	12	12	*36*
		All treatments	*56*	*56*	*56*	**168**	*56*	*56*	*56*	**168**	*48*	*48*	*48*	**144**

TABLE S2. The effects of spring goose grubbing, summer warming, summer season, and their interactions on CO_2-fluxes and normalized-difference vegetation index in 2017. F values and associated degrees of freedom (df; presented in the order of nominator and denominator), as well as the significance level (*P-value*) for the predictors, were determined by using analysis of variance on the linear mixed-effects models (see *Material and Methods: Statistical analyses* in the main text for details and Table S3-S6 for parameter estimates of these models). Significant (P < 0.05) and marginally significant (P < 0.1) main and interactive effects are shown in bold and italic font, respectively.

Model	Predictors	Mesic habitats		Moist habitats		Wet habitats	
		$F_{(df)}$	*P-value*	$F_{(df)}$	*P-value*	$F_{(df)}$	*P-value*
Gross ecosystem productivity (GEP)	Grubbing	**13.3$_{(1,19)}$**	**0.0017**	**37.2$_{(1,19)}$**	**7.8E-06**	**14.6$_{(1,16)}$**	**0.0015**
	Warming	**4.6$_{(1,19)}$**	**0.0449**	**15.0$_{(1,19)}$**	**0.0011**	*4.2 $_{(1,16)}$*	*0.0578*
	Summer season	**6.7$_{(2,129)}$**	**0.0017**	**40.8$_{(2,130)}$**	**1.8E-14**	**44.7$_{(2,112)}$**	**5.4E-15**
	Grubbing × Summer season	1.6$_{(2,129)}$	0.2067	**7.2$_{(2,130)}$**	**0.0011**	**9.6$_{(2,112)}$**	**0.0001**
	Warming × Summer season	1.2$_{(2,129)}$	0.3003	**5.3$_{(2,130)}$**	**0.0061**	2.3$_{(2,112)}$	0.1007
Ecosystem respiration (ER)	Grubbing	**15.9$_{(1,52)}$**	**0.0002**	**11.8$_{(1,25)}$**	**0.0020**	**5.9$_{(1,16)}$**	**0.0272**
	Warming	**45.4$_{(1,52)}$**	**1.3E-08**	**6.9$_{(1,25)}$**	**0.0142**	**7.6$_{(1,16)}$**	**0.0142**
	Summer season	*2.9 $_{(2,102)}$*	*0.0610*	**10.7$_{(2,103)}$**	**0.0001**	**16.8$_{(2,114)}$**	**4.1E-07**
	Grubbing × Summer season	1.4$_{(2,102)}$	0.2583	0.6$_{(2,103)}$	0.5686	**3.2$_{(2,114)}$**	**0.0460**
	Warming × Summer season	0.9$_{(2,,102)}$	0.4239	0.7$_{(2,,103)}$	0.4870	0.6$_{(2,,114)}$	0.5338
Net ecosystem exchange (NEE)	Grubbing	0.2$_{(1,46)}$	0.6877	0.1$_{(1,24)}$	0.7180	**7.9$_{(1,21)}$**	**0.0106**
	Warming	**22.1$_{(1,46)}$**	**2.4E-05**	0.5$_{(1,24)}$	0.4889	<0.1$_{(1,21)}$	0.8798
	Summer season	**4.3$_{(2,95)}$**	**0.0168**	**11.4$_{(2,126)}$**	**2.9E-05**	**20.8$_{(2,112)}$**	**2.1E-08**
	Grubbing × Summer season	0.9$_{(2,95)}$	0.4010	**6.7$_{(2,126)}$**	**0.0017**	**6.0$_{(2,112)}$**	**0.0033**
	Warming × Summer season	**3.8$_{(2,95)}$**	**0.0266**	**6.3$_{(2,126)}$**	**0.0025**	1.2$_{(2,112)}$	0.2932
Normalized-difference vegetation index (NDVI)	Grubbing	**36.7$_{(1,47)}$**	**2.2E-07**	**23.5$_{(1,25)}$**	**0.0001**	**8.6$_{(1,16)}$**	**0.0097**
	Warming	2.4$_{(1,47)}$	0.1249	*3.2 $_{(1,25)}$*	*0.0872*	1.7$_{(1,16)}$	0.2051
	Summer season	**76.1$_{(2,106)}$**	**2.2E-16**	**70.2$_{(2,134)}$**	**2.2E-16**	**82.9$_{(2,114)}$**	**2.2E-16**
	Grubbing × Summer season	**3.4$_{(2,106)}$**	**0.0382**	0.4$_{(2,134)}$	0.6580	**5.0$_{(2,114)}$**	**0.0080**
	Warming × Summer season	*2.8 $_{(2,,106)}$*	*0.0654*	1.3$_{(2,134)}$	0.2745	0.2$_{(2,114)}$	0.8121

TABLE S3. Parameter estimates from the linear mixed-effects models for the effects of spring goose grubbing and summer warming on ***gross ecosystem productivity (GEP)*** throughout the summer of 2017. Parameter estimates of fixed-effects (*Est.*) and their 95% confidence interval (CI – lower and upper bounds) for models on GEP (µmol CO_2 m^{-2} s^{-1}) of mesic, moist, and wet habitats. Predictors are 'spring goose grubbing' (two-level factor: un-grubbed and grubbed plots), 'summer warming' (two-level factor: ambient and warmed plots), and 'summer season' (three-level factor: early, peak, and late summer). To allow a complete overview for the reader, intercepts (i.e. reference levels – RL) are calculated for (i) all main contrasts (i.e. main effects) and (ii) all two-way contrasts (i.e. two-way interacting effects) retained in the final models (see main text for details). As the two-way interaction 'spring goose grubbing × summer warming' and the three-way interaction 'spring goose grubbing × summer warming × summer season' were not retained in the final models (see main text for details), we present (i) the main effects of 'spring goose grubbing' and 'summer warming' and (ii) their two-way interacting effects with 'summer season' by using 'ambient' and 'non-grubbing' plots, respectively, as reference levels. However, note that estimates and their 95% confidence intervals would be the same if 'warmed' and 'grubbed' plots, respectively, were used instead. Estimates in bold indicate that their 95% CI does not include zero (i.e. statistically significant effects), while estimates in italic indicate that their 90% CI does not include zero (i.e. marginally significant effects). Random-effects, which are presented as standard deviations, as well as the number of observations for each model, are reported at the bottom of the table.

Table S3.

Fixed-effects	Contrast	Baseline combination for the contrast of interest	Mesic habitats				Moist habitats				Wet habitats			
			RL	Est.	Low CI	Up CI	RL	Est.	Low CI	Up CI	RL	Est.	Low CI	Up CI
(i) Main effects	Grubbing *vs* Non-grubbing	Ambient/Early	-2.27	**0.97**	**0.08**	**1.86**	-2.42	**1.55**	**0.91**	**2.19**	-2.66	**1.51**	**0.45**	**2.57**
		Ambient/Peak	-3.34	**1.71**	**0.81**	**2.61**	-3.58	**2.06**	**1.42**	**2.70**	-5.71	**2.86**	**1.78**	**3.93**
		Ambient/Late	-2.83	**1.38**	**0.48**	**2.27**	-1.70	*0.60*	*-0.05*	*1.24*	-3.25	0.50	-0.56	1.56
	Warming *vs* Ambient	Non-grubbing/Early	-2.27	**-0.99**	**-1.88**	**-0.09**	-2.42	**-1.12**	**-1.76**	**-0.48**	-2.66	**-1.11**	**-2.18**	**-0.05**
		Non-grubbing/Peak	-3.34	**-0.98**	**-1.88**	**-0.08**	-3.58	**-1.38**	**-2.02**	**-0.74**	-5.71	**-1.28**	**-2.36**	**-0.21**
		Non-grubbing/Late	-2.83	-0.42	-1.32	0.47	-1.70	-0.17	-0.81	0.48	-3.25	-0.21	-1.27	0.86
	Summer season: Peak vs Early	Non-grubbing/Ambient	-2.27	**-1.07**	**-1.78**	**-0.35**	-2.42	**-1.16**	**-1.82**	**-0.49**	-2.66	**-3.05**	**-4.00**	**-2.10**
		Grubbing/Ambient	-1.30	-0.33	-1.04	0.38	-0.87	*-0.65*	*-1.32*	*0.02*	-1.16	**-1.70**	**-2.62**	**-0.78**
		Non-grubbing/Warming	-3.26	**-1.06**	**-1.76**	**-0.36**	-3.54	**-1.42**	**-2.09**	**-0.75**	-3.78	**-3.22**	**-4.14**	**-2.30**
		Grubbing/Warming	-2.28	-0.32	-1.03	0.39	-1.99	**-0.91**	**-1.57**	**-0.25**	-2.27	**-1.87**	**-2.78**	**-0.95**
	Summer season: Late vs Peak	Non-grubbing/Ambient	-3.34	0.51	-0.22	1.23	-3.58	**1.88**	**1.22**	**2.54**	-5.71	**2.46**	**1.51**	**3.41**
		Grubbing/Ambient	-1.63	0.17	-0.55	0.89	-1.52	0.42	-0.26	1.10	-2.86	0.11	-0.81	1.02
		Non-grubbing/Warming	-4.32	**1.06**	**0.36**	**1.76**	-4.96	**3.09**	**2.42**	**3.76**	-7.00	**3.54**	**2.62**	**4.46**
		Grubbing/Warming	-2.61	**0.73**	**0.02**	**1.44**	-2.90	**1.63**	**0.97**	**2.30**	-4.14	**1.18**	**0.27**	**2.10**
	Summer season: Late *vs* Early	Non-grubbing/Ambient	-2.27	-0.56	-1.28	0.16	-2.42	**0.72**	**0.06**	**1.39**	-2.66	-0.59	-1.50	0.32
		Grubbing/Ambient	-1.30	-0.16	-0.87	0.55	-0.87	-0.23	-0.90	0.44	-1.16	**-1.59**	**-2.51**	**-0.68**
		Non-grubbing/Warming	-3.26	0.002	-0.70	0.70	-3.54	**1.67**	**0.99**	**2.35**	-3.78	0.32	-0.59	1.23
		Grubbing/Warming	-2.28	0.40	-0.29	1.10	-1.99	**0.72**	**0.06**	**1.38**	-2.27	-0.69	-1.60	0.23
(ii) Two-way interacting effects	Grubbing vs Non-grubbing Peak vs Early	Ambient	-2.27	*0.74*	*-0.08*	*1.56*	-2.42	0.51	-0.26	1.28	-2.66	**1.35**	**0.29**	**2.42**
	Grubbing vs Non-grubbing Late vs Peak	Ambient	-3.34	-0.34	-1.16	0.49	-3.58	**-1.46**	**-2.23**	**-0.68**	-5.71	**-2.36**	**-3.43**	**-1.29**
	Grubbing *vs* Non-grubbing	Ambient	-2.27	0.40	-0.41	1.22	-2.42	**-0.95**	**-1.73**	**-0.18**	-2.66	*-1.01*	*-2.06*	*0.05*
	Warming vs Ambient Peak vs Early	Non-grubbing	-2.27	0.004	-0.81	0.82	-2.42	-0.26	-1.03	0.51	-2.66	-0.17	-1.24	0.90
	Warming vs Ambient Late vs Peak	Non-grubbing	-3.34	0.56	-0.27	1.38	-3.58	**1.21**	**0.44**	**1.99**	-5.71	**1.08**	**0.01**	**2.14**
	Warming *vs* Ambient Late *vs* Early	Non-grubbing	-2.27	0.56	-0.25	1.38	-2.42	**0.95**	**0.18**	**1.73**	-2.66	*0.91*	*-0.15*	*1.96*

Random-effects	Mesic Std.Dev.	Mesic Low CI	Mesic Up CI	Moist Std.Dev.	Moist Low CI	Moist Up CI	Wet Std.Dev.	Wet Low CI	Wet Up CI
Block	0.72	0.17	1.41	0.42	0.09	0.85	1.17	0.33	2.16
Plot_ID	0.87	0.54	1.26	0.44	0.16	0.78	0.89	0.49	1.36
Residual	1.07	0.95	1.20	1.02	0.90	1.14	1.30	1.13	1.48
Observations	162			164			142		

TABLE S4. Parameter estimates from the linear mixed-effects models for the effects of spring goose grubbing and summer warming on ***ecosystem respiration (ER)*** throughout the summer of 2017. Parameter estimates of fixed-effects (*Est.*) and their 95% confidence interval (CI – lower and upper bounds) for models on ER (μmol CO_2 m^{-2} s^{-1}) of mesic, moist, and wet habitats. Predictors are 'spring goose grubbing' (two-level factor: un-grubbed and grubbed plots), 'summer warming' (two-level factor: ambient and warmed plots), and 'summer season' (three-level factor: early, peak, and late summer). To allow a complete overview for the reader, intercepts (i.e. reference levels – RL) are calculated for (i) all main contrasts (i.e. main effects) and (ii) all two-way contrasts (i.e. two-way interacting effects) retained in the final models (see main text for details). As the two-way interaction 'spring goose grubbing × summer warming' and the three-way interaction 'spring goose grubbing × summer warming × summer season' were not retained in the final models (see main text for details), we present (i) the main effects of 'spring goose grubbing' and 'summer warming' and (ii) their two-way interacting effects with 'summer season' by using 'ambient' and 'non-grubbing' plots, respectively, as reference levels. However, note that estimates and their 95% confidence intervals would be the same if 'warmed' and 'grubbed' plots, respectively, were used instead. Estimates in bold indicate that their 95% CI does not include zero (i.e. statistically significant effects), while estimates in italic indicate that their 90% CI does not include zero (i.e. marginally significant effects). Random-effects, which are presented as standard deviations, as well as the number of observations for each model, are reported at the bottom of the table.

Table S4.

Fixed-effects	Contrast	Baseline combination for the contrast of interest	Mesic habitats				Moist habitats				Wet habitats			
			RL	Est.	Low CI	Up CI	RL	Est.	Low CI	Up CI	RL	Est.	Low CI	Up CI
(i) Main effects	Grubbing *vs* Non-grubbing	Ambient/Early	2.94	**-1.26**	**-2.26**	**-0.26**	3.08	**-1.75**	**-2.74**	**-0.75**	2.23	-0.48	-1.26	0.30
		Ambient/Peak	3.71	**-1.99**	**-3.01**	**-0.97**	3.16	**-1.38**	**-2.37**	**-0.38**	3.40	**-1.30**	**-2.07**	**-0.52**
		Ambient/Late	2.87	*-0.92*	*-1.95*	*0.11*	2.29	**-1.40**	**-2.40**	**-0.40**	2.35	-0.57	-1.35	0.21
	Warming *vs* Ambient	Non-grubbing/Early	2.94	**1.86**	**0.86**	**2.86**	3.08	*0.89*	*-0.11*	*1.88*	2.23	**0.87**	**0.09**	**1.64**
		Non-grubbing/Peak	3.71	**2.60**	**1.58**	**3.62**	3.16	**1.31**	**0.32**	**2.31**	3.40	**1.10**	**0.32**	**1.87**
		Non-grubbing/Late	2.87	**2.59**	**1.57**	**3.62**	2.29	**1.27**	**0.27**	**2.27**	2.35	*0.70*	*-0.08*	*1.48*
	Summer season: Peak vs Early	Non-grubbing/Ambient	2.94	0.77	-0.34	1.88	3.08	0.08	-0.58	0.74	2.23	**1.18**	**0.57**	**1.79**
		Grubbing/Ambient	1.68	0.04	-1.07	1.14	1.33	0.45	-0.23	1.13	1.74	0.37	-0.24	0.98
		Non-grubbing/Warming	4.80	**1.51**	**0.39**	**2.64**	3.97	0.50	-0.16	1.16	3.09	**1.41**	**0.80**	**2.02**
		Grubbing/Warming	3.54	0.78	-0.35	1.90	2.22	**0.87**	**0.21**	**1.53**	2.61	*0.60*	*-0.01*	*1.21*
	Summer season: Late vs Peak	Non-grubbing/Ambient	3.71	-0.84	-1.95	0.28	3.16	**-0.87**	**-1.53**	**-0.21**	3.40	**-1.05**	**-1.66**	**-0.45**
		Grubbing/Ambient	1.72	0.23	-0.90	1.36	1.78	**-0.89**	**-1.57**	**-0.21**	2.11	-0.33	-0.94	0.28
		Non-grubbing/Warming	6.31	-0.84	-1.99	0.30	4.47	**-0.91**	**-1.59**	**-0.24**	4.50	**-1.45**	**-2.06**	**-0.84**
		Grubbing/Warming	4.32	0.22	-0.92	1.36	3.09	**-0.94**	**-1.60**	**-0.27**	3.20	**-0.72**	**-1.33**	**-0.12**
	Summer season: Late *vs* Early	Non-grubbing/Ambient	2.94	-0.07	-1.18	1.04	3.08	**-0.79**	**-1.45**	**-0.13**	2.23	0.13	-0.48	0.73
		Grubbing/Ambient	1.68	0.26	-0.86	1.39	1.33	-0.45	-1.13	0.23	1.74	0.04	-0.57	0.65
		Non-grubbing/Warming	4.80	0.67	-0.46	1.80	3.97	-0.41	-1.08	0.27	3.09	-0.04	-0.65	0.57
		Grubbing/Warming	3.54	*1.00*	*-0.13*	*2.13*	2.22	-0.06	-0.73	0.60	2.61	-0.13	-0.73	0.48
(ii) Two-way interacting effects	Grubbing vs Non-grubbing Peak vs Early	Ambient	2.94	-0.73	-2.02	0.56	3.08	0.37	-0.40	1.14	2.23	**-0.81**	**-1.51**	**-0.11**
	Grubbing vs Non-grubbing Late vs Peak	Ambient	3.71	1.07	-0.24	2.37	3.16	-0.03	-0.80	0.75	3.40	**0.72**	**0.02**	**1.43**
	Grubbing *vs* Non-grubbing	Ambient	2.94	0.33	-0.96	1.63	3.08	0.34	-0.43	1.12	2.23	-0.09	-0.79	0.61
	Warming vs Ambient Peak vs Early	Non-grubbing	2.94	0.74	-0.55	2.03	3.08	0.42	-0.34	1.19	2.23	0.23	-0.47	0.93
	Warming vs Ambient Late vs Peak	Non-grubbing	3.71	-0.01	-1.32	1.30	3.16	-0.04	-0.82	0.73	3.40	-0.40	-1.10	0.31
	Warming *vs* Ambient Late *vs* Early	Non-grubbing	2.94	0.74	-0.56	2.03	3.08	0.38	-0.39	1.16	2.23	-0.17	-0.87	0.54
Random-effects				Std.Dev.	Low CI	Up CI		Std.Dev.	Low CI	Up CI		Std.Dev.	Low CI	Up CI
Block												0.77	0.21	1.46
Plot_ID								0.99	0.62	1.40		0.71	0.42	1.05
Subplot_ID				0.84	0.39	1.35		0.60	0.28	0.97				
Residual				1.70	1.47	1.93		1.02	0.88	1.16		0.87	0.75	0.98
Observations				163				164				144		

TABLE S5. Parameter estimates from the linear mixed-effects models for the effects of spring goose grubbing and summer warming on ***net ecosystem exchange (NEE)*** throughout the summer of 2017. Parameter estimates of fixed-effects (*Est.*) and their 95% confidence interval (CI – lower and upper bounds) for models on NEE (μmol CO_2 m^{-2} s^{-1}) of mesic, moist, and wet habitats. Predictors are 'spring goose grubbing' (two-level factor: un-grubbed and grubbed plots), 'summer warming' (two-level factor: ambient and warmed plots), and 'summer season' (three-level factor: early, peak, and late summer). To allow a complete overview for the reader, intercepts (i.e. reference levels – RL) are calculated for (i) all main contrasts (i.e. main effects) and (ii) all two-way contrasts (i.e. two-way interacting effects) retained in the final models (see main text for details). As the two-way interaction 'spring goose grubbing × summer warming' and the three-way interaction 'spring goose grubbing × summer warming × summer season' were not retained in the final models (see main text for details), we present (i) the main effects of 'spring goose grubbing' and 'summer warming' and (ii) their two-way interacting effects with 'summer season' by using 'ambient' and 'non-grubbing' plots, respectively, as reference levels. However, note that estimates and their 95% confidence intervals would be the same if 'warmed' and 'grubbed' plots, respectively, were used instead. Estimates in bold indicate that their 95% CI does not include zero (i.e. statistically significant effects), while estimates in italic indicate that their 90% CI does not include zero (i.e. marginally significant effects). Random-effects, which are presented as standard deviations, as well as the number of observations for each model, are reported at the bottom of the table. Positive and negative fluxes denote CO_2 losses (the ecosystem acts as a C source) and CO_2 gains (the ecosystem acts as a C sink), respectively.

Table S5.

Fixed-effects	Contrast	Baseline combination for the contrast of interest	Mesic habitats				Moist habitats				Wet habitats			
			RL	Est.	Low CI	Up CI	RL	Est.	Low CI	Up CI	RL	Est.	Low CI	Up CI
(i) Main effects	Grubbing *vs* Non-grubbing	Ambient/Early	0.70	-0.34	-1.59	0.91	0.58	0.03	-0.65	0.71	-0.44	**1.02**	**0.18**	**1.86**
		Ambient/Peak	0.02	0.07	-1.22	1.36	-0.74	**0.80**	**0.12**	**1.48**	-2.44	**1.65**	**0.79**	**2.51**
		Ambient/Late	0.05	0.77	-0.47	2.02	0.46	-0.54	-1.22	0.15	-0.90	-0.07	-0.91	0.77
	Warming *vs* Ambient	Non-grubbing/Early	0.70	0.91	-0.33	2.16	0.58	-0.46	-1.14	0.22	-0.44	-0.25	-1.09	0.59
		Non-grubbing/Peak	0.02	**1.76**	**0.47**	**3.05**	-0.74	0.19	-0.49	0.88	-2.44	-0.10	-0.96	0.76
		Non-grubbing/Late	0.05	**3.17**	**1.92**	**4.41**	0.46	**0.83**	**0.15**	**1.52**	-0.90	0.49	-0.35	1.33
	Summer season: Peak vs Early	Non-grubbing/Ambient	0.70	-0.69	-2.15	0.77	0.58	**-1.32**	**-1.94**	**-0.69**	-0.44	**-2.00**	**-2.89**	**-1.12**
		Grubbing/Ambient	0.36	-0.28	-1.72	1.17	0.61	*-0.55*	*-1.19*	*0.09*	0.59	**-1.38**	**-2.23**	**-0.52**
		Non-grubbing/Warming	1.62	0.16	-1.29	1.60	0.12	**-0.66**	**-1.29**	**-0.04**	-0.69	**-1.86**	**-2.71**	**-1.00**
		Grubbing/Warming	1.28	0.57	-0.90	2.04	0.15	0.10	-0.51	0.72	0.34	**-1.23**	**-2.09**	**-0.37**
	Summer season: Late vs Peak	Non-grubbing/Ambient	0.02	0.03	-1.44	1.50	-0.74	**1.20**	**0.57**	**1.82**	-2.44	**1.54**	**0.66**	**2.43**
		Grubbing/Ambient	0.09	0.73	-0.71	2.18	0.06	-0.14	-0.78	0.49	-0.79	-0.18	-1.04	0.68
		Non-grubbing/Warming	1.78	*1.44*	*-0.01*	*2.89*	-0.55	**1.84**	**1.19**	**2.48**	-2.54	**2.14**	**1.28**	**2.99**
		Grubbing/Warming	1.85	**2.14**	**0.68**	**3.61**	0.25	0.50	-0.12	1.12	-0.89	0.41	-0.44	1.27
	Summer season: Late *vs* Early	Non-grubbing/Ambient	0.70	-0.66	-2.11	0.80	0.58	-0.12	-0.74	0.49	-0.44	-0.46	-1.32	0.39
		Grubbing/Ambient	0.36	0.46	-0.96	1.87	0.61	**-0.69**	**-1.32**	**-0.07**	0.59	**-1.56**	**-2.41**	**-0.70**
		Non-grubbing/Warming	1.62	**1.60**	**0.18**	**3.01**	0.12	**1.17**	**0.53**	**1.82**	-0.69	0.28	-0.57	1.13
		Grubbing/Warming	1.28	**2.71**	**1.30**	**4.13**	0.15	*0.60*	*-0.01*	*1.22*	0.34	*-0.81*	*-1.67*	*0.04*
(ii) Two-way interacting effects	Grubbing vs Non-grubbing Peak vs Early	Ambient	0.70	0.41	-1.27	2.09	0.58	**0.77**	**0.05**	**1.49**	-0.44	0.63	-0.37	1.63
	Grubbing vs Non-grubbing Late vs Peak	Ambient	0.02	0.71	-0.98	2.39	-0.74	**-1.34**	**-2.07**	**-0.61**	-2.44	**-1.72**	**-2.72**	**-0.72**
	Grubbing *vs* Non-grubbing	Ambient	0.70	1.12	-0.53	2.76	0.58	-0.57	-1.29	0.15	-0.44	**-1.09**	**-2.08**	**-0.11**
	Warming vs Ambient Peak vs Early	Non-grubbing	0.70	0.85	-0.84	2.52	0.58	*0.65*	*-0.07*	*1.38*	-0.44	0.15	-0.85	1.15
	Warming vs Ambient Late vs Peak	Non-grubbing	0.02	*1.41*	*-0.27*	*3.09*	-0.74	*0.64*	*-0.09*	*1.37*	-2.44	0.59	-0.41	1.59
	Warming *vs* Ambient Late *vs* Early	Non-grubbing	0.70	**2.26**	**0.61**	**3.90**	0.58	**1.30**	**0.57**	**2.02**	-0.44	0.74	-0.24	1.73
Random-effects				Std.Dev.	Low CI	Up CI		Std.Dev.	Low CI	Up CI		Std.Dev.	Low CI	Up CI
Plot_ID								0.59	0.36	0.84		0.57	0.25	0.92
Subplot_ID				0.87	0.26	1.60								
Residual				2.17	1.88	2.47		0.94	0.83	1.06		1.22	1.06	1.38
Observations				161				160				142		

TABLE S6. Parameter estimates from the linear mixed-effects models for the effects of spring goose grubbing and summer warming on ***normalized-difference vegetation index (NDVI)*** throughout the summer of 2017. Parameter estimates of fixed-effects (*Est.*) and their 95% confidence interval (CI – lower and upper bounds) for models on NDVI of mesic, moist, and wet habitats. Predictors are ‘spring goose grubbing’ (two-level factor: un-grubbed and grubbed plots), ‘summer warming’ (two-level factor: ambient and warmed plots), and ‘summer season’ (three-level factor: early, peak, and late summer). To allow a complete overview for the reader, intercepts (i.e. reference levels – RL) are calculated for (i) all main contrasts (i.e. main effects) and (ii) all two-way contrasts (i.e. two-way interacting effects) retained in the final models (see main text for details). As the two-way interaction ‘spring goose grubbing × summer warming’ and the three-way interaction ‘spring goose grubbing × summer warming × summer season’ were not retained in the final models (see main text for details), we present (i) the main effects of ‘spring goose grubbing’ and ‘summer warming’ and (ii) their two-way interacting effects with ‘summer season’ by using ‘ambient’ and ‘non-grubbing’ plots, respectively, as reference levels. However, note that estimates and their 95% confidence intervals would be the same if ‘warmed’ and ‘grubbed’ plots, respectively, were used instead. Estimates in bold indicate that their 95% CI does not include zero (i.e. statistically significant effects), while estimates in italic indicate that their 90% CI does not include zero (i.e. marginally significant effects). Random-effects, which are presented as standard deviations, as well as the number of observations for each model, are reported at the bottom of the table.

Table S6.

Fixed-effects	Contrast	Baseline combination for the contrast of interest	Mesic habitats				Moist habitats				Wet habitats			
			RL	Est.	Low CI	Up CI	RL	Est.	Low CI	Up CI	RL	Est.	Low CI	Up CI
(i) Main effects	Grubbing *vs* Non-grubbing	Ambient/Early	0.650	**-0.058**	**-0.083**	**-0.032**	0.678	**-0.076**	**-0.115**	**-0.037**	0.638	-0.024	-0.064	0.017
		Ambient/Peak	0.727	**-0.082**	**-0.108**	**-0.057**	0.779	**-0.090**	**-0.129**	**-0.051**	0.780	**-0.084**	**-0.124**	**-0.044**
		Ambient/Late	0.706	**-0.070**	**-0.096**	**-0.045**	0.682	**-0.079**	**-0.118**	**-0.040**	0.764	-0.032	-0.072	0.008
	Warming *vs* Ambient	Non-grubbing/Early	0.650	**0.031**	**0.005**	**0.056**	0.678	**0.044**	**0.005**	**0.083**	0.638	0.023	-0.017	0.063
		Non-grubbing/Peak	0.727	0.013	-0.013	0.038	0.779	0.018	-0.021	0.057	0.780	0.026	-0.014	0.066
		Non-grubbing/Late	0.706	0.011	-0.015	0.036	0.682	0.028	-0.011	0.067	0.764	0.013	-0.027	0.054
	Summer season: Peak vs Early	Non-grubbing/Ambient	0.650	**0.078**	**0.062**	**0.094**	0.678	**0.101**	**0.073**	**0.129**	0.638	**0.142**	**0.106**	**0.177**
		Grubbing/Ambient	0.592	**0.053**	**0.037**	**0.069**	0.602	**0.087**	**0.059**	**0.115**	0.615	**0.081**	**0.046**	**0.117**
		Non-grubbing/Warming	0.681	**0.059**	**0.043**	**0.075**	0.722	**0.075**	**0.047**	**0.103**	0.661	**0.145**	**0.109**	**0.180**
		Grubbing/Warming	0.623	**0.035**	**0.019**	**0.051**	0.646	**0.061**	**0.033**	**0.089**	0.638	**0.085**	**0.049**	**0.120**
	Summer season: Late vs Peak	Non-grubbing/Ambient	0.727	**-0.021**	**-0.037**	**-0.005**	0.779	**-0.096**	**-0.124**	**-0.068**	0.780	-0.016	-0.051	0.019
		Grubbing/Ambient	0.645	-0.009	-0.025	0.007	0.689	**-0.085**	**-0.113**	**-0.057**	0.696	**0.036**	**0.001**	**0.071**
		Non-grubbing/Warming	0.740	**-0.023**	**-0.039**	**-0.007**	0.797	**-0.086**	**-0.114**	**-0.059**	0.806	-0.029	-0.064	0.007
		Grubbing/Warming	0.658	-0.011	-0.027	0.005	0.707	**-0.075**	**-0.103**	**-0.047**	0.722	0.023	-0.012	0.059
	Summer season: Late *vs* Early	Non-grubbing/Ambient	0.650	**0.057**	**0.040**	**0.073**	0.678	0.005	-0.023	0.032	0.638	**0.126**	**0.090**	**0.161**
		Grubbing/Ambient	0.592	**0.044**	**0.028**	**0.060**	0.602	0.002	-0.026	0.030	0.615	**0.117**	**0.082**	**0.153**
		Non-grubbing/Warming	0.681	**0.037**	**0.020**	**0.053**	0.722	-0.012	-0.039	0.016	0.661	**0.116**	**0.081**	**0.151**
		Grubbing/Warming	0.623	**0.024**	**0.008**	**0.040**	0.646	-0.015	-0.042	0.013	0.638	**0.108**	**0.072**	**0.143**
(ii) Two-way interacting effects	Grubbing vs Non-grubbing Peak vs Early	Ambient	0.650	**-0.024**	**-0.043**	**-0.006**	0.678	-0.014	-0.046	0.018	0.638	**-0.060**	**-0.101**	**-0.020**
	Grubbing vs Non-grubbing Late vs Peak	Ambient	0.727	0.012	-0.007	0.031	0.779	0.011	-0.021	0.043	0.780	**0.052**	**0.011**	**0.093**
	Grubbing *vs* Non-grubbing	Ambient	0.650	-0.012	-0.031	0.006	0.678	-0.003	-0.035	0.029	0.638	-0.008	-0.049	0.033
	Warming vs Ambient Peak vs Early	Non-grubbing	0.650	*-0.018*	*-0.037*	*0.001*	0.678	-0.026	-0.058	0.006	0.638	0.003	-0.038	0.044
	Warming vs Ambient Late vs Peak	Non-grubbing	0.727	-0.002	-0.020	0.017	0.779	0.010	-0.022	0.042	0.780	-0.013	-0.054	0.028
	Warming *vs* Ambient Late *vs* Early	Non-grubbing	0.650	**-0.020**	**-0.039**	**-0.001**	0.678	-0.016	-0.048	0.016	0.638	-0.010	-0.051	0.031
Random-effects				Std.Dev.	Low CI	Up CI		Std.Dev.	Low CI	Up CI		Std.Dev.	Low CI	Up CI
Block				0.037	0.013	0.065						0.064	0.027	0.109
Plot_ID								0.041	0.028	0.055		0.033	0.016	0.051
Subplot_ID				0.041	0.032	0.050								
Residual				0.025	0.022	0.028		0.043	0.038	0.048		0.051	0.044	0.057
Observations				168				168				144		

TABLE S7. The effects of normalized-difference vegetation index (NDVI), soil moisture, and moss-mat temperature (–2 cm) on across-habitat CO_2-fluxes in 2017. Parametric coefficients (Est: estimate; SE: standard error) of the intercept are shown for each model. Degrees of freedom (edf: effective degrees of freedom; ref df: effective degrees of freedom used for hypothesis testing), F values, and the significance level (*P-value*) for the predictors (both fixed- and random-effects) were determined by using analysis of variance on the additive mixed-effects models (see *Material and Methods: Statistical analyses* in the main text for details). Significant ($P < 0.05$) effects are shown in bold. The number of observations (n) is reported for each model.

Model	Parametric coefficient (intercept)	Predictors (smooth terms)		Approximate significance		
				edf, ref df	F	*P-value*
Gross ecosystem productivity (GEP)	*Est* : -2.7380	Fixed-effects:	NDVI	3.088, 3.864	44.9	**2.0E-16**
	SE : 0.1064		Soil moisture	2.968, 3.640	3.5	**0.0115**
	t-value : -25.74		Moss-mat temperature	1.657, 2.046	44.9	**2.0E-16**
(n = 468)	*P-value* : 2.0E-16	Random-effects:	Plot_ID	59.987, 79.000	3.0	**2.0E-16**
Ecosystem respiration (ER)	*Est* : 3.0835	Fixed-effects:	NDVI	1.404, 1.692	8.1	**0.002**
	SE : 0.1521		Soil moisture	2.862, 3.510	7.6	**2.7E-05**
	t-value : 20.27		Moss-mat temperature	1.256, 1.451	9.9	**0.001**
(n = 473)	*P-value* : 2.0E-16	Random-effects:	Plot_ID	57.458, 79.000	2.5	**2.0E-16**
Net ecosystem exchange (NEE)	*Est* : 0.3325	Fixed-effects:	NDVI	2.264, 2.807	10.9	**2.7E-06**
	SE : 0.1147		Soil moisture	2.243, 2.737	20.2	**2.0E-16**
	t-value : 2.90		Moss-mat temperature	1.001, 1.002	8.3	**0.0043**
(n = 465)	*P-value* : 0.0040	Random-effects:	Plot_ID	28.092, 79.000	0.8	**0.0065**
			Subplot_ID	45.027, 159.000	0.5	**0.0100**

TABLE S8. The effects of spring goose grubbing, summer warming, summer season, and their interactions on CO_2-fluxes and normalized-difference vegetation index in 2016. F values and associated degrees of freedom (df; presented in the order of nominator and denominator), as well as the significance level (*P-value*) for the predictors, were determined by using analysis of variance on the linear mixed-effects models (see *Material and Methods: Statistical analyses* in the main text for details and Table S9-S12 for parameter estimates of these models). Significant ($P < 0.05$) and marginally significant ($P < 0.1$) main and interactive effects are shown in bold and italic font, respectively.

Model	Predictors	Mesic habitats		Moist habitats		Wet habitats	
		$F_{(df)}$	*P-value*	$F_{(df)}$	*P-value*	$F_{(df)}$	*P-value*
Gross ecosystem productivity (GEP)	Grubbing	$\mathbf{6.2_{(1,22)}}$	**0.0208**	$\mathbf{13.6_{(1,19)}}$	**0.0015**	$\mathbf{10.1_{(1,128)}}$	**0.0018**
	Warming	$1.7_{(1,22)}$	0.2046	$\mathbf{10.9_{(1,19)}}$	**0.0037**	$\mathit{3.3_{(1,128)}}$	*0.0725*
	Summer season	$\mathbf{12.8_{(2,129)}}$	**8.3E-06**	$\mathbf{55.1_{(2,133)}}$	**2.2E-16**	$\mathbf{24.0_{(2,128)}}$	**1.4E-09**
	Grubbing × Summer season	$1.2_{(2,129)}$	0.3010	$\mathit{2.4_{(2,133)}}$	*0.0944*	$1.2_{(2,128)}$	0.3116
	Warming × Summer season	$\mathbf{3.5_{(2,129)}}$	**0.0328**	$1.5_{(2,133)}$	0.2194	$0.6_{(2,128)}$	0.5519
Ecosystem respiration (ER)	Grubbing	$\mathit{3.0_{(1,47)}}$	*0.0902*	$\mathbf{8.5_{(1,146)}}$	**0.0041**	$2.7_{(1,16)}$	0.1231
	Warming	$\mathbf{13.5_{(1,47)}}$	**0.0006**	$\mathbf{28.3_{(1,146)}}$	**3.8E-07**	$\mathit{3.4_{(1,16)}}$	*0.0832*
	Summer season	$\mathbf{41.7_{(2,98)}}$	**7.8E-14**	$\mathbf{28.8_{(2,146)}}$	**2.8E-11**	$\mathbf{32.9_{(2,112)}}$	**5.8E-12**
	Grubbing × Summer season	$0.7_{(2,98)}$	0.4758	$1.1_{(2,146)}$	0.3505	$0.5_{(2,112)}$	0.5873
	Warming × Summer season	$\mathbf{3.4_{(2,98)}}$	**0.0382**	$0.7_{(2,146)}$	0.5128	$0.4_{(2,112)}$	0.6550
Net ecosystem exchange (NEE)	Grubbing	$1.3_{(1,53)}$	0.2546	$\mathit{3.9_{(1,25)}}$	*0.0611*	$2.3_{(1,127)}$	0.1298
	Warming	$\mathbf{7.3_{(1,53)}}$	**0.0093**	$0.1_{(1,25)}$	0.7494	$<0.1_{(1,127)}$	0.9170
	Summer season	$\mathbf{21.7_{(2,105)}}$	**1.3E-08**	$\mathbf{37.8_{(2,129)}}$	**1.2E-13**	$\mathbf{16.1_{(2,127)}}$	**5.7E-07**
	Grubbing × Summer season	$0.5_{(2,105)}$	0.5794	$1.7_{(2,129)}$	0.1837	$1.0_{(2,127)}$	0.3750
	Warming × Summer season	$3.8_{(2,105)}$	0.2973	$0.2_{(2,129)}$	0.8261	$0.1_{(2,127)}$	0.8675
Normalized-difference vegetation index (NDVI)	Grubbing	$\mathbf{23.8_{(1,47)}}$	**1.3E-05**	$\mathbf{31.1_{(1,19)}}$	**2.2E-05**	$\mathbf{7.5_{(1,16)}}$	**0.0147**
	Warming	$0.4_{(1,47)}$	0.5267	$2.7_{(1,19)}$	0.1159	$\mathit{4.2_{(1,16)}}$	*0.0567*
	Summer season	$\mathbf{129.6_{(2,106)}}$	**2.2E-16**	$\mathbf{77.5_{(2,134)}}$	**2.2E-16**	$\mathbf{100.3_{(2,113)}}$	**2.2E-16**
	Grubbing × Summer season	$1.2_{(2,106)}$	0.2996	$0.8_{(2,134)}$	0.4699	$0.9_{(2,113)}$	0.4250
	Warming × Summer season	$1.2_{(2,106)}$	0.3079	$1.1_{(2,134)}$	0.3511	$0.7_{(2,113)}$	0.5158

TABLE S9. Parameter estimates from the linear mixed-effects models for the effects of spring goose grubbing and summer warming on ***gross ecosystem productivity (GEP)*** throughout the summer of 2016. Parameter estimates of fixed-effects (*Est.*) and their 95% confidence interval (CI – lower and upper bounds) for models on GEP (µmol CO_2 m^{-2} s^{-1}) of mesic, moist, and wet habitats. Predictors are 'spring goose grubbing' (two-level factor: un-grubbed and grubbed plots), 'summer warming' (two-level factor: ambient and warmed plots), and 'summer season' (three-level factor: early, peak, and late summer). To allow a complete overview for the reader, intercepts (i.e. reference levels – RL) are calculated for (i) all main contrasts (i.e. main effects) and (ii) all two-way contrasts (i.e. two-way interacting effects) retained in the final models (see main text for details). As the two-way interaction 'spring goose grubbing × summer warming' and the three-way interaction 'spring goose grubbing × summer warming × summer season' were not retained in the final models (see main text for details), we present (i) the main effects of 'spring goose grubbing' and 'summer warming' and (ii) their two-way interacting effects with 'summer season' by using 'ambient' and 'non-grubbing' plots, respectively, as reference levels. However, note that estimates and their 95% confidence intervals would be the same if 'warmed' and 'grubbed' plots, respectively, were used instead. Estimates in bold indicate that their 95% CI does not include zero (i.e. statistically significant effects), while estimates in italic indicate that their 90% CI does not include zero (i.e. marginally significant effects). Random-effects, which are presented as standard deviations, as well as the number of observations for each model, are reported at the bottom of the table.

Table S9.

Fixed-effects	Contrast	Baseline combination for the contrast of interest	Mesic habitats				Moist habitats				Wet habitats			
			RL	Est.	Low CI	Up CI	RL	Est.	Low CI	Up CI	RL	Est.	Low CI	Up CI
(i) Main effects	Grubbing *vs* Non-grubbing	Ambient/Early	-1.50	0.35	-0.47	1.17	-1.70	*0.63*	*-0.10*	*1.36*	-3.02	**1.46**	**0.16**	**2.75**
		Ambient/Peak	-3.93	**1.13**	**0.33**	**1.93**	-4.39	**1.51**	**0.77**	**2.24**	-6.86	**1.78**	**0.48**	**3.07**
		Ambient/Late	-2.61	0.50	-0.30	1.30	-1.85	0.60	-0.12	1.33	-4.27	0.40	-0.93	1.73
	Warming *vs* Ambient	Non-grubbing/Early	-1.50	**-1.13**	**-1.95**	**-0.31**	-1.70	**-1.26**	**-1.99**	**-0.53**	-3.02	*-1.26*	*-2.55*	*0.04*
		Non-grubbing/Peak	-3.93	0.27	-0.53	1.06	-4.39	**-0.74**	**-1.47**	**-0.01**	-6.86	-0.28	-1.57	1.01
		Non-grubbing/Late	-2.61	-0.18	-0.98	0.62	-1.85	-0.45	-1.18	0.28	-4.27	-0.53	-1.85	0.79
	Summer season: Peak vs Early	Non-grubbing/Ambient	-1.50	**-2.43**	**-3.35**	**-1.51**	-1.70	**-2.69**	**-3.49**	**-1.89**	-3.02	**-3.85**	**-5.43**	**-2.26**
		Grubbing/Ambient	-1.15	**-1.65**	**-2.58**	**-0.72**	-1.07	**-1.82**	**-2.62**	**-1.02**	-1.56	**-3.52**	**-5.11**	**-1.94**
		Non-grubbing/Warming	-2.63	**-1.04**	**-1.94**	**-0.13**	-2.96	**-2.18**	**-2.98**	**-1.38**	-4.27	**-2.87**	**-4.46**	**-1.29**
		Grubbing/Warming	-2.27	-0.26	-1.17	0.65	-2.33	**-1.30**	**-2.12**	**-0.48**	-2.82	**-2.55**	**-4.14**	**-0.96**
	Summer season: Late vs Peak	Non-grubbing/Ambient	-3.93	**1.32**	**0.42**	**2.22**	-4.39	**2.54**	**1.75**	**3.34**	-6.86	**2.60**	**1.01**	**4.19**
		Grubbing/Ambient	-2.80	0.69	-0.21	1.59	-2.88	**1.64**	**0.84**	**2.44**	-5.09	1.22	-0.40	2.84
		Non-grubbing/Warming	-3.66	*0.87*	*-0.03*	*1.77*	-5.13	**2.84**	**2.04**	**3.63**	-7.14	**2.35**	**0.75**	**3.94**
		Grubbing/Warming	-2.53	0.25	-0.65	1.15	-3.63	**1.93**	**1.13**	**2.74**	-5.37	0.97	-0.65	2.58
	Summer season: Late *vs* Early	Non-grubbing/Ambient	-1.50	**-1.11**	**-2.03**	**-0.19**	-1.70	-0.15	-0.95	0.65	-3.02	-1.25	-2.84	0.34
		Grubbing/Ambient	-1.15	**-0.96**	**-1.89**	**-0.02**	-1.07	-0.18	-0.97	0.62	-1.56	**-2.31**	**-3.92**	**-0.69**
		Non-grubbing/Warming	-2.63	-0.16	-1.07	0.74	-2.96	0.66	-0.14	1.46	-4.27	-0.53	-2.12	1.07
		Grubbing/Warming	-2.27	-0.01	-0.92	0.89	-2.33	0.63	-0.18	1.44	-2.82	*-1.58*	*-3.20*	*0.04*
(ii) Two-way interacting effects	Grubbing vs Non-grubbing Peak vs Early	Ambient	-1.50	0.78	-0.28	1.84	-1.70	*0.88*	*-0.05*	*1.81*	-3.02	0.32	-1.51	2.15
	Grubbing vs Non-grubbing Late vs Peak	Ambient	-3.93	-0.63	-1.67	0.41	-4.39	*-0.90*	*-1.83*	*0.02*	-6.86	-1.38	-3.23	0.48
	Grubbing *vs* Non-grubbing	Ambient	-1.50	0.15	-0.91	1.21	-1.70	-0.03	-0.95	0.90	-3.02	-1.06	-2.91	0.80
	Warming vs Ambient Peak vs Early	Non-grubbing	-1.50	**1.39**	**0.33**	**2.45**	-1.70	0.52	-0.41	1.45	-3.02	0.98	-0.86	2.81
	Warming vs Ambient Late vs Peak	Non-grubbing	-3.93	-0.45	-1.49	0.59	-4.39	0.29	-0.63	1.22	-6.86	-0.25	-2.10	1.60
	Warming *vs* Ambient Late *vs* Early	Non-grubbing	-1.50	*0.95*	*-0.11*	*2.00*	-1.70	*0.81*	*-0.12*	*1.73*	-3.02	0.72	-1.13	2.57
Random-effects				Std.Dev.	Low CI	Up CI		Std.Dev.	Low CI	Up CI		Std.Dev.	Low CI	Up CI
Block								0.57	0.14	1.10		1.88	0.72	3.14
Plot_ID				0.41	0.06	0.83		0.42	0.08	0.83				
Subplot_ID														
Residual				1.39	1.23	1.56		1.23	1.09	1.38		2.27	1.98	2.55
Observations				165				166				142		

TABLE S10. Parameter estimates from the linear mixed-effects models for the effects of spring goose grubbing and summer warming on ***ecosystem respiration (ER)*** throughout the summer of 2016. Parameter estimates of fixed-effects (*Est.*) and their 95% confidence interval (CI – lower and upper bounds) for models on ER (µmol CO_2 m^{-2} s^{-1}) of mesic, moist, and wet habitats. Predictors are 'spring goose grubbing' (two-level factor: un-grubbed and grubbed plots), 'summer warming' (two-level factor: ambient and warmed plots), and 'summer season' (three-level factor: early, peak, and late summer). To allow a complete overview for the reader, intercepts (i.e. reference levels – RL) are calculated for (i) all main contrasts (i.e. main effects) and (ii) all two-way contrasts (i.e. two-way interacting effects) retained in the final models (see main text for details). As the two-way interaction 'spring goose grubbing × summer warming' and the three-way interaction 'spring goose grubbing × summer warming × summer season' were not retained in the final models (see main text for details), we present (i) the main effects of 'spring goose grubbing' and 'summer warming' and (ii) their two-way interacting effects with 'summer season' by using 'ambient' and 'non-grubbing' plots, respectively, as reference levels. However, note that estimates and their 95% confidence intervals would be the same if 'warmed' and 'grubbed' plots, respectively, were used instead. Estimates in bold indicate that their 95% CI does not include zero (i.e. statistically significant effects), while estimates in italic indicate that their 90% CI does not include zero (i.e. marginally significant effects). Random-effects, which are presented as standard deviations, as well as the number of observations for each model, are reported at the bottom of the table.

Table S10.

Fixed-effects	Contrast	Baseline combination for the contrast of interest	Mesic habitats				Moist habitats				Wet habitats			
			RL	Est.	Low CI	Up CI	RL	Est.	Low CI	Up CI	RL	Est.	Low CI	Up CI
(i) Main effects	Grubbing *vs* Non-grubbing	Ambient/Early	1.28	-0.14	-1.06	0.78	1.34	-0.19	-0.73	0.36	1.74	-0.70	-1.58	0.19
		Ambient/Peak	4.89	*-0.82*	*-1.74*	*0.09*	3.16	**-0.76**	**-1.33**	**-0.20**	3.28	-0.72	-1.60	0.16
		Ambient/Late	3.36	-0.68	-1.59	0.23	2.71	*-0.46*	*-1.01*	*0.08*	3.13	-0.34	-1.22	0.55
	Warming *vs* Ambient	Non-grubbing/Early	1.28	**1.58**	**0.65**	**2.50**	1.34	**1.05**	**0.50**	**1.59**	1.74	*0.79*	*-0.10*	*1.67*
		Non-grubbing/Peak	4.89	0.29	-0.63	1.20	3.16	**0.93**	**0.36**	**1.50**	3.28	0.44	-0.43	1.32
		Non-grubbing/Late	3.36	**1.63**	**0.72**	**2.54**	2.71	**0.61**	**0.06**	**1.15**	3.13	*0.76*	*-0.12*	*1.64*
	Summer season: Peak vs Early	Non-grubbing/Ambient	1.28	**3.60**	**2.61**	**4.60**	1.34	**1.82**	**1.14**	**2.50**	1.74	**1.54**	**0.83**	**2.26**
		Grubbing/Ambient	1.14	**2.92**	**1.89**	**3.95**	1.16	**1.24**	**0.55**	**1.93**	1.04	**1.52**	**0.81**	**2.23**
		Non-grubbing/Warming	2.86	**2.32**	**1.32**	**3.32**	2.39	**1.70**	**1.01**	**2.39**	2.52	**1.20**	**0.48**	**1.92**
		Grubbing/Warming	2.72	**1.63**	**0.62**	**2.65**	2.21	**1.12**	**0.44**	**1.80**	1.83	**1.17**	**0.46**	**1.89**
	Summer season: Late vs Peak	Non-grubbing/Ambient	4.89	**-1.52**	**-2.52**	**-0.53**	3.16	-0.46	-1.13	0.22	3.28	-0.15	-0.86	0.56
		Grubbing/Ambient	4.07	**-1.38**	**-2.38**	**-0.39**	2.40	-0.16	-0.84	0.53	2.56	0.24	-0.47	0.95
		Non-grubbing/Warming	5.18	-0.18	-1.18	0.81	4.09	**-0.78**	**-1.47**	**-0.08**	3.72	0.17	-0.54	0.88
		Grubbing/Warming	4.35	-0.04	-1.05	0.97	3.33	-0.48	-1.14	0.19	3.00	0.56	-0.17	1.28
	Summer season: Late *vs* Early	Non-grubbing/Ambient	1.28	**2.08**	**1.08**	**3.08**	1.34	**1.36**	**0.70**	**2.03**	1.74	**1.39**	**0.68**	**2.11**
		Grubbing/Ambient	1.14	**1.54**	**0.51**	**2.57**	1.16	**1.08**	**0.42**	**1.75**	1.04	**1.76**	**1.04**	**2.47**
		Non-grubbing/Warming	2.86	**2.13**	**1.14**	**3.13**	2.39	**0.93**	**0.25**	**1.60**	2.52	**1.37**	**0.65**	**2.10**
		Grubbing/Warming	2.72	**1.59**	**0.60**	**2.59**	2.21	*0.65*	*-0.03*	*1.32*	1.83	**1.73**	**1.01**	**2.46**
(ii) Two-way interacting effects	Grubbing vs Non-grubbing Peak vs Early	Ambient	1.28	-0.68	-1.85	0.49	1.34	-0.58	-1.37	0.21	1.74	-0.03	-0.85	0.80
	Grubbing vs Non-grubbing Late vs Peak	Ambient	4.89	0.14	-1.01	1.30	3.16	0.30	-0.49	1.09	3.28	0.39	-0.44	1.21
	Grubbing *vs* Non-grubbing	Ambient	1.28	-0.54	-1.70	0.62	1.34	-0.28	-1.05	0.49	1.74	0.36	-0.47	1.19
	Warming vs Ambient Peak vs Early	Non-grubbing	1.28	**-1.29**	**-2.45**	**-0.12**	1.34	-0.12	-0.91	0.67	1.74	-0.34	-1.17	0.48
	Warming vs Ambient Late vs Peak	Non-grubbing	4.89	**1.34**	**0.19**	**2.50**	3.16	-0.32	-1.11	0.47	3.28	0.32	-0.51	1.15
	Warming *vs* Ambient Late *vs* Early	Non-grubbing	1.28	0.05	-1.11	1.22	1.34	-0.44	-1.21	0.33	1.74	-0.02	-0.85	0.81
Random-effects				Std.Dev.	Low CI	Up CI		Std.Dev.	Low CI	Up CI		Std.Dev.	Low CI	Up CI
Block								0.55	0.22	0.96		0.75	0.20	1.52
Plot_ID												0.77	0.45	1.15
Subplot_ID				0.78	0.40	1.22								
Residual				1.53	1.33	1.74		1.02	0.91	1.14		1.01	0.89	1.15
Observations				165				161				142		

TABLE S11. Parameter estimates from the linear mixed-effects models for the effects of spring goose grubbing and summer warming on ***net ecosystem exchange (NEE)*** throughout the summer of 2016. Parameter estimates of fixed-effects (*Est.*) and their 95% confidence interval (CI – lower and upper bounds) for models on NEE (μmol CO_2 m^{-2} s^{-1}) of mesic, moist, and wet habitats. Predictors are 'spring goose grubbing' (two-level factor: un-grubbed and grubbed plots), 'summer warming' (two-level factor: ambient and warmed plots), and 'summer season' (three-level factor: early, peak, and late summer). To allow a complete overview for the reader, intercepts (i.e. reference levels – RL) are calculated for (i) all main contrasts (i.e. main effects) and (ii) all two-way contrasts (i.e. two-way interacting effects) retained in the final models (see main text for details). As the two-way interaction 'spring goose grubbing × summer warming' and the three-way interaction 'spring goose grubbing × summer warming × summer season' were not retained in the final models (see main text for details), we present (i) the main effects of 'spring goose grubbing' and 'summer warming' and (ii) their two-way interacting effects with 'summer season' by using 'ambient' and 'non-grubbing' plots, respectively, as reference levels. However, note that estimates and their 95% confidence intervals would be the same if 'warmed' and 'grubbed' plots, respectively, were used instead. Estimates in bold indicate that their 95% CI does not include zero (i.e. statistically significant effects), while estimates in italic indicate that their 90% CI does not include zero (i.e. marginally significant effects). Random-effects, which are presented as standard deviations, as well as the number of observations for each model, are reported at the bottom of the table. Positive and negative fluxes denote CO_2 losses (the ecosystem acts as a C source) and CO_2 gains (the ecosystem acts as a C sink), respectively.

Table S11.

Fixed-effects	Contrast	Baseline combination for the contrast of interest	Mesic habitats				Moist habitats				Wet habitats			
			RL	Est.	Low CI	Up CI	RL	Est.	Low CI	Up CI	RL	Est.	Low CI	Up CI
(i) Main effects	Grubbing *vs* Non-grubbing	Ambient/Early	-0.21	0.19	-0.54	0.91	-0.35	0.45	-0.26	1.16	-1.15	0.50	-0.60	1.60
		Ambient/Peak	0.98	0.57	-0.15	1.28	-0.85	**0.95**	**0.22**	**1.67**	-3.59	*1.06*	*-0.04*	*2.16*
		Ambient/Late	0.62	0.13	-0.59	0.86	0.89	0.29	-0.42	1.00	-1.10	-0.07	-1.21	1.07
	Warming *vs* Ambient	Non-grubbing/Early	-0.21	0.46	-0.27	1.18	-0.35	-0.21	-0.92	0.49	-1.15	-0.21	-1.31	0.89
		Non-grubbing/Peak	0.98	0.51	-0.21	1.23	-0.85	-0.07	-0.80	0.65	-3.59	0.16	-0.94	1.26
		Non-grubbing/Late	0.62	**1.10**	**0.37**	**1.82**	0.89	0.01	-0.70	0.72	-1.10	0.15	-0.99	1.28
	Summer season: Peak vs Early	Non-grubbing/Ambient	-0.21	**1.19**	**0.41**	**1.97**	-0.35	-0.49	-1.12	0.13	-1.15	**-2.43**	**-3.78**	**-1.09**
		Grubbing/Ambient	-0.03	**1.57**	**0.80**	**2.34**	0.10	0.01	-0.62	0.63	-0.65	**-1.88**	**-3.22**	**-0.53**
		Non-grubbing/Warming	0.25	**1.25**	**0.47**	**2.02**	-0.56	-0.36	-1.00	0.29	-1.36	**-2.06**	**-3.41**	**-0.71**
		Grubbing/Warming	0.43	**1.63**	**0.85**	**2.40**	-0.11	0.14	-0.49	0.77	-0.86	**-1.51**	**-2.85**	**-0.16**
	Summer season: Late vs Peak	Non-grubbing/Ambient	0.98	-0.36	-1.13	0.41	-0.85	**1.74**	**1.11**	**2.37**	-3.59	**2.49**	**1.13**	**3.84**
		Grubbing/Ambient	1.54	**-0.79**	**-1.56**	**-0.02**	0.10	**1.08**	**0.45**	**1.71**	-2.53	*1.36*	*-0.01*	*2.74*
		Non-grubbing/Warming	1.49	0.23	-0.56	1.01	-0.92	**1.82**	**1.18**	**2.47**	-3.42	**2.47**	**1.12**	**3.83**
		Grubbing/Warming	2.06	-0.21	-0.98	0.56	0.03	**1.16**	**0.53**	**1.79**	-2.37	*1.35*	*-0.05*	*2.75*
	Summer season: Late *vs* Early	Non-grubbing/Ambient	-0.21	**0.83**	**0.04**	**1.61**	-0.35	**1.25**	**0.63**	**1.86**	-1.15	0.06	-1.30	1.41
		Grubbing/Ambient	-0.03	**0.78**	**0.00**	**1.55**	0.10	**1.08**	**0.47**	**1.70**	-0.65	-0.51	-1.89	0.86
		Non-grubbing/Warming	0.25	**1.47**	**0.69**	**2.26**	-0.56	**1.47**	**0.84**	**2.09**	-1.36	0.41	-0.94	1.77
		Grubbing/Warming	0.43	**1.42**	**0.64**	**2.19**	-0.11	**1.30**	**0.69**	**1.92**	-0.86	-0.16	-1.56	1.24
(ii) Two-way interacting effects	Grubbing vs Non-grubbing Peak vs Early	Ambient	-0.21	0.38	-0.52	1.27	-0.35	0.50	-0.23	1.23	-1.15	0.56	-1.00	2.11
	Grubbing vs Non-grubbing Late vs Peak	Ambient	0.98	-0.43	-1.33	0.46	-0.85	*-0.66*	*-1.39*	*0.07*	-3.59	-1.13	-2.71	0.46
	Grubbing *vs* Non-grubbing	Ambient	-0.21	-0.05	-0.95	0.85	-0.35	-0.16	-0.87	0.55	-1.15	-0.57	-2.15	1.02
	Warming vs Ambient Peak vs Early	Non-grubbing	-0.21	0.38	-0.52	1.27	-0.35	0.14	-0.59	0.87	-1.15	0.37	-1.18	1.93
	Warming vs Ambient Late vs Peak	Non-grubbing	0.98	-0.05	-0.95	0.85	-0.85	0.22	-0.49	0.93	-3.59	-0.02	-1.60	1.57
	Warming *vs* Ambient Late *vs* Early	Non-grubbing	-0.21	-0.43	-1.33	0.46	-0.35	0.08	-0.65	0.81	-1.15	0.36	-1.22	1.94
Random-effects				Std.Dev.	Low CI	Up CI		Std.Dev.	Low CI	Up CI		Std.Dev.	Low CI	Up CI
Block												1.51	0.60	2.62
Plot_ID								0.65	0.40	0.91				
Subplot_ID				0.66	0.38	0.98								
Residual				1.19	1.03	1.35		0.95	0.83	1.06		1.93	1.69	2.16
Observations				166				162				141		

TABLE S12. Parameter estimates from the linear mixed-effects models for the effects of spring goose grubbing and summer warming on ***normalized-difference vegetation index (NDVI)*** throughout the summer of 2016. Parameter estimates of fixed-effects (*Est.*) and their 95% confidence interval (CI – lower and upper bounds) for models on NDVI of mesic, moist, and wet habitats. Predictors are ‘spring goose grubbing’ (two-level factor: un-grubbed and grubbed plots), ‘summer warming’ (two-level factor: ambient and warmed plots), and ‘summer season’ (three-level factor: early, peak, and late summer). To allow a complete overview for the reader, intercepts (i.e. reference levels – RL) are calculated for (i) all main contrasts (i.e. main effects) and (ii) all two-way contrasts (i.e. two-way interacting effects) retained in the final models (see main text for details). As the two-way interaction ‘spring goose grubbing × summer warming’ and the three-way interaction ‘spring goose grubbing × summer warming × summer season’ were not retained in the final models (see main text for details), we present (i) the main effects of ‘spring goose grubbing’ and ‘summer warming’ and (ii) their two-way interacting effects with ‘summer season’ by using ‘ambient’ and ‘non-grubbing’ plots, respectively, as reference levels. However, note that estimates and their 95% confidence intervals would be the same if ‘warmed’ and ‘grubbed’ plots, respectively, were used instead. Estimates in bold indicate that their 95% CI does not include zero (i.e. statistically significant effects), while estimates in italic indicate that their 90% CI does not include zero (i.e. marginally significant effects). Random-effects, which are presented as standard deviations, as well as the number of observations for each model, are reported at the bottom of the table.

Table S12.

Fixed-effects	Contrast	Baseline combination for the contrast of interest	Mesic habitats				Moist habitats				Wet habitats			
			RL	Est.	Low CI	Up CI	RL	Est.	Low CI	Up CI	RL	Est.	Low CI	Up CI
(i) Main effects	Grubbing *vs* Non-grubbing	Ambient/Early	0.662	**-0.064**	**-0.089**	**-0.038**	0.692	**-0.058**	**-0.083**	**-0.032**	0.674	-0.021	-0.052	0.011
		Ambient/Peak	0.740	**-0.054**	**-0.080**	**-0.029**	0.774	**-0.066**	**-0.092**	**-0.040**	0.820	**-0.044**	**-0.075**	**-0.013**
		Ambient/Late	0.689	**-0.048**	**-0.073**	**-0.022**	0.703	**-0.050**	**-0.076**	**-0.024**	0.773	*-0.029*	*-0.060*	*0.002*
	Warming *vs* Ambient	Non-grubbing/Early	0.662	0.003	-0.023	0.028	0.692	**0.027**	**0.002**	**0.053**	0.674	**0.035**	**0.004**	**0.067**
		Non-grubbing/Peak	0.740	0.002	-0.023	0.028	0.774	0.015	-0.011	0.041	0.820	0.019	-0.012	0.051
		Non-grubbing/Late	0.689	0.016	-0.009	0.042	0.703	0.009	-0.017	0.035	0.773	0.016	-0.015	0.047
	Summer season: Peak vs Early	Non-grubbing/Ambient	0.662	**0.078**	**0.060**	**0.096**	0.692	**0.083**	**0.061**	**0.105**	0.674	**0.146**	**0.115**	**0.177**
		Grubbing/Ambient	0.598	**0.087**	**0.070**	**0.105**	0.634	**0.074**	**0.052**	**0.096**	0.653	**0.123**	**0.092**	**0.154**
		Non-grubbing/Warming	0.665	**0.077**	**0.060**	**0.095**	0.719	**0.070**	**0.048**	**0.092**	0.709	**0.130**	**0.099**	**0.161**
		Grubbing/Warming	0.601	**0.087**	**0.069**	**0.104**	0.662	**0.062**	**0.039**	**0.084**	0.689	**0.107**	**0.075**	**0.138**
	Summer season: Late vs Peak	Non-grubbing/Ambient	0.740	**-0.051**	**-0.068**	**-0.033**	0.774	**-0.072**	**-0.094**	**-0.050**	0.820	**-0.047**	**-0.078**	**-0.016**
		Grubbing/Ambient	0.685	**-0.044**	**-0.062**	**-0.026**	0.708	**-0.056**	**-0.078**	**-0.034**	0.776	**-0.032**	**-0.063**	**-0.001**
		Non-grubbing/Warming	0.742	**-0.037**	**-0.054**	**-0.019**	0.789	**-0.077**	**-0.099**	**-0.055**	0.839	**-0.051**	**-0.082**	**-0.020**
		Grubbing/Warming	0.688	**-0.030**	**-0.048**	**-0.012**	0.723	**-0.062**	**-0.084**	**-0.039**	0.795	**-0.036**	**-0.067**	**-0.005**
	Summer season: Late *vs* Early	Non-grubbing/Ambient	0.662	**0.028**	**0.010**	**0.045**	0.692	0.011	-0.011	0.033	0.674	**0.099**	**0.068**	**0.130**
		Grubbing/Ambient	0.598	**0.043**	**0.026**	**0.061**	0.634	0.018	-0.004	0.040	0.653	**0.091**	**0.060**	**0.122**
		Non-grubbing/Warming	0.665	**0.041**	**0.023**	**0.059**	0.719	-0.007	-0.029	0.015	0.709	**0.079**	**0.048**	**0.110**
		Grubbing/Warming	0.601	**0.057**	**0.039**	**0.074**	0.662	-0.001	-0.022	0.022	0.689	**0.071**	**0.039**	**0.103**
(ii) Two-way interacting effects	Grubbing vs Non-grubbing Peak vs Early	Ambient	0.662	0.009	-0.011	0.030	0.692	-0.009	-0.034	0.017	0.674	-0.023	-0.060	0.013
	Grubbing vs Non-grubbing Late vs Peak	Ambient	0.740	0.007	-0.014	0.027	0.774	0.016	-0.010	0.041	0.820	0.015	-0.021	0.051
	Grubbing *vs* Non-grubbing	Ambient	0.662	0.016	-0.004	0.036	0.692	0.007	-0.018	0.033	0.674	-0.008	-0.044	0.028
	Warming vs Ambient Peak vs Early	Non-grubbing	0.662	-0.001	-0.021	0.020	0.692	-0.013	-0.038	0.013	0.674	-0.016	-0.052	0.020
	Warming vs Ambient Late vs Peak	Non-grubbing	0.740	0.014	-0.006	0.034	0.774	-0.006	-0.031	0.020	0.820	-0.004	-0.040	0.032
	Warming *vs* Ambient Late *vs* Early	Non-grubbing	0.662	0.013	-0.007	0.034	0.692	-0.018	-0.044	0.007	0.674	-0.020	-0.056	0.016
Random-effects				Std.Dev.	Low CI	Up CI		Std.Dev.	Low CI	Up CI		Std.Dev.	Low CI	Up CI
Block				0.042	0.015	0.072		0.026	0.009	0.047		0.077	0.032	0.130
Plot_ID								0.024	0.014	0.034		0.021	0.009	0.037
Subplot_ID				0.039	0.030	0.048								
Residual				0.027	0.024	0.031		0.034	0.030	0.038		0.044	0.039	0.050
Observations				168				168				143		

TABLE S13. The effects of normalized-difference vegetation index (NDVI), soil moisture, and moss-mat temperature (–2 cm) on across-habitat CO_2-fluxes in 2016. Parametric coefficients (Est: estimate; SE: standard error) of the intercept are shown for each model. Degrees of freedom (edf: effective degrees of freedom; ref df: effective degrees of freedom used for hypothesis testing), F values, and the significance level (*P-value*) for the predictors (both fixed- and random-effects) were determined by using analysis of variance on the additive mixed-effects models (see *Material and Methods: Statistical analyses* in the main text for details). Significant ($P < 0.05$) and marginally significant ($P < 0.1$) effects are shown in bold and italic font, respectively. The number of observations (n) is reported for each model.

Model	Parametric coefficient (intercept)	Predictors (smooth terms)		Approximate significance		
				edf, ref df	F	*P-value*
Gross ecosystem productivity (GEP)	*Est* : -3.1553	Fixed-effects:	NDVI	5.045, 6.180	69.5	**2.0E-16**
	SE : 0.2511		Soil moisture	2.535, 3.190	2.3	*0.0698*
	t-value : -12.57		Moss-mat temperature	1.453, 1.777	30.6	**2.0E-16**
(n = 472)	*P-value* : 2.0E-16	Random-effects:	Site	5.503, 6.000	11.4	**2.0E-16**
Ecosystem respiration (ER)	*Est* : 3.0671	Fixed-effects:	NDVI	1.001, 1.002	50.7	**2.0E-16**
	SE : 0.1137		Soil moisture	2.664, 3.261	16.5	**2.0E-16**
	t-value : 26.97		Moss-mat temperature	2.626, 3.215	4.6	**0.003**
(n = 471)	*P-value* : 2.0E-16	Random-effects:	Plot_ID	37.612, 79.000	0.9	**3.7E-05**
Net ecosystem exchange (NEE)	*Est* : -0.04839	Fixed-effects:	NDVI	4.155, 5.126	13.1	**2.0E-16**
	SE : 0.15836		Soil moisture	1.003, 1.005	50.1	**2.0E-16**
	t-value : -0.31		Moss-mat temperature	2.750, 3.383	4.1	**0.0070**
(n = 471)	*P-value* : 0.7600	Random-effects:	Block	10.856, 19.000	2.7	**0.0007**
			Plot_ID	25.254, 79.000	0.5	**0.0077**

Supplementary Figures

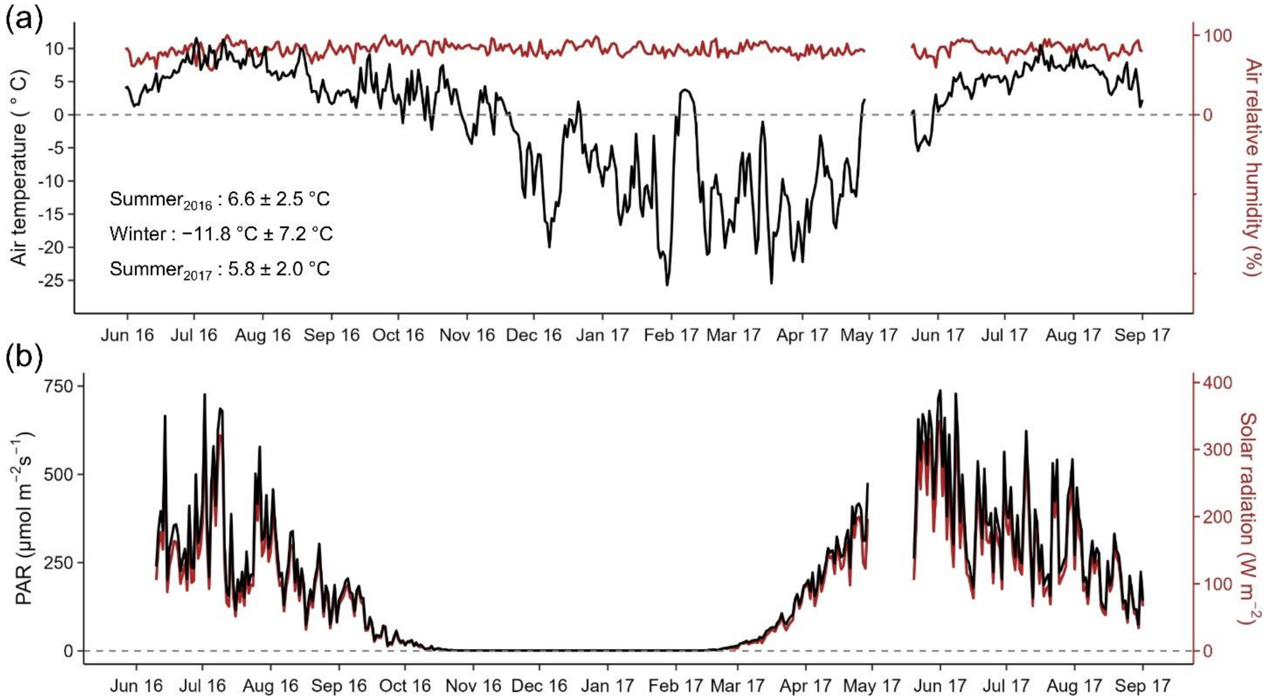

FIGURE S1. *In-situ* environmental characteristics of the study area throughout the study period (from 01-June 2016 to 31-August 2017). Daily average **(a)** air temperature and air relative humidity and **(b)** photosynthetically active radiation (PAR) and solar radiation registered 2 m aboveground by an automated weather station located in the center of the study area (logging interval: 15 min). The weather station was equipped with a 4-channel Micro Station data logger (H21-002, HOBO Onset Computer Corporation, Bourne, Massachusetts, USA) and sensors for environmental data collection (air temperature and relative humidity: S-THB-M002; PAR: S-LIA-M003; and solar radiation: S-LIB-M003). Sensors for air temperature and relative humidity were equipped with solar radiation shields. In (a), daily average (± standard deviation) summer (June to August) and winter (January to March) temperatures are displayed. In 2016, (b) PAR and solar radiation were registered from 10-June. In 2017, (a,b) data are missing from 30-April to 19-May due to a malfunctioning of the weather station. Note that data for summer 2016 and summer 2017 are also presented in the main text (Figure 1) but reported here for completeness.

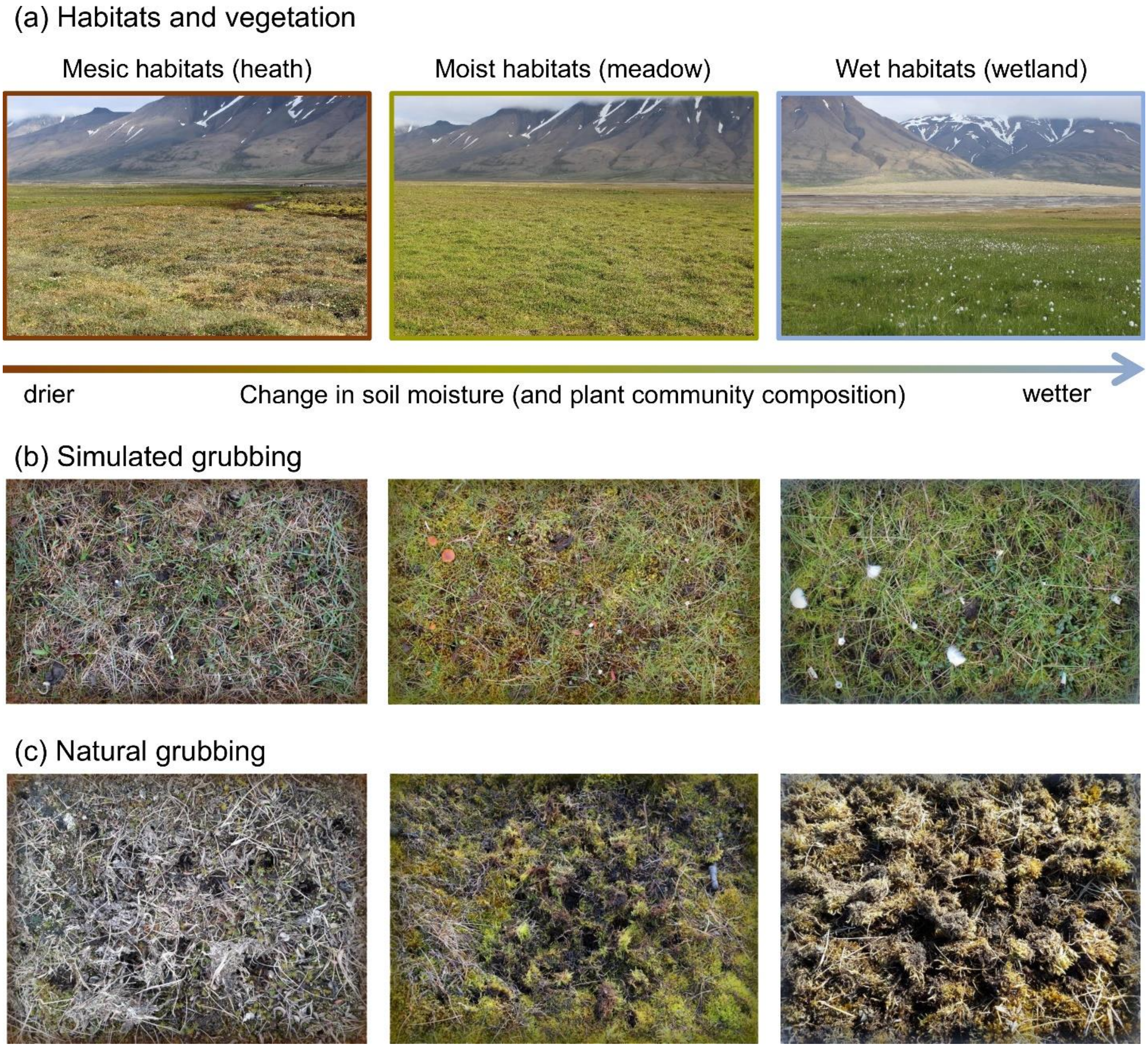

FIGURE S2. Overview of the three habitats and of the simulated and natural spring grubbing by pink-footed geese. **(a)** Example of mesic (heath vegetation), moist (meadow vegetation) and wet (wetland vegetation) habitats used in the experiment; photos: July 2017. **(b)** Example of the spring goose grubbing simulation applied to experimental plots of mesic, moist, and wet habitats, as found at the peak of the growing season; photos: July 2017. **(c)** Example of naturally grubbed tundra-patches in mesic, moist, and wet habitats, as found in spring soon after snowmelt; photos: June 2017. Note that (a) habitat photos are also presented in the main text (Figure 1) but reported here for completeness. Photo credits: Matteo Petit Bon.

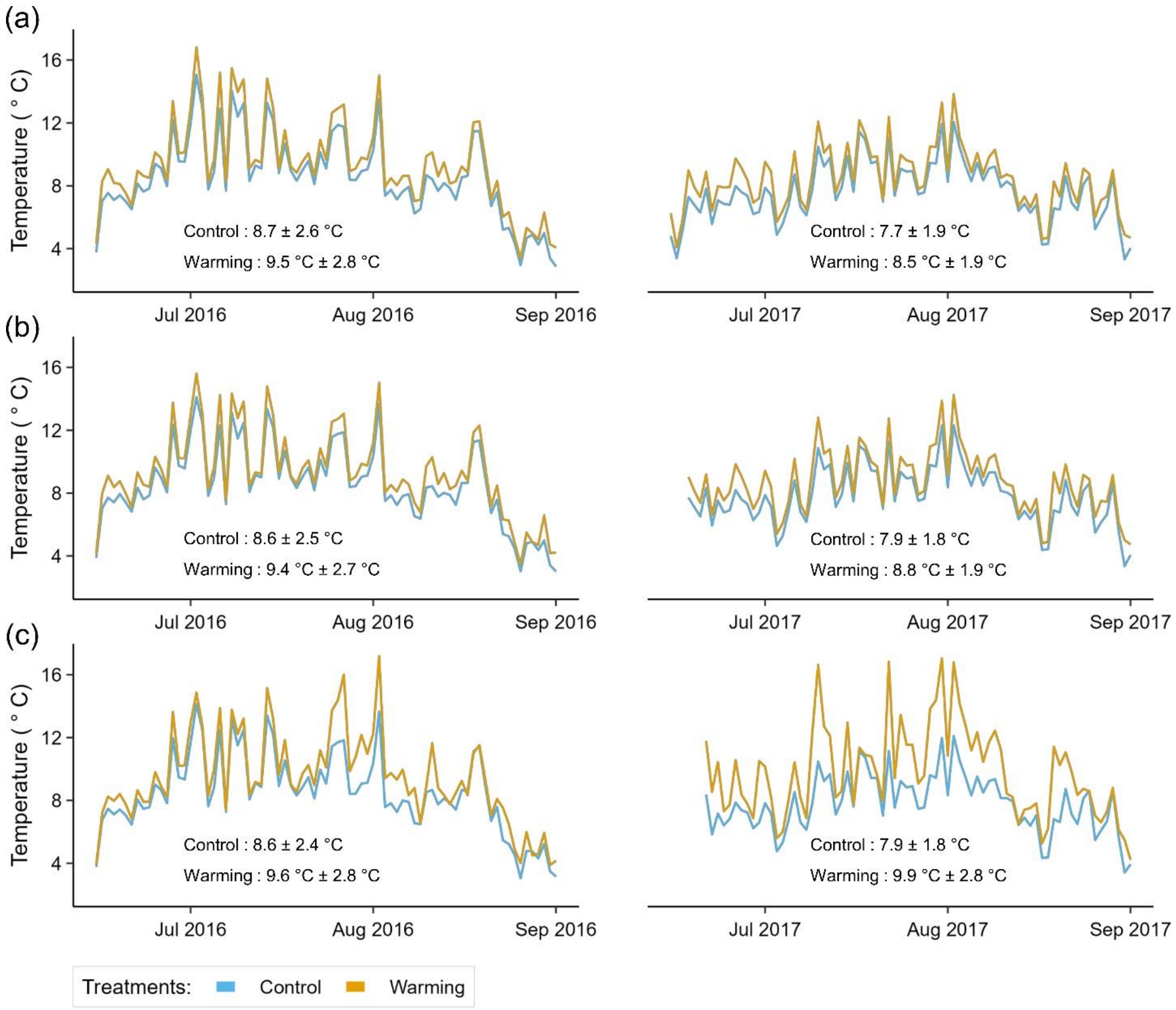

FIGURE S3. Daily plot-level *air temperatures* in control and warming treatments. Daily (from 900 to 1700 h, that is, the same time of CO_2-flux measurements; *cf.* main text) average air (+10 cm from the moss surface) temperatures in **(a)** mesic, **(b)** moist, and **(c)** wet habitats between 15-June and 31-August of 2016 (left panels) and 2017 (right panels), separately for control and warming treatments. Note that air temperature was only monitored in non-grubbing plots. Daily average (± standard deviation) temperatures throughout this period for the two treatments are displayed on each panel. Data (logging interval: 30 min) were registered in 3 replicate plots of each 'treatment × habitat' combination by temperature loggers (U23-003 and UA-001, HOBO Onset Computer Corporation, Bourne, Massachusetts, USA) equipped with solar radiation shields.

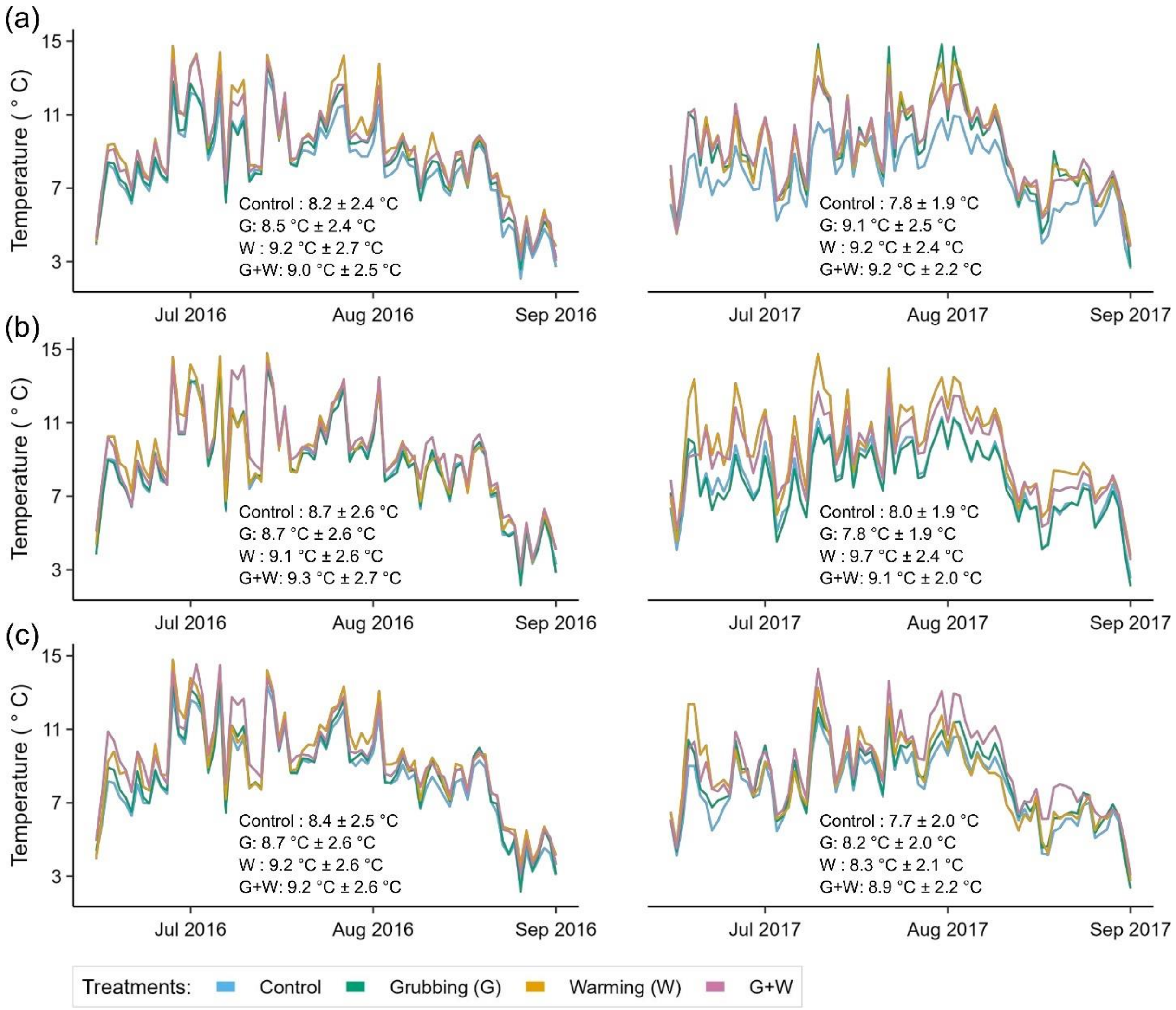

FIGURE S4. Daily plot-level *moss-layer temperatures* in the four treatment combinations. Daily (from 900 to 1700 h, that is, the same time of CO_2-flux measurements; *cf.* main text) average moss-layer (–2 cm from the moss surface) temperatures in **(a)** mesic, **(b)** moist, and **(c)** wet habitats between 15-June and 31-August of 2016 (left panels) and 2017 (right panels), separately for the four treatment combinations: no grubbing and ambient temperature (control); grubbing and ambient temperature; no grubbing and warming; and grubbing and warming. Daily average (± standard deviation) temperatures throughout this period for the four treatments are displayed on each panel. Data (logging interval: 2 h) were registered in 4 replicate plots of each ‘treatment × habitat’ combination by temperature loggers (DS1921G-F5, Maxim Integrated, San Jose, California, USA).

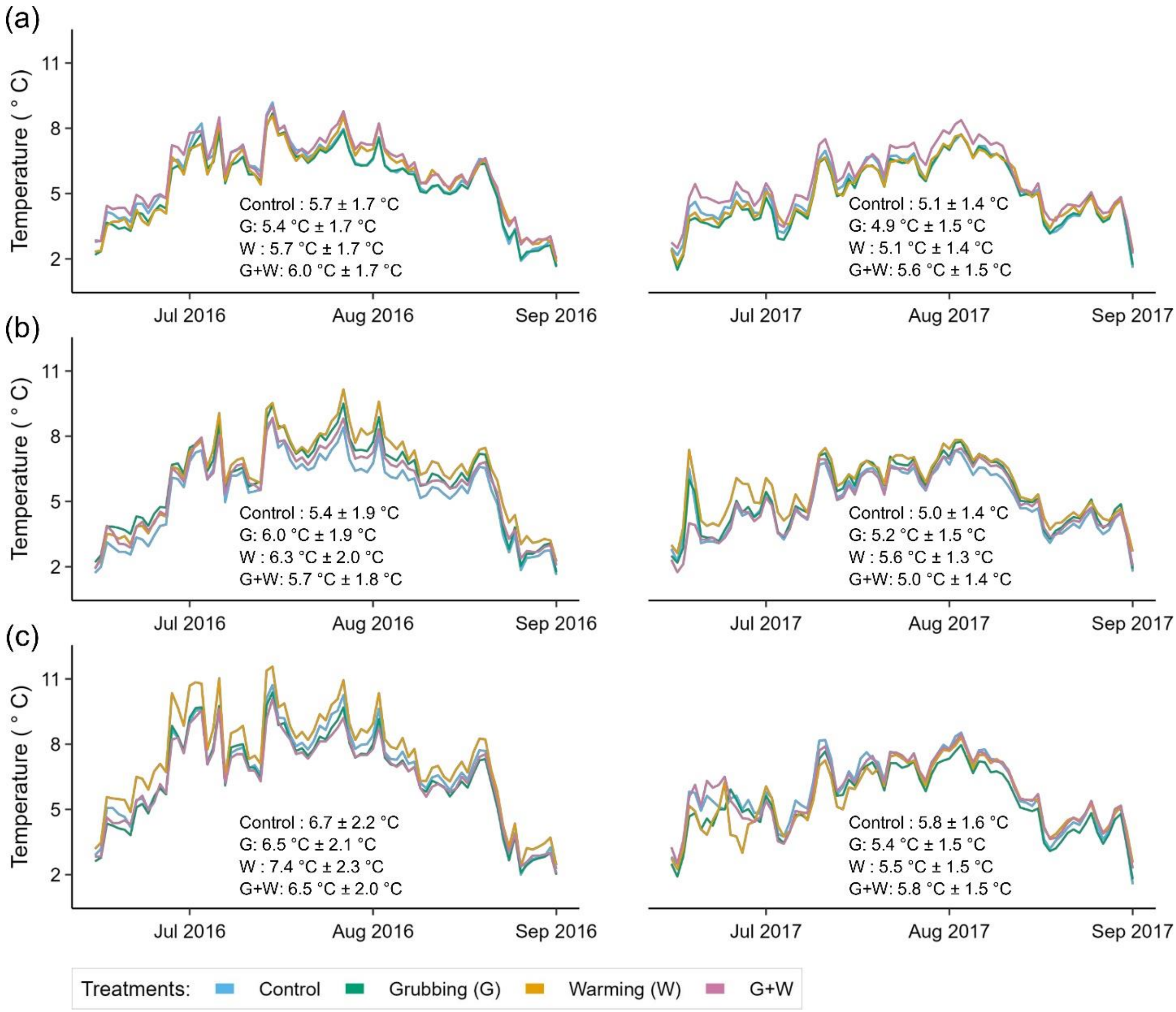

FIGURE S5. Daily plot-level *soil temperatures* in the four treatment combinations. Daily (from 900 to 1700 h, that is, the same time of CO_2-flux measurements; *cf.* main text) average soil (–7 cm from the moss surface) temperatures in **(a)** mesic, **(b)** moist, and **(c)** wet habitats between 15-June and 31-August of 2016 (left panels) and 2017 (right panels), separately for the four treatment combinations: no grubbing and ambient temperature (control); grubbing and ambient temperature; no grubbing and warming; and grubbing and warming. Daily average (± standard deviation) temperatures throughout this period for the four treatments are displayed on each panel. Data (logging interval: 2 h) were registered in 4 replicate plots of each ‘treatment × habitat’ combination by temperature loggers (DS1921G-F5, Maxim Integrated, San Jose, California, USA).

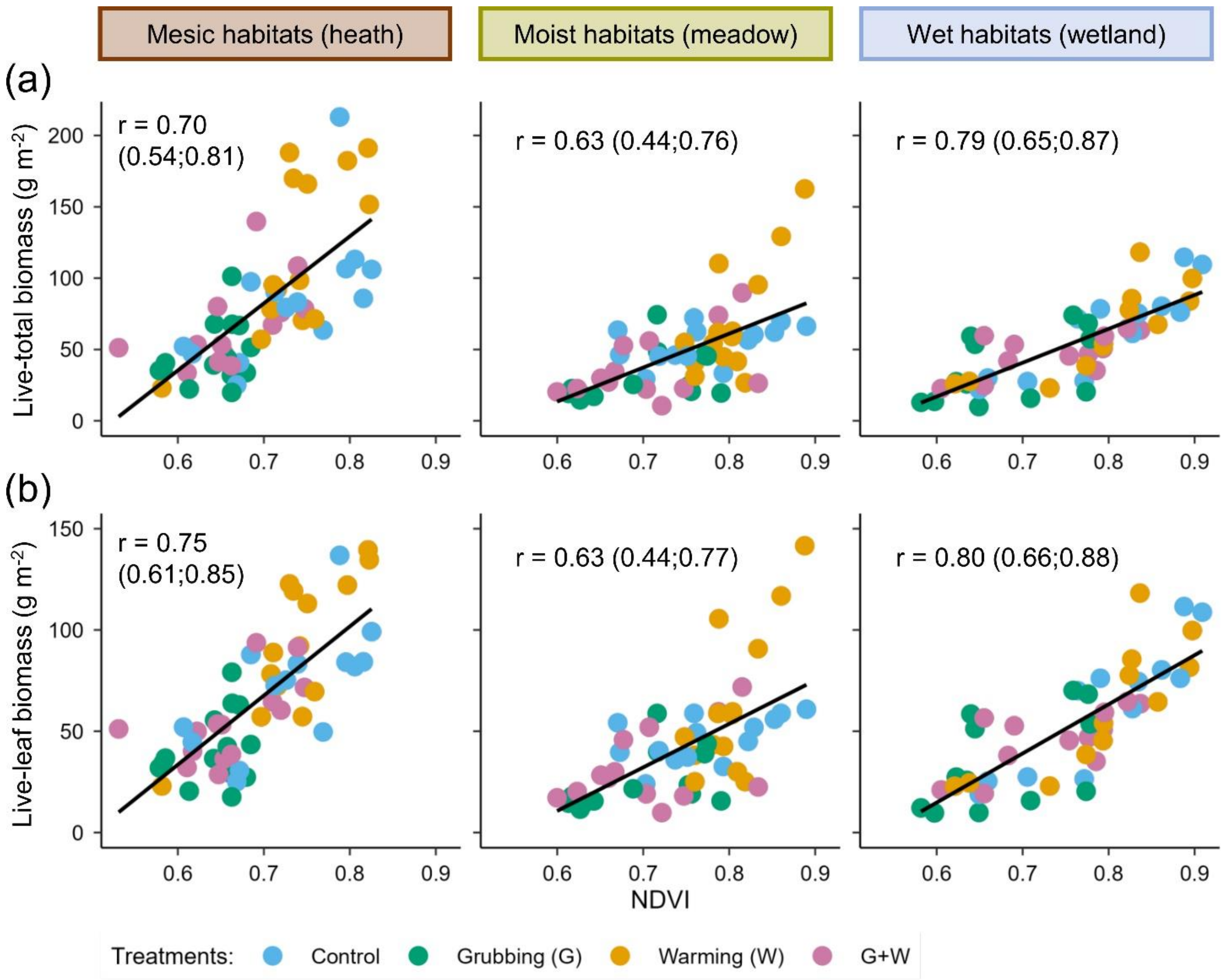

FIGURE S6. Normalized-difference vegetation index (NDVI) as a proxy for live aboveground plant biomass (g m^{-2} dry weight). Relationships between **(a)** subplot live-total biomass (leaves and woody parts) and **(b)** subplot live-leaf biomass and subplot NDVI within the experiment at peak plant growing season (21-28 July) in 2017, separately for mesic, moist, and wet habitats. The four treatment combinations are used as a coding variable. NDVI data were collected within the present study (cf. main text), whereas biomass data (obtained using the point intercept method [PIM]; Bråthen & Hagberg, 2004) are from Petit Bon et al. (2023a). Briefly, PIM was performed in the two subplots at each plot and data were converted into aboveground plant biomass using the conversion coefficients in Petit Bon et al. (2021); see Appendix S1: Section S1 for more details. Pearson correlation coefficients (r) and their 95% confidence intervals for the relationships are given on each panel.

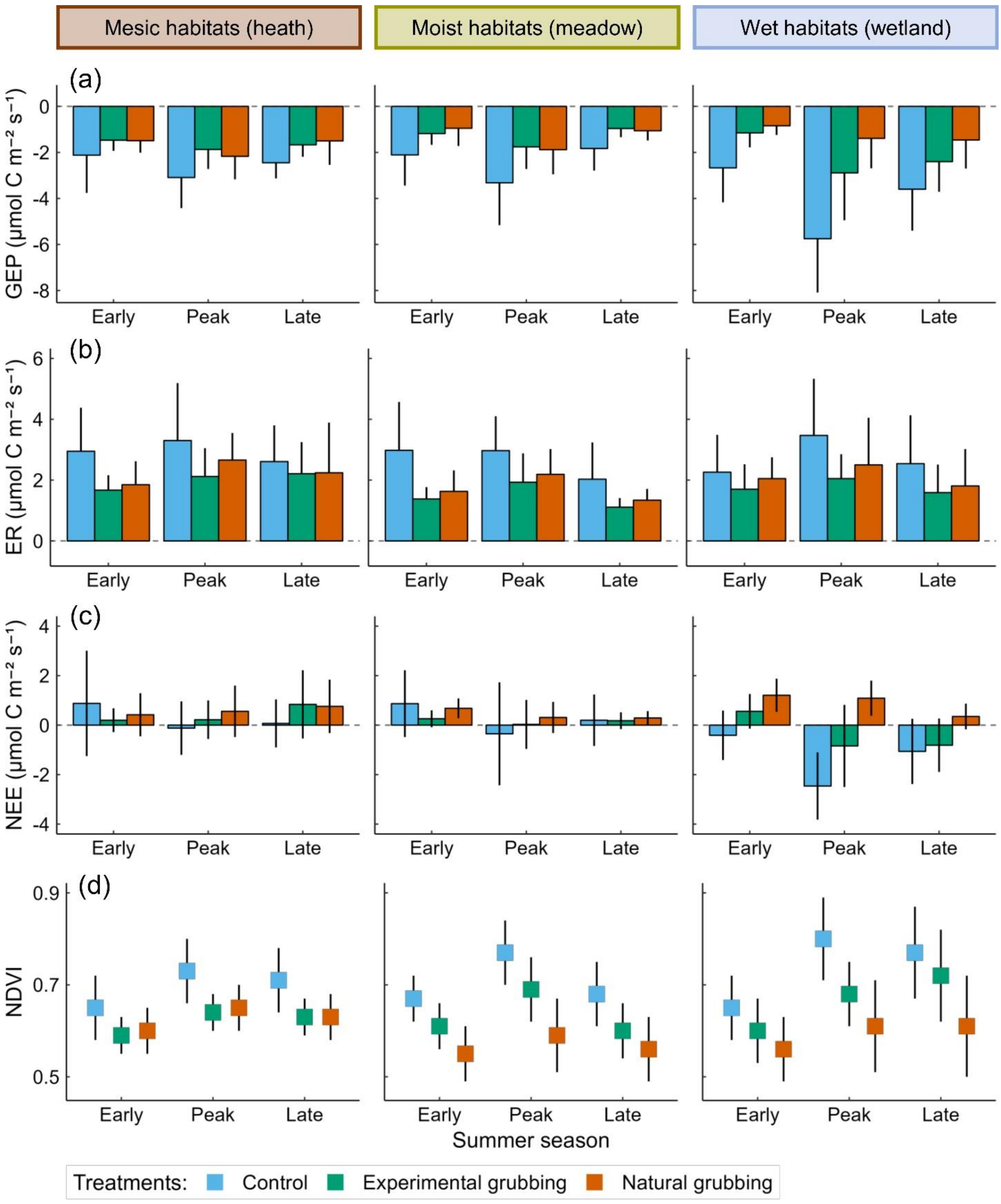

FIGURE S7. Based on our directional hypotheses, the effect of experimental grubbing was either similar or weaker than that of natural grubbing. Average ± standard deviation **(a)** gross ecosystem productivity (GEP), **(b)** ecosystem respiration (ER), **(c)** net ecosystem exchange (NEE), and **(d)** normalized-difference vegetation index (NDVI) in un-manipulated control,

experimentally grubbed and naturally grubbed plots in summer 2017, separately for the three habitats and sampling occasions (note that the effect of natural grubbing was only assessed in 2017). Positive and negative fluxes denote CO_2 losses (the ecosystem acts as a C source) and CO_2 gains (the ecosystem acts as a C sink), respectively. At each habitat and sampling occasion, GEP, ER, NEE, and NDVI were measured in the two subplots at each control and experimentally grubbed plot (n=14 for mesic and moist habitats; n=12 for wet habitats) and in the four naturally grubbed plots selected within each of three of the seven replicate sites (n=12); for details, see *Material and Methods: Data collection and processing* in the main text.

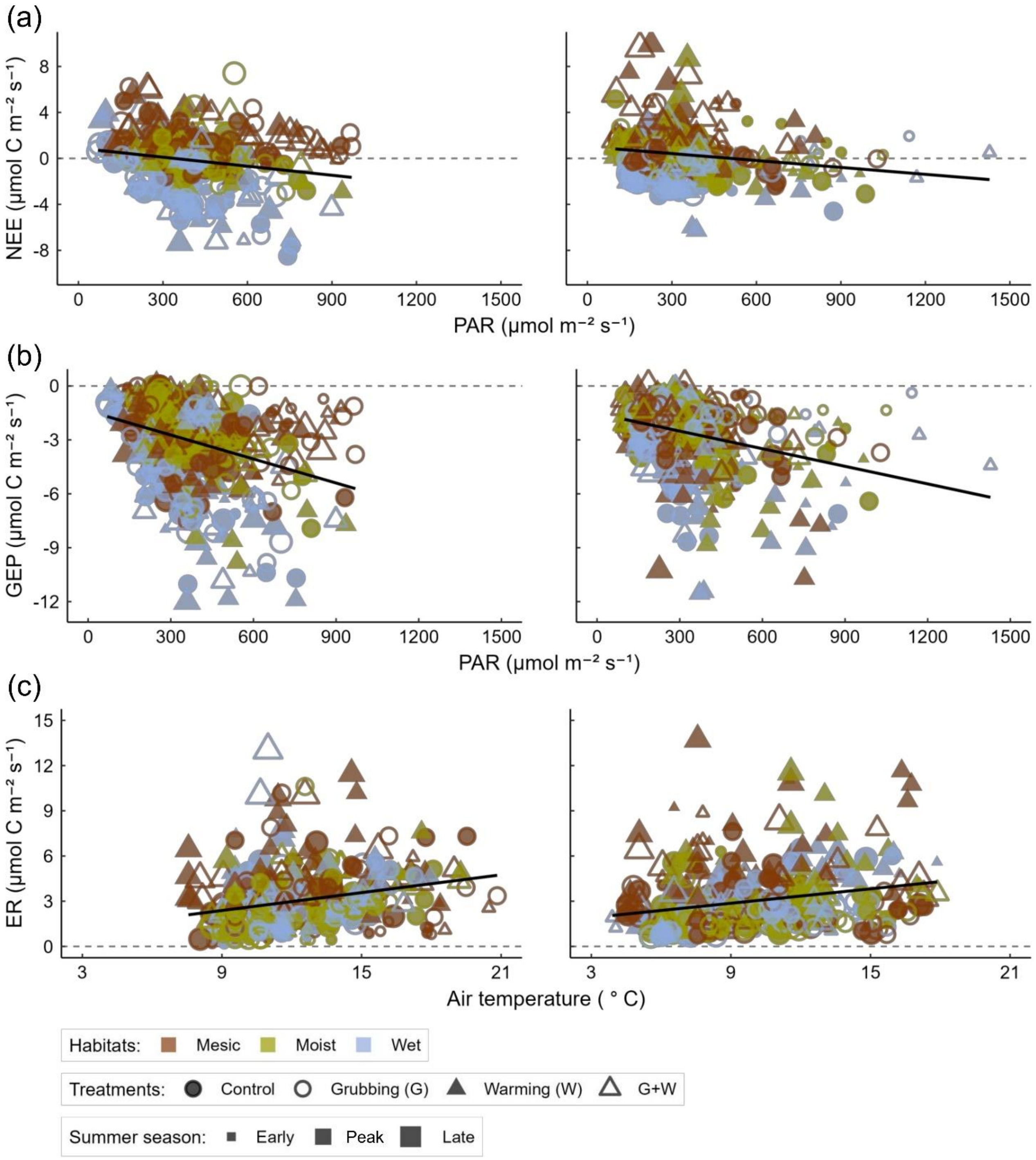

FIGURE S8. High variability around the relationships between within-chamber abiotic variables (i.e. potential covariates initially considered in modelling) and CO_2-fluxes. Relationships between **(a)** net ecosystem exchange (NEE) and photosynthetically active radiation (PAR), **(b)** gross ecosystem productivity (GEP) and PAR, and **(c)** ecosystem respiration (ER) and air temperature, separately for 2016 (left panels) and 2017 (right panels). Habitat, treatment, and sampling occasion were used as coding variables. A similarly high

variation in these relationships was observed when they were explored separately by treatments, habitats, or sampling occasions (results not shown). For graphical purposes, (a) seven data points (fluxes higher than 10 and lower than –10 µmol CO_2 m^{-2} s^{-1}), (b) two data points (fluxes lower than –13 µmol CO_2 m^{-2} s^{-1}), and (c) three data points (fluxes higher than 15 µmol CO_2 m^{-2} s^{-1}) were omitted from the figure, yet the displayed relationships are based on all data. Positive and negative fluxes denote CO_2 losses (the ecosystem acts as a C source) and CO_2 gains (the ecosystem acts as a C sink), respectively.

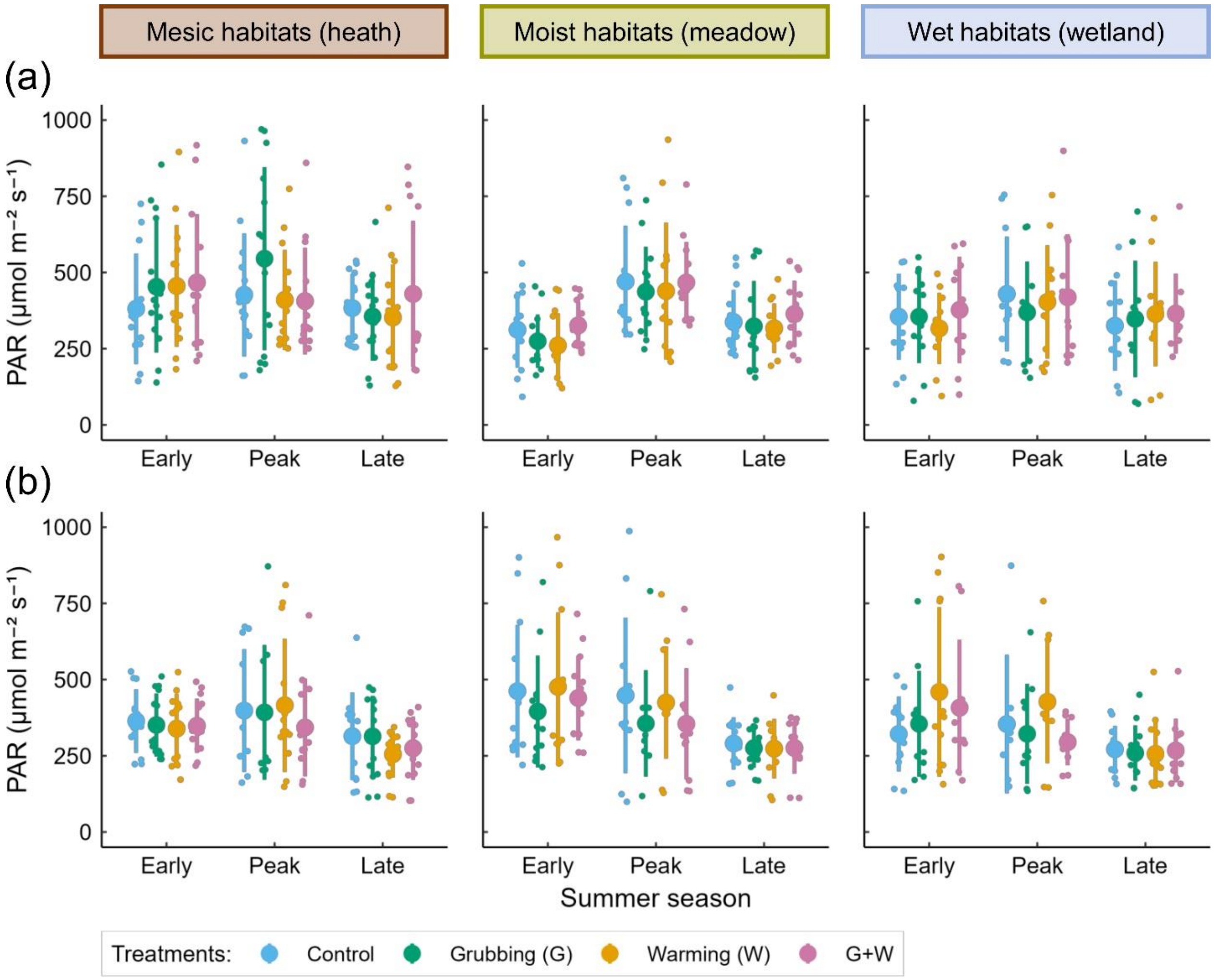

FIGURE S9. Differences in photosynthetically active radiation (PAR) among treatment and habitat CO_2-flux measurements were small. Average ± standard deviation (SD) PAR during net ecosystem exchange (light) measurements in each treatment combination across the summer of **(a)** 2016 and **(b)** 2017, separately for the three habitats. PAR was registered every 5 s within the chamber concurrently with air CO_2 concentration (cf. main text). The dots in the background represent the average PAR during each light measurement. Five data points (>1000 µmol m^{-2} s^{-1}) were omitted from the figure for graphical purposes, yet the displayed averages and SDs are based on all data.

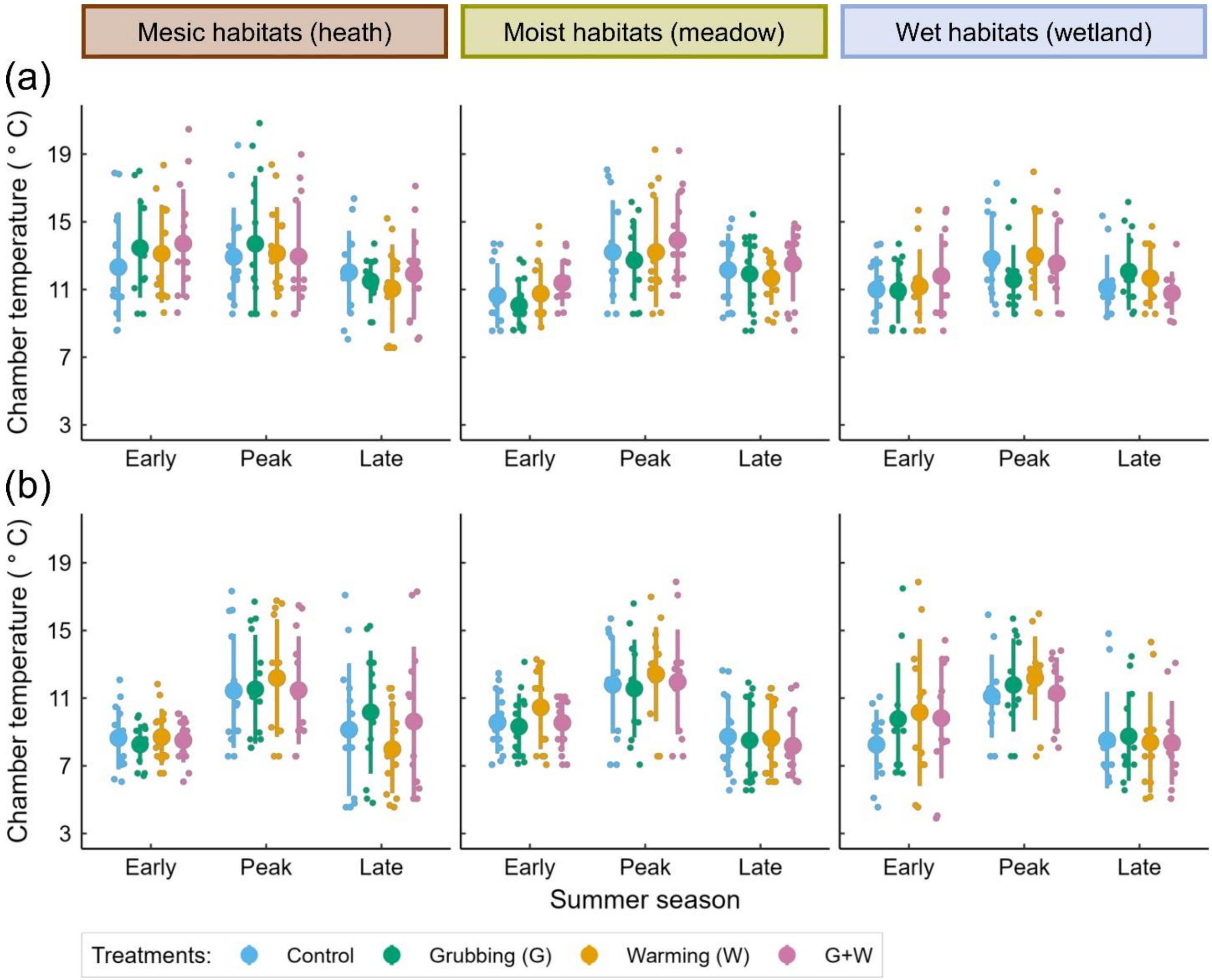

FIGURE S10. Differences in chamber air temperature among treatment and habitat CO_2-flux measurements were small. Average (± standard deviation) air temperature during ecosystem respiration (dark) measurements in each treatment combination across the summer of **(a)** 2016 and **(b)** 2017, separately for the three habitats. Air temperature was registered every 10 s within the chamber concurrently with air CO_2 concentration (cf. main text). The dots in the background represent the average air temperature during each dark measurement. Very similar patterns held for air temperature registered during net ecosystem exchange (light) measurements (data not shown).

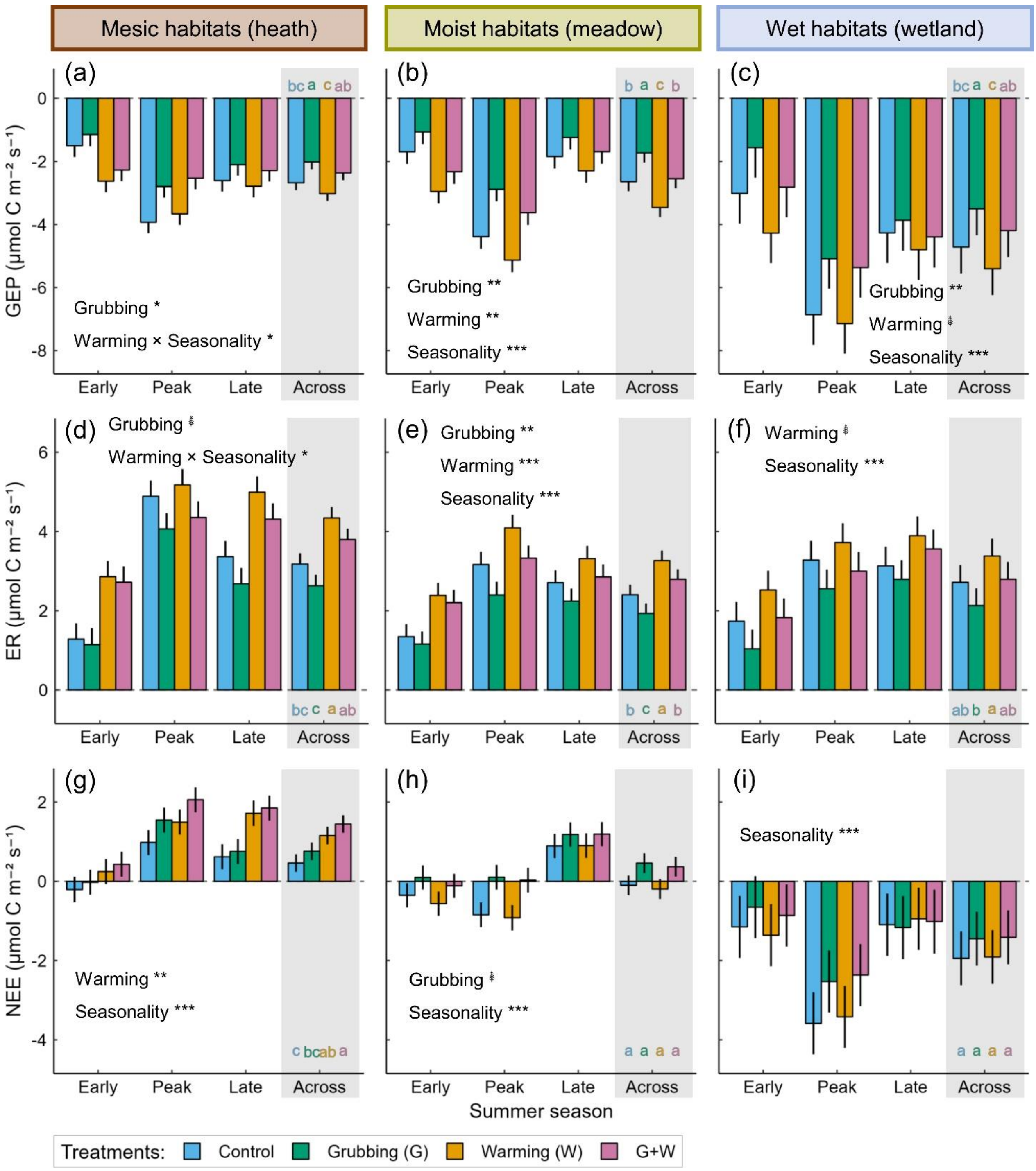

FIGURE S11. Effects of spring goose grubbing and summer warming on ecosystem CO_2-fluxes in 2016. Model predictions ± standard error (SE) for **(a-c)** gross ecosystem productivity (GEP), **(d-f)** ecosystem respiration (ER), and **(g-i)** net ecosystem exchange (NEE) in early, peak, and late summer, separately for the three habitats. Grey panels show model predictions ± SE averaged over the summer; different letters indicate significant

differences between treatments. Significant and marginally significant main and interactive effects are shown (ANOVA); when an interaction was significant (*P*<0.05), its main effects are not shown. Significance: ǂ*P*<0.1, **P*<0.05, ***P*<0.01, and ****P*<0.001. Full ANOVA results are given in Appendix S1: Table S8. LMM parameter estimates are given in Appendix S1: Tables S9-S11. Positive and negative fluxes denote CO_2 losses (the ecosystem acts as a C source) and CO_2 gains (the ecosystem acts as a C sink), respectively.

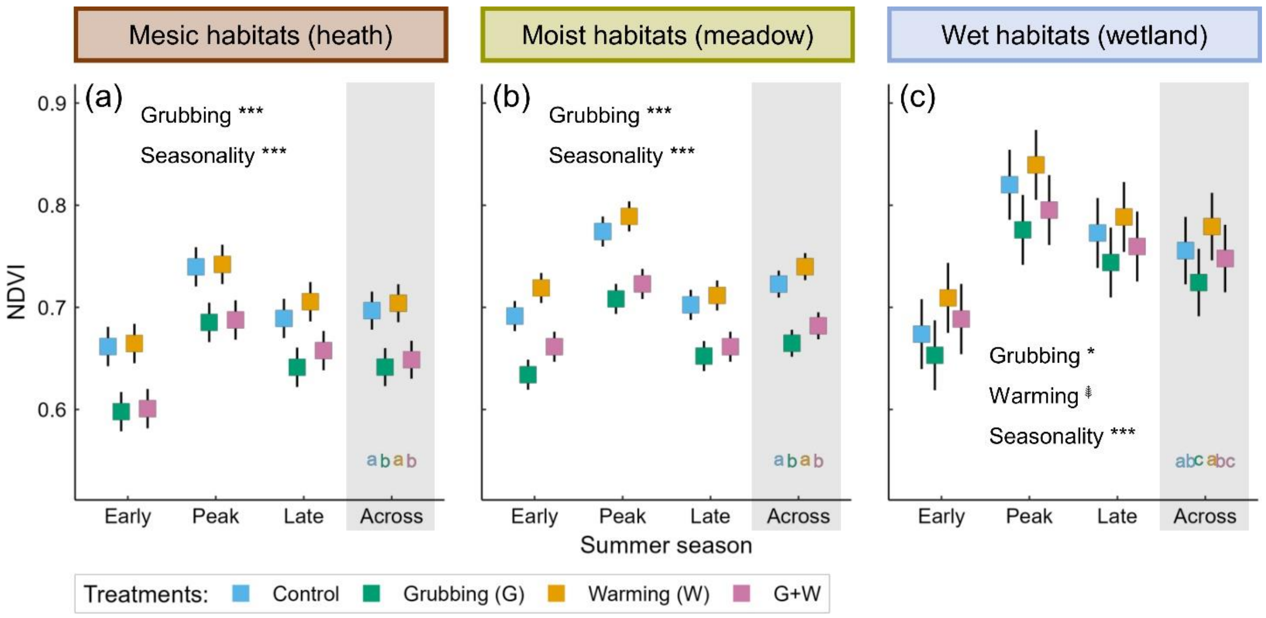

FIGURE S12. Effects of spring goose grubbing and summer warming on normalized-difference vegetation index (NDVI) in 2016. Model predictions ± standard error (SE) for NDVI of **(a)** mesic, **(b)** moist, and **(c)** wet habitats in early, peak, and late summer. Grey panels show model predictions ± SE averaged over the summer; different letters indicate significant differences between treatments. Significant and marginally significant main and interactive effects are shown (ANOVA); when an interaction was significant (*P*<0.05), its main effects are not shown. Significance: ⸸*P*<0.1, **P*<0.05, and ****P*<0.001. Full ANOVA results are given in Appendix S1: Table S8. LMM parameter estimates are given in Appendix S1: Table S12.

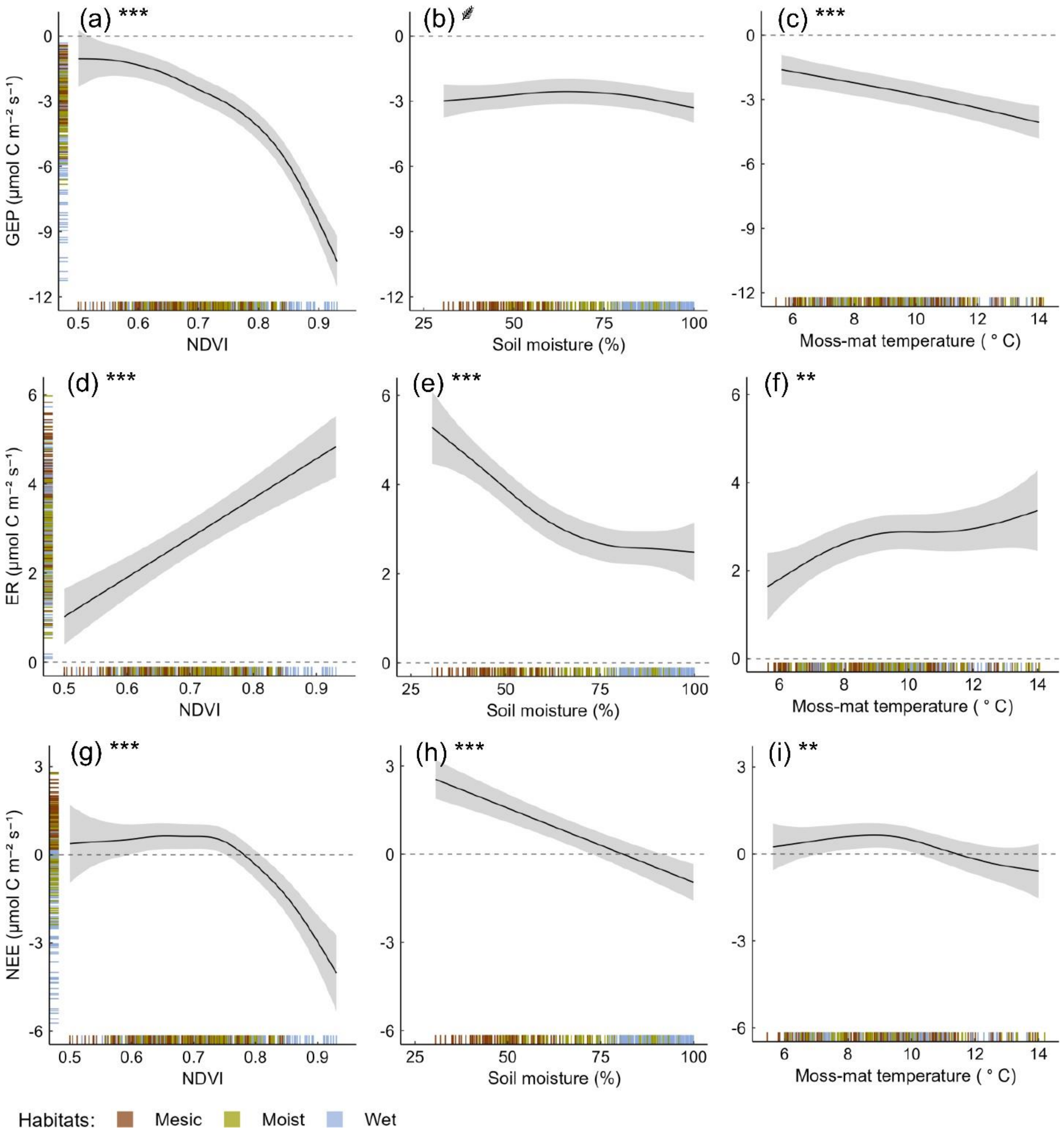

FIGURE S13. Across-habitat relationships between ecosystem CO_2-fluxes and both vegetation and abiotic variables in 2016. Regression lines ± 95% confidence interval (CI) for relations between **(a-c)** gross ecosystem productivity (GEP), **(d-f)** ecosystem respiration (ER), and **(g-i)** net ecosystem exchange (NEE) and the predictors (as additive smooth terms) normalized-difference vegetation index (NDVI), soil moisture, and moss-mat temperature (–2 cm). For each of the three models (GEP, ER, and NEE), the relationship with each predictor is shown at the average value of the other two predictors. Adjusted R^2 for each model: GEP:

0.63; ER: 0.33; NEE: 0.49. Rugs on the *y*-axis show predicted values, whereas rugs on the *x*-axis show raw values of the predictors (all colored according to habitat). Significance of the smooth terms: ǂP<0.1, **P<0.01, and ***P<0.001. ANOVA results are given in Appendix S1: Table S13. Positive and negative fluxes denote CO_2 losses (the ecosystem acts as a C source) and CO_2 gains (the ecosystem acts as a C sink), respectively. CO_2-flux relationships with air (+10 cm) and soil (−7 cm) temperatures (instead of moss-mat temperature) were very similar (not shown), plausibly because of the positive correlations among plot-level temperatures (Appendix S1: Figure S14).

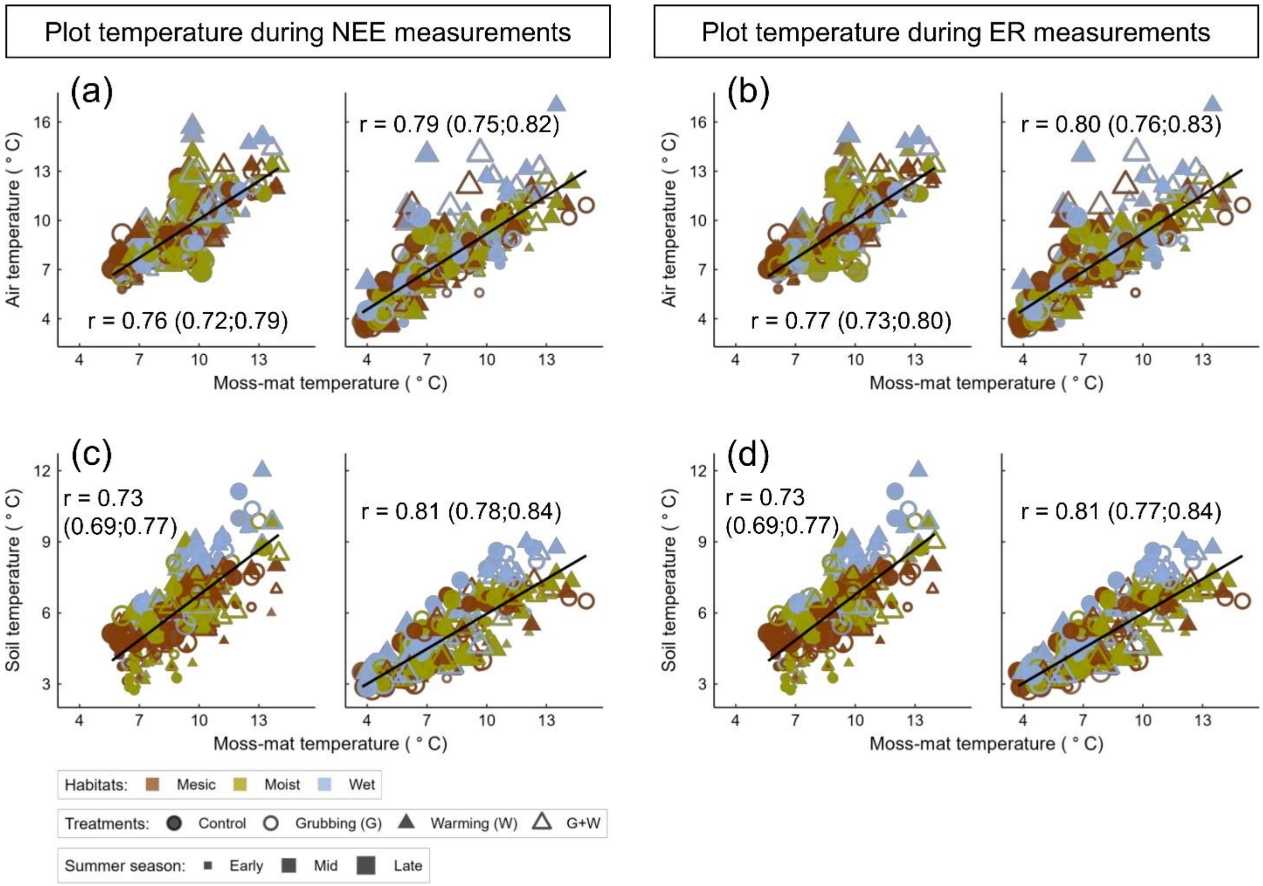

FIGURE S14. Relationships between plot-level temperatures during CO_2-flux measurements. Relationships between **(a)** moss-mat temperature (–2 cm) and air temperature (+10 cm) during net ecosystem exchange (NEE) measurements, **(b)** moss-mat temperature and air temperature during ecosystem respiration (ER) measurements, **(c)** moss-mat temperature and soil temperature (–7 cm) during NEE measurements, and **(d)** moss-mat temperature and soil temperature during ER measurements, separately for 2016 (left sub-panel) and 2017 (right sub-panel). Habitats, treatments, and summer season were used as coding variables. Pearson correlation coefficients (r) and their 95% confidence intervals for the relationships are given in the figure.

Section S1: ***Details on the non-metric multidimensional scaling presented in Figure 1 (main text).***

A non-metric multidimensional scaling (NMDS; Legendre & Legendre, 2012) was performed to help the reader visualize differences in plant-community composition and abiotic characteristics among the three studied habitats (mesic [heath vegetation], moist [meadow vegetation], and wet [wetland vegetation] habitats); see also Appendix S1: Figure S2. In this supporting information section, we describe data collection and analyses underlying the NMDS. Further details on data collection can also be found in Petit Bon et al. (2021) and Petit Bon et al. (2023a). Analyses were conducted in R v. 4.3.0 (https://www.r-project.org). Details on R packages and associated built-in R functions used here are given in the text below.

We determined live biomass in each plot (n = 80; see main text) using point intercept frequency methodology (PIM; Bråthen & Hagberg, 2004). To incorporate some of the within-plot spatial variation, two subplots (25 × 25 cm) were randomly selected within each plot and PIM was performed within these at peak growing season (end of July) in 2017 by using a sampling frame (25 × 25 cm area × 35 cm height) with 25 evenly distributed points. A pin density of 25 pins per ~0.06 m^2 area (the area of the subplots), which corresponds to a pin density of ~400 pins per 1 m^2 area, is largely above (i) the threshold after which an increase in number of pins has a negligible effect on the accuracy of biomass estimates (Bråthen & Hagberg, 2004) and (ii) the pin density used by the International Tundra Experiment (ITEX) network, a well-established multi-site (and multi-year) coordinated study examining the impacts of experimental warming on tundra ecosystems (Molau & Mølgaard, 1996; Henry et al., 2022). The two subplots at each plot in which PIM was performed are also the two subplots in which we gathered data for the present study (see *Material and Methods: Data collection and processing* in the main text for details).

Within each subplot, we vertically lowered a stick (3 mm in diameter) at all points and counted the number of hits with vascular plant live tissue, separately for each species. Point intercept data for each species (or genera when the species were not further identified; e.g. *Draba* spp.) were averaged between the two subplots within a plot and converted into live biomass (g m^{-2}) using the correlation coefficients in Petit Bon et al. (2021). Briefly, correlation coefficients were obtained by calculating the relationship between PIM data and live biomass data, separately for each plant functional type (*sensu* Chapin et al., 1996; forbs, grasses, sedges, rushes, deciduous dwarf shrubs, evergreen dwarf shrubs, and horsetails), collected from 17 external plots selected for destructive harvesting across the three studied habitats. For additional details, see Petit Bon et al. (2021). For a similar approach, see e.g., Ravolainen et al. (2010).

The NMDS was implemented using the *metaMDS*-function via '*vegan*' package (Oksanen et al., 2020) and was based on Bray-Curtis dissimilarity distances of the *log*-transformed aboveground relative plant species composition of the species/genera at each plot. A visual screening of the plant communities clearly reveals that species composition (Figure 1b), as well as aboveground live biomass of main growth forms (Figure 1c), varied widely between the three studied habitats. We statistically tested for differences in species composition among the three plant communities by using Permutational Multivariate Analysis of Variance (PERMANOVA; *adonis*-function via '*vegan*' package) based on the Bray-Curtis distance matrix of species composition (see above). We run PERMANOVA with 10000 restricted permutations (defined using the *how*-function via '*permute*' package) to account for the hierarchical spatial structure of the study design. In particular, 'plots' were allowed to permute only within the 'site' (seven in total) they belonged to. PERMANOVA results are reported in the table below.

Model terms	df	SS	MS	F-model	R^2	*P-value*
Habitat	2	8.344	4.172	51.911	0.574	**0.0001**
Residuals	77	6.188	0.080		0.426	
Total	79	14.532	0.184		1.000	

Abbreviations: df = degrees of freedom; SS = sum of squares; MS = mean of squares; F-model = pseudo-F statistics; R^2 = explained variance.

Finally, the statistical significance of soil environmental characteristics (moisture, pH, nitrogen [N] concentration, carbon [C] concentration, and C-to-N ratio) when *a posteriori* fitted onto the NMDS ordination was evaluated by also using 10000 restricted permutations to account for the hierarchical spatial structure of the study design (*envfit*-function via '*vegan*' package; see above). While soil moisture data were specifically collected for the present study (see *Material and Methods: Data collection and processing* in the main text for details; here we only used data that were collected at peak summer), collection of peak summer data for soil pH, N concentration, C concentration, and C-to-N ratio is described in Petit Bon et al. (2021) and Petit Bon et al. (2023a).

The interpretation of significant soil environmental features (vectors), which were soil moisture, soil N concentration, and soil C concentration, is that they significantly correlate with the Bray-Curtis distance matrix of species composition (see above). As such, the direction of the vectors in the NMDS ordination space indicates towards which direction environmental characteristics shift most rapidly and the direction to which they have maximal correlations with the ordination configuration. The projections of points (i.e. experimental plots) onto vectors have maximum correlation with corresponding soil environmental characteristics.

Section S2: ***Additional details on data analyses.***

In this supporting information section, we provide additional details on data exploration and data analyses, aiming at clarifying modelling strategy and analytical decisions, following guidelines for a greater level of result interpretation, reproducibility, and transparency (Borregaard & Hart, 2016; Calin-Jageman & Cumming, 2019; Ioannidis, 2019). Data exploration and analyses were conducted in R v. 4.3.0 (https://www.r-project.org). Details on R packages and associated built-in R functions used here are given in the text below.

Data exploration

Following standardized protocols (Zuur et al., 2010), and before formal analyses (modelling; see next section), we performed (graphical) data exploration to (i) identify unreliable data points and (ii) detect extreme observations (outliers). To achieve this, we used a variety of graphical tools (histograms, Cleveland dotplots, boxplots, scatterplots, etc.; Ieno & Zuur, 2015) using the *ggplot*-function via '*ggplot2*' package (Wickham, 2016). These same graphical tools were also employed to initially detect biologically meaningful patterns in raw data. The different steps of data exploration and associated decisions are reported below.

First, we removed 23 CO_2-flux data points (~0.9% of the total 2427 net ecosystem exchange [NEE] and ecosystem respiration [ER] measures) for which clear errors had occurred during data collection (e.g. the tubing connecting the gas analyzer to the chamber got disconnected during measurements), and gross ecosystem productivity (GEP) was not calculated in these instances. In particular, 7 of these data points belonged to the 2016-dataset and 16 of these data points (3 of which from naturally grubbed plots) belonged to the 2017-dataset. Seen differently, 20 of these data points were NEE (light) measures and 3 of these data points were ER (dark) measures. Analogously, we excluded 1 normalized-difference vegetation index (NDVI) data point (~0.1% of the total 960 NDVI measures) for which a clear error had

occurred during measurements (the NDVI, which values are constrained between 0 and 1, was negative).

Second, as ER and GEP can almost equal, it is not surprising that NEE may take values of ~0 µmol CO_2 m^{-2} s^{-1}, and for which the R^2 of the linear regression model is inherently low. Similarly low NEE values, and hence low R^2 of the linear regression models, may also be detected when main ecosystem fluxes (ER and GEP) are very low, which is not uncommon in temperature-, nutrient-limited Arctic ecosystems. Therefore, we still retained 100 NEE data points (~8.4% of the total remaining 1195 NEE measures after error removal; see above) for which the R^2 of the linear regression model was relatively low ($R^2 < 0.70$), but there was otherwise no indication that errors had occurred. Nonetheless, the R^2 of the linear regression models clearly indicated that CO_2-flux measurements were of high quality (details in the table below).

Measurement quality	Average ± standard deviation				Median			
	Mesic habitats	Moist habitats	Wet habitats	Overall	Mesic habitats	Moist habitats	Wet habitats	Overall
R^2 NEE measurements (all)	0.90 ± 0.21	0.86 ± 0.25	0.87 ± 0.27	0.88 ± 0.24	0.96	0.95	0.98	0.96
R^2 NEE measurements (with R^2 > 0.70)	0.947 ± 0.052	0.943 ± 0.055	0.957 ± 0.052	0.949 ± 0.053	0.97	0.96	0.98	0.97
R^2 ER measurements (all)	0.985 ± 0.019	0.989 ± 0.013	0.988 ± 0.016	0.987 ± 0.016	0.991	0.993	0.993	0.993

Notes: average and median R^2 of NEE measurements are also presented separately when the 100 NEE data points with $R^2 < 0.70$ were excluded.

Third, to increase precision of parameter estimates, we excluded 24 outliers (~0.7% of the total remaining 3596 NEE, ER, and GEP measures after error removal; see above), which were defined as values outside the range of ±3 standard deviations from their group average, i.e., from the average of the 'year × habitat × treatment × sampling occasion' group they belonged to (cf. Benhadi-Marín, 2018). For completeness, we also ran all the analyses (see main text and next section for details) by including these outliers, and biologically meaningful patterns did not differ from those detected in the absence of outliers (results not

shown). An overview of the final dataset used in the analyses is given in Appendix S1: Table S1. The R-script used to process CO_2-flux data and perform the data exploration described above is given in Petit Bon et al. (2023b).

Finally, because we excluded a total of 47 CO_2-flux data points (23 data points associated with clear errors that had occurred during data collection and 24 outliers; see above), we explored possibly introduced non-random trends in missing observations using the suite of functions offered by the '*naniar*' package (Tierney & Cook, 2023). No patterns were identified that could affect the estimates of the models and hence modify our biological conclusions. This exploration was not performed on NDVI data, as a single data point was excluded there (see above).

Modelling strategy and analytical decisions

We designed the model structure of our main linear mixed-effects models (LMMs) following the major experimental question asked by this study. As we set out to investigate the extent to which spring goose grubbing and summer warming, either alone or in combination, modify high-Arctic ecosystem CO_2-fluxes during the growing season, we fitted LMMs in which the initial full fixed-effects structure included the three-way interaction 'grubbing × warming × growing season'. We refrained from fitting more complex models, such as the ones including the four-way interaction 'grubbing × warming × growing season × habitat', as the many estimated interaction coefficients from these models could not be interpreted with confidence. We decided to fit separate LMMs for the two years of study (2016 and 2017) for two main reasons: (i) including 'year' as an additional fixed-effect would have also introduced a potential four-way interaction in the models and (ii) interpreting an eventual interaction between 'year' and treatments is not necessarily meaningful, as the one-year and two-year responses to treatments would be completely confounded with that these responses are being

evaluated in two years that inherently differ in both biotic and abiotic conditions. After pondering the possibility of including ‘year’ as a temporally crossed random-effects to the spatial structure of the experiment (cf. Baayen et al., 2008; Petit Bon et al., 2022), we did not do so as two levels (here ‘year 2016’ and ‘year 2017’) for a random-effect are often not sufficient to properly determine its variance component (Bolker et al., 2009).

To be able to compare effect sizes of treatment effects across habitats and years, we retained the most parsimonious, yet common, fixed-effects structure for all the analyses (cf. Ravolainen et al., 2011; Petit Bon et al., 2023a). Indeed, different fixed-effects structures inherently differ in the way that model variance is partitioned across the specified fixed-effect coefficients, thus making a direct comparison between effect sizes obtained in different models practically impossible. Yet, as interactions in the models influence the variance attributed to the main-effects, we also ran all the analyses by obtaining the most parsimonious models. Because the selected structure did not significantly affect the estimates of the models, and hence did not modify our biological conclusions, we confidently retained a common fixed-effects structure for all the analyses to be able to compare (i) effect sizes of CO_2-flux responses to treatments within and across habitats, as well as of one-year and two-year responses, and (ii) CO_2-flux and NDVI responses. Model simplification was achieved through analysis of variance (ANOVA) using the *anova*-function. We also performed model simplification by using likelihood ratio tests, and obtained identical results.

We separately selected the better random-effects structure for each model by deleting those terms with an estimated zero variance. However, given the fervent debate around the simplification of the random-effects structure (Barr et al., 2013; Bates et al., 2015), we also ran all the analyses by maintaining the maximal random-effects structure (i.e. all the random intercepts) in each LMM. For those models that converged with a maximal random-effects

structure (some models did have convergence issues indeed), this latter did not affect the estimates of the fixed-effects compared to models with a simplified random-effects structure. As such, we decided to present LMMs with the reduced random-effects structure, as suggested by Bates et al. (2015). The selection of the better random-effects structure for each LMM was accomplished by using a combination of two alternative methods: (i) parametric bootstrapping with 10000 replicates using the *confint*-function and (ii) graphical tools to visually inspect the variability of each random-effect.

We used additive mixed-effects models (AMMs) to gain a better mechanistic understanding of the biotic and abiotic controls of CO_2-fluxes in this high-Arctic ecosystem. We built across-habitat models by incorporating three key predictors that have been repeatedly shown to explain a significant amount of variation in CO_2-fluxes: normalized-difference vegetation index (NDVI; e.g., Boelman et al., 2003; Virkkala et al., 2024), which reflects net primary productivity and plant biomass (Appendix S1: Figure S6), soil moisture content (e.g., Sjögersten et al., 2006; Zona et al., 2022), and temperature (e.g., Lund et al., 2012; Virkkala et al., 2024). In these models, we did not include 'grubbing' and/or 'warming' as fixed-effects as they would be largely correlated with the considered predictors (e.g., grubbing reduced NDVI [Figure 3; Appendix S1: Figures S12], while warming increased plot temperatures [Appendix S1: Figures S3-S5]). Yet, in the interpretation of the results obtained from these models, it is important to notice that both grubbing and warming were important modifiers of the biotic and abiotic environment.

In these AMMs, the nested spatial structure of the experiment was specified as random-effects part of the models (cf. the LMMs described above). Random-effects were fitted as smooth terms, i.e. penalized regression terms, following Wood (2017), and the better random-effects structure for each AMM was obtained by removing those terms that were not

statistically significant ($P > 0.05$). When compared to the habitat-specific LMMs (see above), these across-habitat AMMs initially included the additional, highest-ranked random-effect 'Site', which was specified to account for the fact that three blocks (one for each habitat) were selected at each of the 7 sites.

REFERENCES

Baayen, R. H., D. J. Davidson, and D. M. Bates. 2008. “Mixed-effects modeling with crossed random effects for subjects and items.” *Journal of Memory and Language* 59: 390-412.

Barr, D. J., R. Levy, C. Scheepers, and H. J. Tily. 2013. “Random effects structure for confirmatory hypothesis testing: Keep it maximal.” *Journal of Memory and Language* 68: 255-278.

Bates, D., R. Kliegl, S. Vasishth, and H. Baayen. 2015a. “Parsimonious mixed models.” arXiv preprint arXiv: 1506.04967.

Benhadi-Marín, J. 2018. “A conceptual framework to deal with outliers in ecology.” *Biodiversity and Conservation* 27: 3295-3300.

Boelman, N. T., M. Stieglitz, H. M. Rueth, M. Sommerkorn, K. L. Griffin, G. R. Shaver, and J. A. Gamon. 2003. “Response of NDVI, biomass, and ecosystem gas exchange to long-term warming and fertilization in wet sedge tundra.” *Oecologia* 135: 414-421.

Bolker, B. M., M. E. Brooks, C. J. Clark, S. W. Geange, J. R. Poulsen, M. H. H. Stevens, and J.-S. S. White. 2009. “Generalized linear mixed models: a practical guide for ecology and evolution.” *Trends in Ecology & Evolution* 24: 127-135.

Borregaard, M. K., and E. M. Hart. 2016. “Towards a more reproducible ecology.” *Ecography* 39: 349-353.

Bråthen, K. A., and O. Hagberg. 2004. “More efficient estimation of plant biomass.” *Journal of Vegetation Science* 15: 653-660.

Calin-Jageman, R. J., and G. Cumming. 2019. “The New Statistics for better science: Ask how much, how uncertain, and what else is known.” *The American Statistician* 73: 271-280.

Chapin, F. S., M. S. Bret-Harte, S. E. Hobbie, and H. Zhong. 1996. “Plant functional types as predictors of transient responses of arctic vegetation to global change.” *Journal of Vegetation Science* 7: 347-358.

Henry, G. H., R. D. Hollister, K. Klanderud, R. G. Björk, A. D. Bjorkman, C. Elphinstone, I. S. Jónsdóttir, U. Molau, A. Petraglia, and S. F. Oberbauer. 2022. “The International Tundra Experiment (ITEX): 30 years of research on tundra ecosystems.” *Arctic Science* 8: 550-571.

Ieno, E. N., and A. F. Zuur. 2015. “A Beginner's guide to data exploration and visualisation with R.” Highland Statistics Ltd.

Ioannidis, J. P. 2019. “What have we (not) learnt from millions of scientific papers with P values?” *The American Statistician* 73: 20-25.

Legendre, P., and L. Legendre. 2012. “Numerical ecology.” Elsevier Science, Amsterdam, NL.

Lund, M., J. M. Falk, T. Friborg, H. N. Mbufong, C. Sigsgaard, H. Soegaard, and M. P. Tamstorf. 2012. “Trends in CO2 exchange in a high Arctic tundra heath, 2000–2010.” *Journal of Geophysical Research: Biogeosciences* 117: G02001.

Molau, U., and P. Mølgaard. 1996. “International tundra experiment (ITEX) manual.” Danish Polar Center, Copenhagen, Denmark.

Oksanen, J., F. G. Blanchet, M. Friendly, R. Kindt, P. Legendre, D. McGlinn, P. R. Minchin, R. B. O'Hara, G. L. Simpson, P. Solymos, M. H. H. Stevens, E. Szoecs, and H. Wagner. 2020. “vegan: Community Ecology Package.” R package version 2.5-7. https://CRAN.R-project.org/package=vegan.

Petit Bon, M., H. Böhner, K. A. BrÅthen, V. T. Ravolainen, and I. S. Jónsdóttir. 2021. “Variable responses of carbon and nitrogen contents in vegetation and soil to herbivory and warming in high-Arctic tundra.” *Ecosphere* 12 :e03746.

Petit Bon, M., K. G. Inga, T. A. Utsi, I. S. Jónsdóttir, and K. A. Bråthen. 2022. "Forage quality in tundra grasslands under herbivory: Silicon-based defences, nutrients and their ratios in grasses." *Journal of Ecology* 110: 129-143.

Petit Bon, M., K. A. Bråthen, V. T. Ravolainen, G. Ottaviani, H. Böhner, and I. S. Jónsdóttir. 2023a. "Herbivory and warming have opposing short-term effects on plant-community nutrient levels across high-Arctic tundra habitats." *Journal of Ecology* 111: 1514-1530.

Petit Bon, M., B. B. Hansen, M. J. J. E. Loonen, A. Petraglia, K. A. Bråthen, H. Böhner, K. Layton-Matthews, K. H. Beard, M. Le Moullec, I. S. Jónsdóttir, and R. Van der Wal. 2023b. "Long-term herbivore removal experiments reveal how geese and reindeer shape vegetation and ecosystem CO2-fluxes in high-Arctic tundra." *Journal of Ecology* 111: 2627-2642.

Ravolainen, V. T., K. A. Bråthen, R. A. Ims, N. G. Yoccoz, J.-A. Henden, and S. T. Killengreen. 2011. "Rapid, landscape scale responses in riparian tundra vegetation to exclusion of small and large mammalian herbivores." *Basic and Applied Ecology* 12: 643-653.

Ravolainen, V. T., N. G. Yoccoz, K. A. Bråthen, R. A. Ims, M. Iversen, and V. T. González. 2010. "Additive partitioning of diversity reveals no scale-dependent impacts of large ungulates on the structure of tundra plant communities." *Ecosystems* 13: 157-170.

Sjögersten, S., R. Van der Wal, and S. Woodin. 2006. "Small-scale hydrological variation determines landscape CO2 fluxes in the high Arctic." *Biogeochemistry* 80: 205-216.

Tierney, N., and D. Cook. 2023. "Expanding tidy data principles to facilitate missing data exploration, visualization and assessment of imputations." *Journal of Statistical Software* 105: 1-31.

Virkkala, A. M., P. Niittynen, J. Kemppinen, M. E. Marushchak, C. Voigt, G. Hensgens, J. Kerttula, K. Happonen, V. Tyystjärvi, C. Biasi, J. Hultman, J. Rinne, and M. Luoto. 2024. "High-resolution spatial patterns and drivers of terrestrial ecosystem carbon dioxide, methane, and nitrous oxide fluxes in the tundra." *Biogeosciences* 21: 335-355.

Wickham, H. 2016. "ggplot2: elegant graphics for data analysis." Springer.

Wood, S. N. 2017. "Generalized additive models: an introduction with R." CRC press.

Zona, D., P. M. Lafleur, K. Hufkens, B. Gioli, B. Bailey, G. Burba, E. S. Euskirchen, et al. 2022. "Pan-Arctic soil moisture control on tundra carbon sequestration and plant productivity." *Global Change Biology* 29: 1267-1281.

Zuur, A. F., E. N. Ieno, and C. S. Elphick. 2010. "A protocol for data exploration to avoid common statistical problems." *Methods in Ecology and Evolution* 1: 3-14.
